# *In silico* identification of modulators of J domain protein-Hsp70 interactions in *Plasmodium falciparum*: a drug repurposing strategy against malaria

**DOI:** 10.3389/fmolb.2023.1158912

**Published:** 2023-08-09

**Authors:** Harpreet Singh, Shaikha Y. Almaazmi, Tanima Dutta, Robert A. Keyzers, Gregory L. Blatch

**Affiliations:** ^1^ Department of Bioinformatics, Hans Raj Mahila Maha Vidyalaya, Jalandhar, India; ^2^ Biomedical Research and Drug Discovery Research Group, Faculty of Health Sciences, Higher Colleges of Technology, Sharjah, United Arab Emirates; ^3^ Department of Diagnostic Genomics, Path West Nedlands, QEII Medical Centre, Nedlands, WA, Australia; ^4^ Centre for Biodiscovery & School of Chemical and Physical Sciences, Victoria University of Wellington, Wellington, New Zealand; ^5^ Biomedical Biotechnology Research Unit, Department of Biochemistry and Microbiology, Rhodes University, Grahamstown, South Africa

**Keywords:** Hsp70, J domain protein, pandemic response box, virtual screening, drug repurposing

## Abstract

*Plasmodium falciparum* is a unicellular, intracellular protozoan parasite, and the causative agent of malaria in humans, a deadly vector borne infectious disease. A key phase of malaria pathology, is the invasion of human erythrocytes, resulting in drastic remodeling by exported parasite proteins, including molecular chaperones and co-chaperones. The survival of the parasite within the human host is mediated by *P. falciparum* heat shock protein 70s (PfHsp70s) and J domain proteins (PfJDPs), functioning as chaperones-co-chaperones partnerships. Two complexes have been shown to be important for survival and pathology of the malaria parasite: PfHsp70-x-PFE0055c (exported); and PfHsp70-2-PfSec63 (endoplasmic reticulum). Virtual screening was conducted on the drug repurposing library, the Pandemic Response Box, to identify small-molecules that could specifically disrupt these chaperone complexes. Five top ranked compounds possessing preferential binding affinity for the malarial chaperone system compared to the human system, were identified; three top PfHsp70-PfJDP binders, MBX 1641, zoliflodacin and itraconazole; and two top J domain binders, ezetimibe and a benzo-diazepinone. These compounds were validated by repeat molecular dockings and molecular dynamics simulation, resulting in all the compounds, except for MBX 1461, being confirmed to bind preferentially to the malarial chaperone system. A detailed contact analysis of the PfHsp70-PfJDP binders identified two different types of modulators, those that potentially inhibit complex formation (MBX 1461), and those that potentially stabilize the complex (zoliflodacin and itraconazole). These data suggested that zoliflodacin and itraconazole are potential novel modulators specific to the malarial system. A detailed contact analysis of the J domain binders (ezetimibe and the benzo-diazepinone), revealed that they bound with not only greater affinity but also a better pose to the malarial J domain compared to that of the human system. These data suggested that ezetimibe and the benzo-diazepinone are potential specific inhibitors of the malarial chaperone system. Both itraconazole and ezetimibe are FDA-approved drugs, possess anti-malarial activity and have recently been repurposed for the treatment of cancer. This is the first time that such drug-like compounds have been identified as potential modulators of PfHsp70-PfJDP complexes, and they represent novel candidates for validation and development into anti-malarial drugs.

## 1 Introduction

The parasite *Plasmodium falciparum*, which causes a severe form of malaria in humans, encodes 49 J domain proteins (PfJDPs), of which 18 are predicted to be exported into host cells during the intracellular erythrocytic stage of the life cycle (Dutta et al., 2021a; Almaazmi et al., 2022). The PfJDPs play an important role in the establishment of infection in the human host, serving as co-chaperones for the activation of heat shock protein 70 kDa (Hsp70) molecular chaperones of both parasite and human origin. The interaction of these exported PfJDPs with Hsp70s enables the trafficking, folding and functionalization of key virulence factors (e.g. *P. falciparum* erythrocyte membrane protein 1, PfEMP1) thereby augmenting malaria pathogenicity (Külzer et al., 2012; Behl et al., 2019). The exported PfJDP, PFA0660w (PlasmoDB ID: PF3D7_0113,700), stimulates the ATPase activity of the only exported *P. falciparum* Hsp70, PfHsp70-x (Daniyan et al., 2016). In addition, PFA0660w associates with another exported PfJDP (PFE0055c; PlasmoDB ID: PF3D7_0501,100) and PfHsp70-x in J-dots, highly mobile structures found in the cytosol of parasite-infected erythrocytes, and implicated in the trafficking of PfEMP1 (Külzer et al., 2010; Külzer et al., 2012; Petersen et al., 2016; Behl et al., 2019). PFE0055c is essential to parasite survival (Zhang et al., 2018), and stimulates the basal ATPase activity of PfHsp70-x to a greater extent than PFA0660w (Dutta et al., 2021b). Furthermore, small-molecule inhibition assays have revealed that the chalcone, C86, preferentially inhibits the PFE0055c-stimulated ATPase activity of PfHsp70-x (Dutta et al., 2021b).

Another potential anti-malarial drug target is the endoplasmic reticulum (ER)-resident chaperone, *P. falciparum* Hsp70-2 (PfHsp70-2). PfHsp70-2 is essential to parasite survival (Zhang et al., 2018), and is proposed to associate with the ER-resident PfJDP, *P. falciparum* Sec63 (PfSec63; PlasmoDB ID: PF13_0102/PF3D7_1318800), corresponding to the homologous human partnership of *Homo sapiens* GRP78 (HsGRP78)-*Homo sapiens* Sec63 (HsSec63) involved in protein translocation into the ER (Tuteja, 2007; Zimmermann and Blatch, 2009; Cortés et al., 2020; Blatch, 2022). Interestingly, like PfHsp70-x, PfHsp70-2 also interacts with exported proteins involved in pathogenesis, including PfEMP1 (Saridaki et al., 2008; Batinovic et al., 2017; Cortés et al., 2020). We propose that the PfHsp70-x-PFE0055c and PfHsp70-2-PfSec63 partnerships are important components of the malaria parasite protein export pathway, and are therefore key drug targets for the identification of novel chaperone inhibitors and potential anti-malarial drugs.

The advent of publicly available drug repurposing libraries has provided an opportunity to apply virtual screening approaches to identify novel inhibitors with validated drug-like properties. A prime example of such a library is the Pandemic Response Box (PRB), which is a collection of approximately 400 small molecule compounds with demonstrated potent anti-viral, anti-bacterial, anti-fungal or anti-neoplastic activity (Samby et al., 2022). Therefore, the aim of this study was a bioinformatics analysis of the interaction of PFE0055c and PfSec63 with their corresponding PfHsp70s, and a molecular docking-based screening of the drug-repurposing PRB library. In addition, comparative side-by-side studies were carried out with the corresponding human Hsp70-JDP systems to identify selective inhibitors of the novel PfHsp70-JDP complexes.

## 2 Materials and methods

### 2.1 Retrieval of sequences and protein crystal structures

Sequences of *P. falciparum* proteins of interest were retrieved from PlasmoDB (https://plasmodb.org). The amino acid sequences of the human and *Escherichia coli* proteins of interest were retrieved from Uniprot (https://www.uniprot.org). To carry out various protein-protein and protein-ligand interaction studies, protein crystal structures of the Hsp70 Nucleotide Binding Domains (NBDs) and DNAJA1 J domain were obtained from the Protein Data Bank (PDB, https://www.rcsb.org/; Table 1). However, the crystal structure of the PfHsp70-x NBD (PDB ID: 6S02) was remodeled to accommodate missing residues (in position 217-221) based on the *E. coli* DnaK (EcDnaK) crystal structure (PDB ID: 5NRO) (Kityk et al., 2018). The comparative modelling of the PFE0055c, PfSec63 and HsSec63 J domains were performed with *E. coli* DnaJ (EcDnaJ) (PDB ID: 5NRO) (Kityk et al., 2018) as a template for the PfJDPs, and *Mus musculus* (mouse) DnaJ (PDB ID: 2CUG) as a template for HsSec63. All the above modeling procedures were carried out using Modeller Version 10.1 (Webb and Sali, 2016). The 3D models of PFE0055c, PfSec63 and HsSec63 J domains were also predicted using SWISS-MODEL (Waterhouse et al., 2018) and AlphaFold2 (Varadi et al., 2022) implemented in Google Colab (https://colab.research.google.com/). The models predicted with SWISS-MODEL as well as AlphaFold2 were almost identical to those predicted with Modeller except for some variations in the helix-IV region of the J domains predicted by AlphaFold2 (Supplementary Figure S1), prompting us to confidently use the 3D structures of the J domains predicted by Modeller in the following steps.

**TABLE 1:** Details of interacting residues in Hsp70 NBDs and JDP J domains.

**S. No.**	**Protein**	**Full sequence length, uniprot ID**	**Position of the NBD/J domain**	**Interacting residues**	**PDB ID**
**Hsp70 NBD**					
1	EcDnaK	638, P0A6Y8	1–385	206 (E), 211 (D), 217 (E)	5NRO
2	PfHsp70-x NBD	679, K7NTP5	25–414	243 (E), 244 (D), 248 (E)	6S02
3	HsHsp70 NBD	641, P0DMV8	1–383	213 (D), 214 (D), 218 (E)	1HJO
4	PfHsp70-2 NBD	652, Q8I2X4	26–404	235 (D), 236 (N), 240 (E)	5UMB
5	HsGRP78 NBD	654, P11021	27–407	238 (D), 239 (N), 243 (E)	5F2R
**JDP J domain**					
1	EcDnaJ J domain	376, P08622	3–72	22 (R), 26 (K), 27 (R)	5NRO
2	PFE0005c J domain	402, Q8I489	80–148	97 (K), 101 (R), 102(K)	Modeled using 5NRO
3	DNAJA1 J domain	397, P31689	6–68	23 (K), 27 (R), 28 (K)	2M6Y
4	HsSec63 J domain	760, Q9UGP8	129–194	146 (K), 150 (R), 151 (L)	Modeled using 2CUG
5	PfSec63 J domain	651, Q8IEC8	129–194	121 (K), 125 (R), 126 (L)	Modeled using 5NRO

Multiple sequence alignments were produced in Pearson/FASTA format using the online Clustal Omega server (https://www.ebi.ac.uk/Tools/msa/clustalo/) (Sievers and Higgins, 2017), and rendered with box shading specifying the fraction of residues that meet identity or similarity in each column of the alignment at a 50% consensus level using Multiple Align Show (http://www.bioinformatics.org/SMS/multi_align.html) (Stothard, 2000).

### 2.2 Retrieval of small molecule virtual library

The PRB library is provided by Medicines for Malaria Venture (MMV, Geneva, Switzerland) for virtual screening (Samby et al., 2022). The PRB website lists the compounds included in this virtual library (https://www.mmv.org/mmv-open). Compound file conversion to PDBQT format was carried out using Open Babel (http://openbabel.org) (O’Boyle et al., 2011).

### 2.3 Molecular docking

The NBD domains of PfHsp70-x, HsHsp70, and PfHSP70-2 were retrieved from PDB database (https://www.rcsb.org/) in the ADP-bound form, while the NBD of HsGRP78 was retrieved in the AMP-PCP (a nucleotide triphosphate analog)-bound form, and using the respective PDB IDs as mentioned in Table 1. Protein-protein docking of the J domains of PFE0055c and PfSec63 with their corresponding Hsp70 NBD partner was performed with the HADDOCK2.4 Server (https://wenmr.science.uu.nl/haddock2.4/) (van Zundert et al., 2016; Honorato et al., 2021) using default parameters, while specifying the interacting residues for both the members of the complexes in reference to the EcDnaK-EcDnaJ interface (Kityk et al., 2018). The validation of the complexes was accomplished by using the ClusPro 2.0 online server (Kozakov et al., 2013; Kozakov et al., 2017; Vajda et al., 2017; Desta et al., 2020) to generate complexes with which to compare to the HADDOCK complexes used in the docking screening (Supplementary Figure S2). The complexes were then evaluated for their similarity to validate the accuracy of the HADDOCK complexes used in the docking screening. Before proceeding to molecular docking studies, the 3D structures of Hsp70 NBD-JDP J domain complexes as well as the J domains were validated using the Saves v6.0 web server to assess the quality of the structures (Supplementary Table S1). Efficient protein-ligand molecular docking requires the specification of grids to achieve docking of ligand molecules at specific binding sites. MGLTools interactive graphical user interface was used to create grid files by specifying grid parameters and the center for the desired binding site for the molecular docking. The grid size parameters were used to efficiently cover the interface region of the Hsp70 NBD-JDP J domain complexes and helix II region of the J domains. The virtual screening of the PRB library was performed using AutoDock Vina version 1.2.0 (https://vina.scripps.edu) (Trott and Olson, 2010) using an in house created shell script. Analysis and sorting of the virtual screening log files was performed using a publically available python script (https://github.com/Bioinformatics-Review/VS-Analysis).

### 2.4 Analysis, validation and visualization of molecular structures

Analyses of the virtual screening results were carried out using visual inspection for the selection of only those docked poses docking into the interface region and making contact with the key residues (Table 1), and then ranking them based on their binding affinity as compared to the control, C86. The top binders were chosen based on their differential binding affinity, with the malarial system having a greater binding affinity as compared to the human system (a minimum difference of 0.6 kcal/mol, Supplementary Tables S2–S15 and S14–S16). The validation of the top docked ligands was carried out by re-docking the compounds three times using the same method, parameters and structures to determine the consistency of the result. The accuracy of the binding conformation was assessed by evaluating the interaction energies between the original and re-docked poses. In addition, detailed interaction plots were generated for the highest-scores of the ligand-protein interaction to differentiate between interacting and non-interacting or weakly interacting residues of all docking poses using LigPlot+ (https://www.ebi.ac.uk/thornton-srv/software/LigPlus/) (Laskowski and Swindells, 2011). The LigPlot + analysis also enabled the choice of conformations docked at the required interface, as well as validation of the re-docked conformations. UCSF chimera 1.10.1 molecular visualization tool (Pettersen et al., 2004) was used to visualize and render the graphical images of structural features and clusters of strongest docked conformations. The RMSD values between the various Hsp70-JDP J domain complexes in reference to the EcDnaK-EcDnaJ J domain complex were calculated using the MatchMaker tool implemented in the UCSF chimera 1.10.1. The RMSD is calculated based on the distances between the aligned pairs of the backbone C-alpha atoms in aligned/superimposed posed structures. A lower RMSD value indicates a better alignment between the pair of structures. The RMSD values (less than or equal to 1.0 Å) of the complexes used in this study as compared to the reference EcDnaJ-EcDnaK J domain complex indicated that they could be used to reproduce the required interaction interface. PyMol 2.5.2 (PyMOL Molecular Graphics System, Version 2.0 Schrödinger, LLC) was used to visualize and render the strongest docked conformations, amino acid residues forming hydrogen bond (H-bond) interactions and calculate the surface electrostatic potential of every protein using APBS Electrostatics Plugin (https://server.poissonboltzmann.org). Pharmacokinetic and drug likeness prediction of top binder compounds was conducted using the SwissADME online server (Daina et al., 2017) to evaluate the chemical and physical properties of the compounds and predict toxicity, absorption, distribution, metabolism and excretion.

### 2.5 Molecular dynamics simulation

Molecular Dynamics (MD) simulation of the top five screened compounds was performed to analyze the conformational behavior and stability of the binding pose using GROMACS version 2022.2 software package (Van Der Spoel et al., 2005) followed by Molecular Mechanics Poisson-Boltzmann (MMPBSA) free energy calculations and its decomposition using gmx_MMPBSA (Miller et al., 2012; Valdés-Tresanco et al., 2021). The topology and the force field parameters were derived from CHARMM36 using GROMACS for protein molecules/complexes and using CGenFF server for ligand molecules (https://cgenff.umaryland.edu/; Yu et al., 2012; Vanommeslaeghe et al., 2010). The ligand and protein complexes were solvated using TITP3 water model in a dodecahedral simulation box with 1 ns spacing between the complex surface and the box. The calculated charge on the protein was neutralized using the corresponding number of sodium or chloride ions by replacing the solvent molecules. The energy minimization of the system was carried out using a steepest descent algorithm followed by two 100 ps phases of equilibrium i.e. NVT (constant number of particles, volume and temperature at 300 K) and NPT (constant number of particles, pressure of 1.0 bar and temperature). The final MD simulation was carried out for 10 ns with each step of 2 fs. The Berendsen thermostat was used for controlling temperature while the LINCS (linear constraint) algorithm was used to constrain covalent bonds. The Particle Mesh Ewald Method was employed for calculating long range electrostatic interactions. The gmx_MMPBSA analysis was performed using the instructions given for Protein-ligand (https://valdes-tresanco-ms.github.io/gmx_MMPBSA/dev/examples/Protein_ligand_CHARMMff/) and Protein-ligand with LPH (lone pair: hydrogen) particles after removing the lone pairs not supported in gmx_MMPBSA algorithm (https://valdes-tresanco-ms.github.io/gmx_MMPBSA/dev/examples/Protein_ligand_LPH_atoms_CHARMMff/). The first 100 frames were discarded and binding free energy for the remaining 900 frames with the interval of 2 frames was considered for calculations.

## 3 Results

This study explored the interactions of two PfJDPs with their corresponding PfHsp70s; PfHsp70-x-PFE0055c and PfHsp70-2-PfSec63. The analyses of these malarial chaperone-co-chaperone complexes in comparison to the homologous human complexes are presented, followed by the outcomes of a virtual molecular-docking-based screen of the PRB database.

### 3.1 Sequence and structural comparison of the Hsp70 Nucleotide Binding Domains and the J domains of the JDPs

Hsp70s consist of three domains; the N-terminal NBD, the middle linker domain, and the C-terminal Substrate Binding Domain (SBD). A detailed structural analysis of the canonical Hsp70-JDP complex of *E. coli* (EcDnaK-EcDnaJ), has revealed the contact residues of three important binding sites: (i) the J domain helix II interface with the NBD; (ii) the HPD motif and J domain helix III interface with the SBD; and (iii) the J domain HPD catalytic interface with the NDB, linker region and SBD (Kityk et al., 2018). The J domain helix II interface with the NBD is a key initial binding step which is critical for the positioning of the catalytic HPD motif (Kityk et al., 2018). This prompted us to explore the NBD-J domain helix II interface as a focus to perform virtual screening studies, using readily available high resolution structures for Hsp70 NBDs and JDP J domains.

**FIGURE 1:**
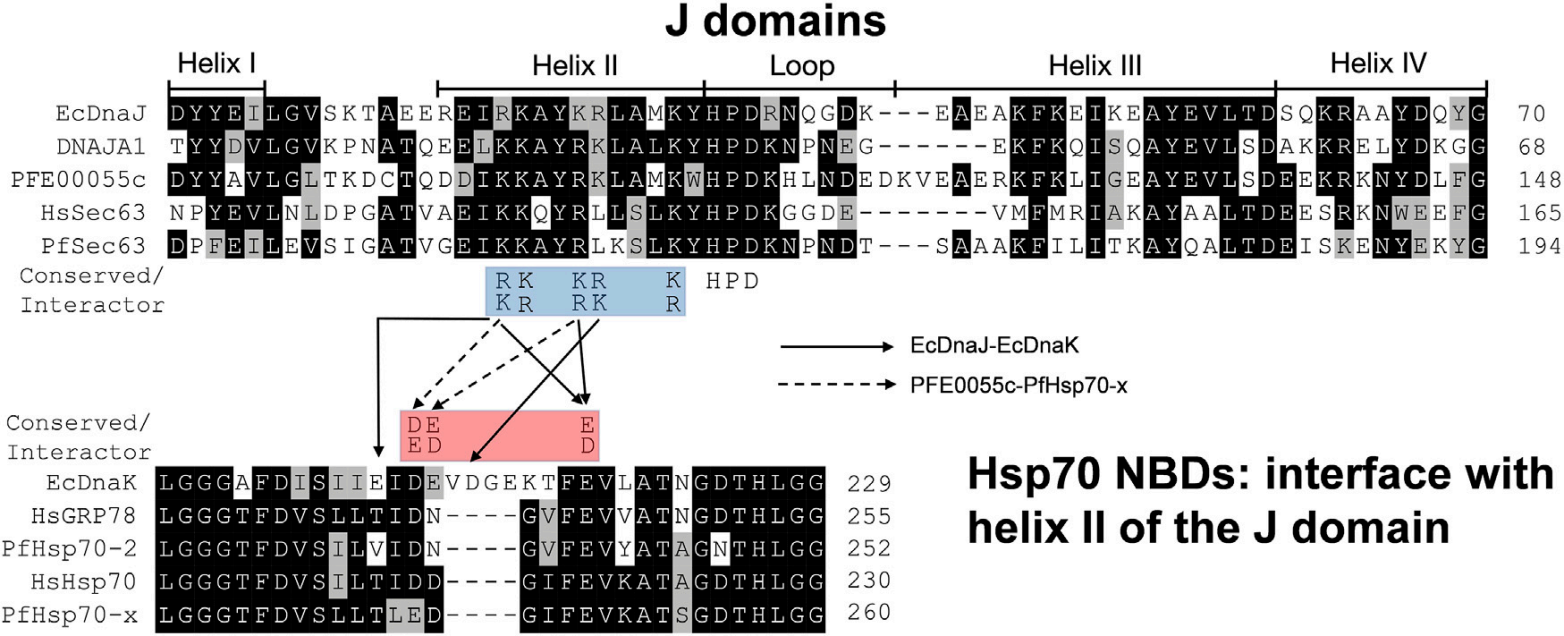
The J domain helix II interface with the NBD in JDP-Hsp70 interaction. Positively charged residues on helix II of the J domain (blue highlighted residues; R/K22, K/R23, K/R26, R/K27 and K/R31, EcDnaJ numbering) have been found to be highly conserved in JDPs and important for functional interaction and/or binding (Greene et al., 1998; Berjanskii et al., 2000; Genevaux et al., 2000; Hennessy et al., 2000; Hennessy et al., 2005; Nicoll et al., 2007; Hatherley et al., 2014; Kityk et al., 2018; Dutta et al., 2021b) to negatively charged residues (red highlighted residues; D/E208, D/E209, and D/E217, EcDnaK numbering) on the undersidecleft of the NBD of Hsp70s (Osipiuk et al., 1999; Hughes et al. 2016; Day et al., 2019). The strictly conserved catalytic HPD motif is also shown. Solid and dashed arrows refers the pairs of residues interacting specifically within the Hsp70 NBD-J domain of the JDP for EcDnaJ-EcDnaK and PFE0055c-PfHsp70-x interfaces, respectively. The proteins are defined by either their PlasmoDB accession number or common name in the first column. Colored in black are identical amino acids (in at least 50% of the aligned sequences), colored in light grey are similar amino acids (in at least 50% of the aligned sequences), and colored in white are the amino acids with no identity or similarity. The default categories for similar amino acids were applied to the multiple sequence alignment (ILV, FWY, KRH, DE, GAS, P, C and TNQM). The protein helices and loop region in the J domains are defined by bidirectional lines on top of the alignment. The alignments were created using Clustal Omega (Sievers and Higgins, 2017) and rendered with box shading using Multiple Align Show (Stothard, 2000).

**FIGURE 2:**
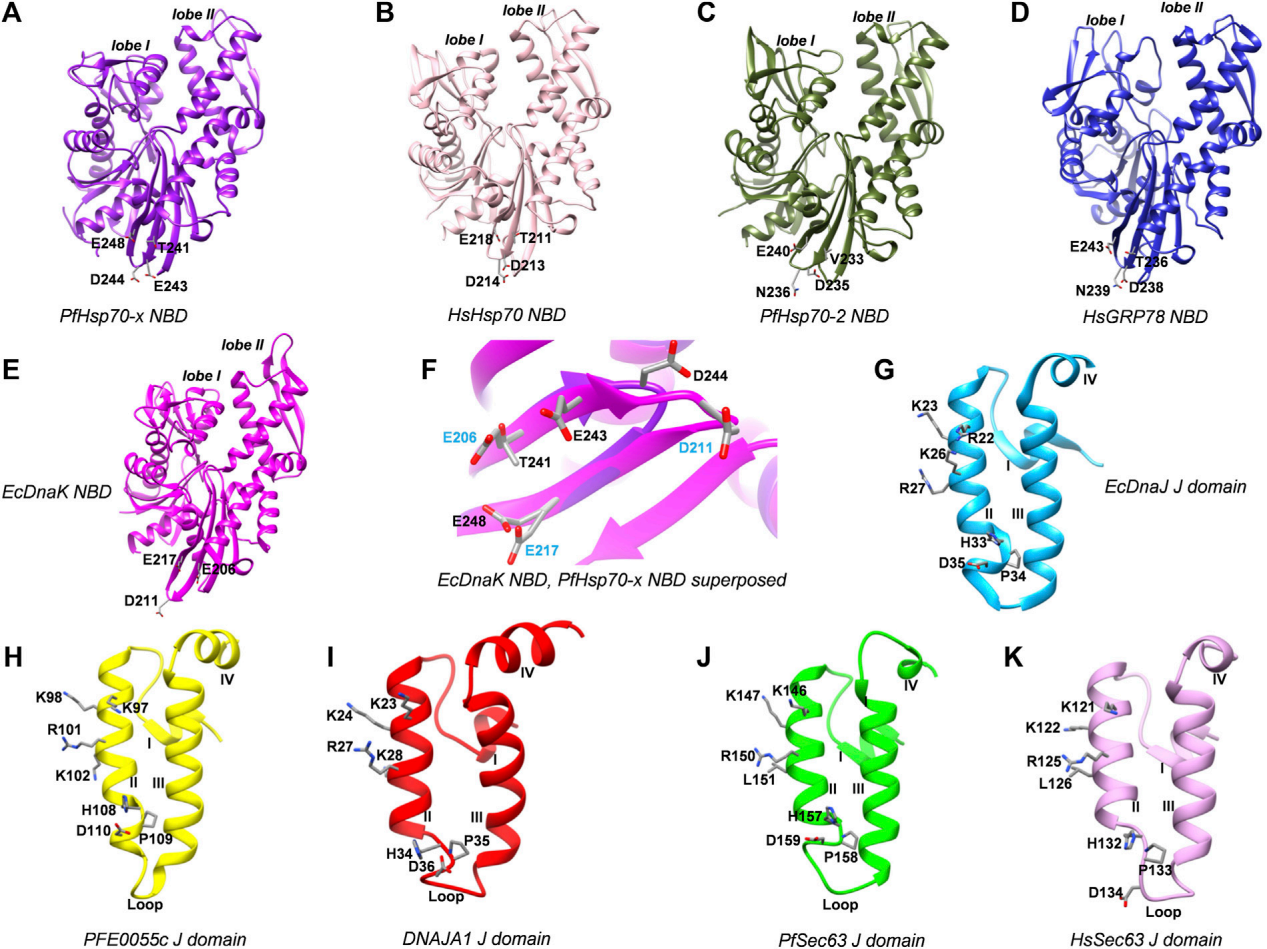
3D models of Hsp70 NBDs, and J domains of various interacting JDPs. **(A)** PfHsp70-x NBD, purple, PDB ID: 6S02 with missing residues at positions 217-221 modeled using EcDnaK as a template (PDB ID: 5NRO). **(B)** HsHsp70/HSPA1A NBD, pink, PDB ID: 1HJO. **(C)** PfHsp70-2 NBD, olive green, PDB ID: 5UMB. **(D)** HsGRP78/HSPA5 NBD, blue, PDB ID: 5F2R. **(E)** EcDnaK NBD used as a reference, magenta, PDB ID: 5NRO. **(F)** Superposition of EcDnaK NBD and PfHsp70-x NBD, focussed on key residues labelled in blue and black color, respectively. **(G)** EcDnaJ J domain used as a reference, sky blue, PDB ID: 5NRO. **(H)** PFE0055c J domain, yellow, modeled using EcDnaJ as a template (PDB ID: 5NRO). **(I)** HsJDP, DNAJA1 J domain, red, PDB ID: 2M6Y. **(J)** PfSec63 J domain, green, modeled using EcDnaJ as a template (PDB ID: 5NRO). **(K)** HsSec63 J domain, plum, modeled using mouse DnaJ as a template (PDB ID: 2CUG). Key residues are shown as sticks and are colored by element type. These residues correspond to interacting regions of the NBD interface in the experimentally determined EcDnaK-EcDnaJ complex (Kityk et al., 2018). The numbering of amino acid residues in each of the J domains corresponds to positions of amino acids in their respective full length protein sequences. The Roman numerals I, II, III and IV denote helices I to IV, respectively, in the J domains. The lobe I and lobe II represent the two lobes in the NBD of respective Hsp70 structures.

Using EcDnaK as a reference, a multiple sequence alignment was created of the region of the NBDs of PfHsp70x, Human Hsp70 (HsHsp70; HSPA1A), PfHsp70-2 and Human GRP78 (HsGRP78; HSPA5) that forms an interface with helix II of the J domain; and using EcDnaJ as a reference, a multiple sequence alignment was created of the J domains of the PFE0055c, DNAJA1, PfSec63 and HsSec63 (Figure 1). A high level of conservation was apparent throughout these domains at sequence (Figure 1; Supplementary Figure S3) as well as structural levels (Figure 2). The 3D models of NBDs and J domains further revealed the attainment of the 3D structure typical of the canonical EcDnaK NBD and EcDnaJ J domain, respectively. Analysis of the conservation of residues involved in the NBD-J domain helix II interface, identified specific residue pairs involved in JDP-Hsp70 interaction (Figure 1). The interacting residue pairs in EcDnaK-EcDnaJ (Kityk et al., 2018) and PfHsp70-x-PFE0055c (Hatherley et al., 2014; Dutta et al., 2021b) were highlighted against the multiple sequence alignment (Figure 1, solid and dashed arrows, respectively).

Out of the NBD interface residues (Figure 1; Figures 2A–E) reported to interact with helix II of the J domain *viz.*, 206 (E), 211 (D), 217 (E) in the reference NBD of EcDnaK (Kityk et al., 2018), the residues corresponding to 206 (E) were found to be the most variant (mutated to either T or V), while those residues corresponding to 217 (E) was fully conserved among all the proteins (Table 1). The residues corresponding to 211 (D) were found to be deleted as a part of a four residue long deletion, such that other residues (e.g. E243 and D244 of PfHsp70-x NBD) attain a topologically equivalent position to that of 206 (E) and 211 (D) as depicted by the 3D models (Figure 2F). However, the exact role of these residues needs to be experimentally validated.

As given in Table 1, the amino acids corresponding to the three positively charged interacting residues *viz.,* 22 (R), 26 (K), 27 (R) of the EcDnaJ J domain making the helix II interface, are conserved in the PFE0055c and human DNAJAI J domains. However, the third residue, corresponding to 27 (R) of the EcDnaJ J domain is mutated to L in both Sec63 proteins (Figure 1, Figures 2G–K). It is notable that a series of positively charged residues of the JDP J domain helix II are involved in binding or functional interaction with negatively charged residues of the NBD of Hsp70, with K/R26 in particular, being highly conserved in JDPs of prokaryotic, parasitic and mammalian systems (Genevaux et al., 2000; Hennessy et al., 2000; Hennessy et al., 2005; Nicoll et al., 2007).

### 3.2 Docking Hsp70 NBD and JDP J domains

As indicated in the previous section, the focus of this study is the J domain helix II interface with the NBD, since it is a key initial binding interface critical for Hsp70-JDP functional interaction. Hence, based on the sequence and structurally conserved positively (J domains) and negatively charged (NBD) residues making up the interface (Figures 1, 2, Supplementary Figure S3; Table 1), protein-protein docking of various Hsp70 NBDs with their corresponding partner JDP J domains was carried out using the HADDOCK2.4 server. While docking these proteins, the interface residues were marked to obtain docking conformations with corresponding interfaces facing each other. Since, PFE0055c along with other exported type II PfJDPs have been reported to interact with HsHsp70 (Jha et al., 2017), docking of PFE0055c and DNAJA1 J domains was also carried out with HsHsp70 and PfHsp70-x NBDs, respectively (Table 2). The highest Haddock score as well as the electrostatic energy of interaction was obtained for PfHsp70-x-PFE0055c (-95.4±4.3, -494.1±21.7) and PfHsp70-2-PfSec63 (-81.7±12.3, -386.1±85.7) complexes. Interestingly, the Haddock score of the HsHsp70-PFE0055c complex (-70.9±5.0) was slightly higher than that of the native HsHsp70-DNAJA1 complex (-67.6±6.0), suggesting a competitive interaction of HsHsp70 with the native DNAJA1 as well as exported PFE0055c. However, as per the preference for binding of PFE0055c, PfHsp70-x appears to be the preferred partner in comparison to HsHsp70.

**TABLE 2:** Protein-protein docking of Hsp70 NBD-JDP J domain complexes.

S. No.	Protein 1	Protein 2	Haddock score, electrostatic energy of the top cluster	RMSD in References to EcDnaK-EcDnaJ J domain (Å)
1	PfHsp70-x NBD	PFE0005c J domain	-95.4±4.3, -494.1±21.7	0.67
2	PfHsp70-x NBD	DNAJA1 J domain	-65.7±5.5, -338.9±86.0	0.527
3	HsHsp70 NBD	PFE0005c J domain	-70.9±5.0, -308.9±39.5	1.085
4	HsHsp70 NBD	DNAJA1 J domain	-67.6±6.0, -310.7±44.7	1.046
5	HsGRP78 NBD	HsSec63 J domain	-65.9±6.2, -358.5±36.9	0.827
6	PfHsp70-2 NBD	PfSec63 J domain	-81.7±12.3, -386.1±85.7	0.926

### 3.3 Virtual screening of Hsp70 NBD-JDP J domain complexes and JDP J domains against the Pandemic Response Box database

Virtual screening to identify JDP inhibitors was carried out using two strategies. The first strategy made use of the complex between the Hsp70 NBDs and corresponding JDP J domains to look for potential ligands from the PRB library which exhibited the potential to disrupt the interaction between the components of the complex. The second strategy, on the other hand, mines for ligands which show preferential binding to the JDP J domains, thereby limiting the binding of J domain to the NBD.

#### 3.3.1 Virtual screening of cytosolic Hsp70s and cytosolic/exported JDP

PFE0055c and DNAJA1 are cytosolic/exported JDPs, so the virtual screening setup was targeted at four complexes *viz.*, PfHsp70-x-PFE0055c, PfHsp70-x-DNAJA1, HsHsp70-DNAJA1 and HsHsp70-PFE0055c, to search for compounds which bind to PfHsp70-x complexes preferentially as compared to the HsHsp70 complexes. The docking was carried out using the NBD of the Hsp70 proteins and the J domain of the JDP proteins. In addition, C86 was used as a reference compound as it has been implicated in disrupting the complex formation between PfHsp70-x and PFE0055c (Dutta et al., 2021b). The binding affinity values (kcal/mol) for the PfHsp70-x-PFE0055c, PfHsp70-x-DNAJA1, HsHsp70-DNAJA1 and HsHsp70-PFE0055c complexes ranged from -3.8 to -8.5, -3.5 to -8.5, -3.7 to -9.1, and -3.6 to -9.7, respectively. The virtual screening results were sorted based on the difference in binding affinities between the PfHsp70-x-PFE0055c and the HsHsp70-DNAJA1 complexes (Supplementary Table S13). The top scoring compounds with differential binding affinities greater than or equal to -6.0 kcal/mol, for the PfHsp70-x-PFE0055c and HsHsp70-DNAJA1 have been presented in Supplementary Table S3 along with binding affinity values for individual docked poses. It is often challenging to find high affinity binding solutions for a macromolecule/macromolecule complex (Huggins et al., 2012; Ramírez and Caballero, 2018), and relying solely on the docking score may lead to a significant number of false positives. So, a visual inspection was carried out to find compounds with docking poses bound to the interface as well having differential binding affinities. The top scoring docking solutions thus obtained are presented in Supplementary Table S2 which specifies the binding affinities of the top ranked docking pose for each compound while the binding affinities of the individual docking poses are given in Supplementary Table S3. The two dimensional (2D) structures of the top scoring compounds have been presented to give a glimpse of the diversity of chemicals selected through virtual screening, as well as the prominent features they possess in common (Supplementary Figure S4, Supplementary Table S4). With the exception of **60196939**, all the identified compounds share aromatic motifs linked by a short, flexible linker to another unsaturated (typically aromatic) unit, and all possess H-bond acceptor sites making this group chemically consistent.

An overview of the different docked poses for each of the compounds has been depicted in Supplementary Figure S5. The binding poses exactly interfering at the interface region were considered on-target dockings. As PfHsp70-x-PFE0055c had strong binding affinity (Haddock score -95.4±4.3) with a major contribution from the electrostatic energy (-494.1±21.7), it may not have been possible to frequently get a conformation docked exactly into the interface region leading to low on-target docking solutions. However, PfHsp70-x-PFE0055c on-target docking poses maintained higher binding affinity compared to the best on-target poses in their human counterpart (HsHsp70-DNAJA1).

A graphical comparison of docking poses for PFHsp70-x-PFE0055c and HsHsp70-DNAJA1, selected on the basis of their binding affinity to the interface region have been presented in Supplementary Figure S6. This graphic shows the overall docking poses, binding affinities of the comparative poses in reference to C86, and their interaction maps to give a comprehensive overview of docking selectivity. The compounds **833990, 9819085** and **10046204** are known anti-viral agents whereas the compounds **60196939** and **2862146/MBX 1641** are both anti-bacterial compounds. It is notable that all these compounds possess binding affinities higher than the HsHsp70-DNAJA1 complex. The compounds **833990**, **60196939** and **2862146/MBX 1641** seem to be specific for PFE0055c while the compounds **9819085** and **10046204** preferentially bind both PfHsp70-x complexes as well as the HsHsp70-PFE0055c complex, suggesting they are potential pan-inhibitors like C86.

**FIGURE 3:**
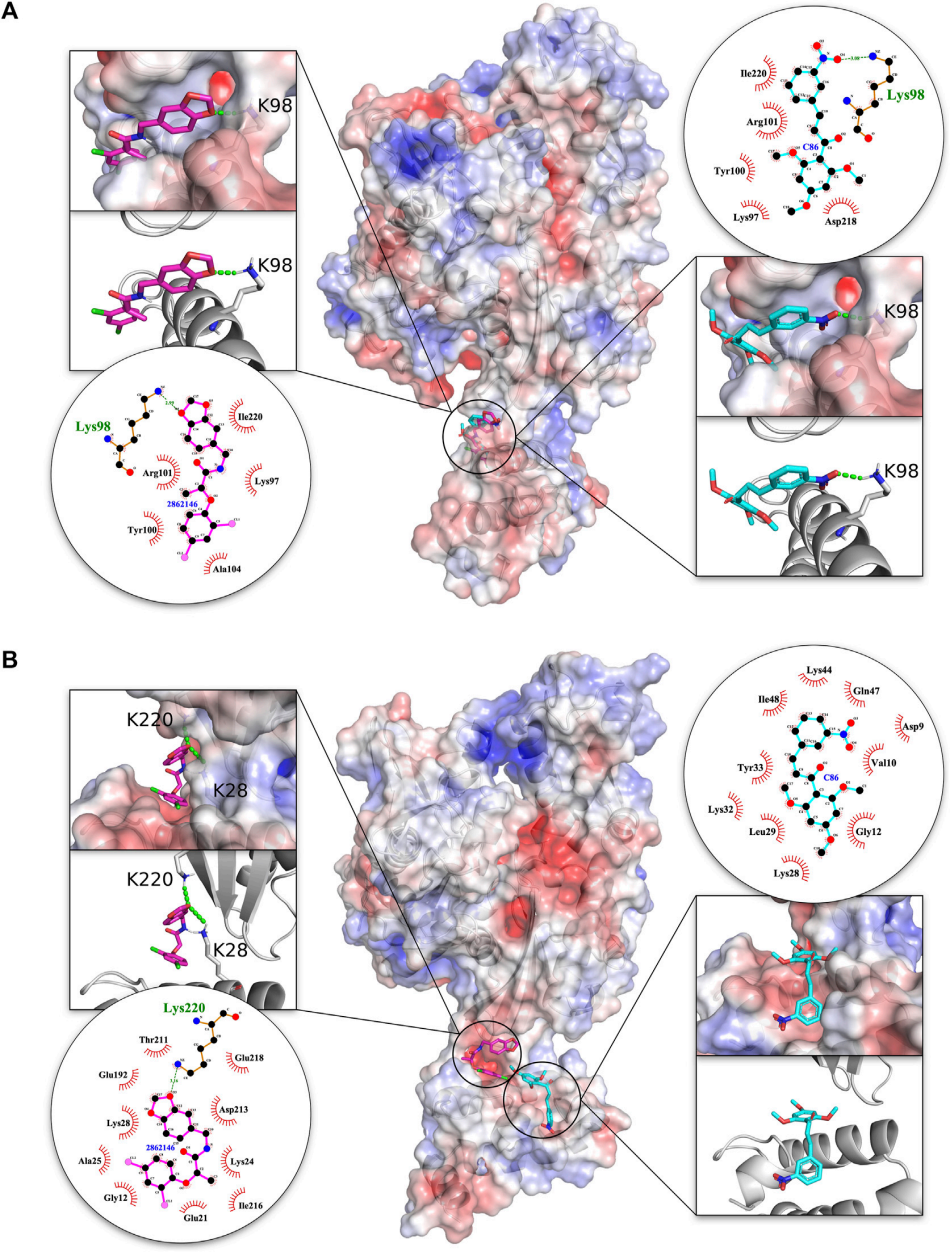
3D structures of molecular docking of **(A)** PfHsp70-x-PFE0055c and **(B)** HsHsp70-DNAJA1 in complex with **2862146/MBX 1641** and C86. The protein structures in the middle show the docked **2862146/MBX 1641** ligand as pink sticks and C86 as cyan sticks. The zoom-in on each side of the protein structure shows the docked ligands, with amino acid residues forming H-bonds (green dotted lines) with the ligand shown as white sticks with nitrogen and oxygen highlighted with blue and red colors, respectively. The positive charge is shown in blue colored surface, negative charge is shown in red colored surface and neutral potentials are shown in white colored surface. The surface electrostatic potential was calculated by APBS and graphically rendered using PyMol 2.5.2 (PyMOL Molecular Graphics System, Version 2.0 Schrödinger, LLC). The contact analysis on each side of the protein structures shows the ligand-protein interaction diagrams. The bonds within **2862146/MBX 1641** are shown with thick pink lines, bonds within C86 are shown with cyan lines, non-ligand bonds belonging to protein residues to which the ligand has H-bonds are shown with thin gold bonds, nitrogen and oxygen are highlighted with blue and red colors dots, respectively, H-bonds are shown by green dashed lines with the length of the bond printed in the middle, and hydrophobic contacts between protein and ligand are indicated by the brick-red spoked arcs. The plots were generated by LigPlot+ (Laskowski and Swindells, 2011).

An in-depth analysis of the H-bonding and hydrophobic interactions between ligand and target protein was performed for the best docked energy complexes alongside C86 that served as positive control for the malarial complex and a negative control for the human complex (Figure 3). Results revealed that, in both the malarial and human complexes, **2862146/MBX 1641** docked completely within the interface. In PfHsp70-x-PFE0055c, **2862146/MBX 1641** overlaps with the positive control C86, forming a stronger H-bond with the same residue (K98) in the J domain of PFE0055c that makes contact with C86, in addition to having similar hydrophobic/other non-covalent interactions with amino acid residues K97, Y100, R101, A104 (J domain) and I220 (NBD). Interestingly, in the HsHsp70-DNAJA1 complex, **2862146/MBX 1641** is oriented in a manner that it forms H-bonds with both proteins in the complex; forming H-bonds with K220 in HsHsp70, and with K28 in DNAJA1 (which is the topologically equivalent to PFE0055c K98). It seems that **2862146/MBX 1641** is acting as molecular glue binding together HsHsp70 and DNAJA1. In addition, **2862146/MBX 1641** is predicted to form hydrophobic/other non-covalent contacts with residues G12, E21, A25, K24, K28 of the J domain and E192, T211, D213, I216, E218 and K220 of the NBD. C86, seems to bind the HsHsp70 NBD only via hydrophobic interactions/other non-covalent interactions. This analysis indicates that **2862146/MBX 1641** has a binding preference to the J domain residues in the PfHsp70-x-PFE0055c complex while it maintains contacts with both the partners in the HsHsp70-DNAJA1 complex having a competitive inhibitory potential in the malarial system and stabilizing effect in the human system.

#### 3.3.2 Virtual screening of ER specific Hsp70s and JDPs

Virtual screening was also conducted on the Hsp70 and J domain complexes specifically expressed in the ER, and which have recently been proposed as potent drug targets against malaria (Chen et al., 2018; Mrozek et al., 2022). Virtual screening of the PfHsp70-2-PfSec63 and HsGRP78-HsSec63 complexes was carried out using the PRB compound library. Due to significant conservation in the overall NDB as well J domains of these ER specific complexes, the same interface region was defined as in the cytosolic/exported complexes discussed in the previous section to explore potential inhibitors. The binding affinity of the docking solutions ranged from -4.0 to -9.0 kcal/mol for PfHsp70-2-PfSec63 and from -3.6 to -8.8 kcal/mol for HsGRP78-HsSec63. The docking solutions were sorted based on the difference in binding affinities between the PfHsp70-2-PfSec63 and the HsGRP78-PfSec63 complexes (Supplementary Table S14), to obtain top scoring compounds with differential binding affinities of PfHsp70-2-PfSec63 over HsGRP78-PfSec63 greater than or equal to -0.7 kcal/mol, overall binding affinities higher than or equal to -7.0 kcal/mol and for whom the docking interface overlapped with the reference, C86 (Supplementary Table S5). The compounds **10955174** and **135398740** were not considered for further analysis as all the binding poses were located outside of the interface region. In addition, the binding affinity values for individual docked poses for all compounds screened was also compiled (Supplementary Table S6). An overview of the chemical space of the identified compounds is presented in Supplementary Figure S7 and Supplementary Table S7, whereas Supplementary Figure S8 gives an overview of the different docked poses for each of the compounds. Unlike the structures that bind to cytosolic complexes as provided in Supplementary Figure S4, those that bind to ER complexes are structurally much more variable. Motifs include a β-lactam, polycyclic aromatics, spiro-centers, a pseudo-peptide *etc.* That said, many contain imidazole or azole rings, and given the isomeric nature of the latter with triazole, which is easily accessible via Cu-catalyzed azide “click chemistry”, many of the identified compounds could be amenable to analysis using pull-down assays with suitable linkers.

The majority of the dockings were specific to the interface region in contrast to the trend observed for the docking screen of PfHsp70-x-PFE0055c (compare Supplementary Figure S5 with Supplementary Figure S8). This may be attributed to a weaker interaction between the PfHsp70-2 and PfSec63 (Haddock score: 81.7±12.3, electrostatic energy: 386.1±85.7), which might result in more space available at the interface, and therefore greater access for the ligands. In addition, the comparison of docking poses for PfHsp70-2-PfSec63 and HsGRP78-HsSec63, selected on the basis of their binding affinity to the interface region have been presented in Supplementary Figures S9 and S10. These figures summarize the overall docking poses and binding affinities of the comparative poses in relation to C86, whilst also providing a graphical depiction of the interacting residues at the interface. Out of these top scoring compounds, four (**60196939**, **6098**, **57383474**, and **76685216/zoliflodacin**) are anti-bacterial, four (**10324367**, **5189681**, **45138674**, and **11485687**) are anti-viral and three (**137648729**, **467825**, and **55283/itraconazole**) are anti-fungal (Supplementary Table S5). All the compounds in this table possess higher binding affinity for PfHsp70-2-PfSec63 as compared to HsGRP78-HsSec63, with compound **76685216/zoliflodacin** possessing the highest (-9.0 kcal/mol) followed by **55283/itraconazole** (-8.3 kcal/mol). However, compound **60196939** (-1.7 kcal/mol) had the largest difference in binding affinity followed by the compound **6098** (-1.5 kcal/mol).

**FIGURE 4:**
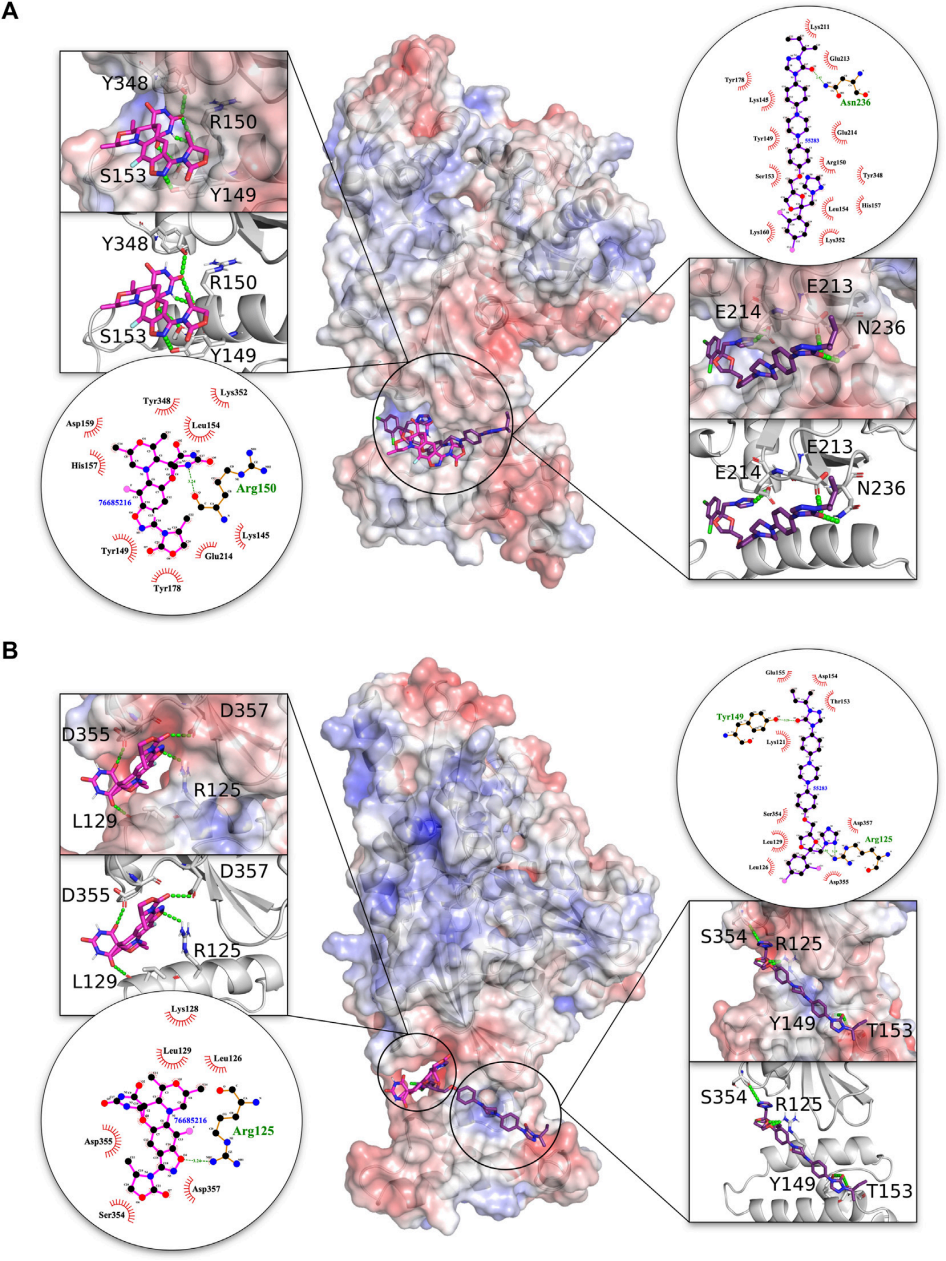
3D structures of molecular docking of **(A)** PfHsp70-2-PfSec63 and **(B)** HsGRP78-HsSec63 in complex with **76685216/zoliflodacin** and **55283/itraconazole**. The protein structures in the middle show the docked **76685216/zoliflodacin** ligand as pink sticks and **55283/itraconazole** as purple sticks. The zoom-in on each side of the protein structure shows the docked ligands, with amino acid residues forming H-bonds (green dotted lines) with the ligand shown as white sticks with nitrogen and oxygen highlighted with blue and red colors, respectively. The positive charge is shown in blue colored surface, negative charge is shown in red colored surface and neutral potentials are shown in white colored surface. The surface electrostatic potential was calculated by APBS and graphically rendered using PyMol 2.5.2 (PyMOL Molecular Graphics System, Version 2.0 Schrödinger, LLC). The contact analysis on each side of the protein structures shows the ligand-protein interaction diagrams. The bonds within **76685216/zoliflodacin** are shown with thick pink lines, and the bonds within **55283/itraconazole** are shown with thick purple lines, non-ligand bonds belonging to protein residues to which the ligand is H-bonded are shown with thin gold bonds, nitrogen and oxygen are highlighted with blue and red color dots, respectively, H-bonds are shown by green dashed lines with the length of the bond printed in the middle, and hydrophobic contacts between protein and ligand are indicated by the brick-red spoked arcs. The plots were generated by LigPlot+ (Laskowski and Swindells, 2011).

On the basis of the visual inspection of docked poses in the interface region, compounds **76685216/zoliflodacin** and **55283/itraconazole** were found to be the best-docked solutions as depicted in Figure 4. These compounds docked completely within the interface in both the malarial and human complexes, with **76685216/zoliflodacin** forming four H-bonds across both proteins in each complex. The modulator **76685216/zoliflodacin** forms four H-bonds with residues Y149, R150 (equivalent to R97 of PFE0055c), S153 of the J domain and Y348 of the NBD in PfHsp70-2-PfSec63 complex while maintaining hydrophobic/other non-covalent interactions with residues K145, L153, H157, D159, Y178 of the J domain and residues E214, K352 of the NBD. The interaction of the HsGRP78-HsSec63 complex with **76685216/zoliflodacin** also involved four H-bonds with residues R125 (equivalent to R97 of PFE0055c), L129 of the J domain and D355, D357 of the NBD apart from making hydrophobic/other non-covalent interactions with residues L126, K129 (J domain) and S354 (NBD).

The compound **55283/itraconazole** forms four H-bonds with both proteins in the HsGRP78-HsSec63 complex, but only forms three H-bonds with PfHsp70-2 in the PfHsp70-2-PfSec63 complex. However, the H-bonds with the PfHsp70-2-PfSec63 complex involve residues E213, E214 and N236 of the NBD, with the former two being reported to be critical for NBD-J domain complex formation in the malarial system (Dutta et al., 2021b). On the other hand, in the HsGRP78-HsSec63 complex **55283/itraconazole** was predicted to form H-bonds with residues R125, Y149 and T153 of J domain and S354 of the NBD. The overall pose and H-bond pattern of **76685216/zoliflodacin** and 55283**/itraconazole** involving critical residues involved in NBD and J domain interaction as well their positioning along helix II of the J domain, indicate their inhibitory potential for the malarial system, compared to their stabilizing effect on HsGRP78-HsSec63, involving R125 (equivalent to R97 of PFE0055c) and residues from NBD regions other than the reported interface. Overall, the data suggested that these modulators potentially inhibit the malarial system, while stabilizing or minimally inhibiting the human system.

#### 3.3.3 Virtual screening based on J domains

Previous studies by our group reported that the pre-incubation of PFE0055c with C86 prior to the addition of PfHsp70-x in the reaction mixture, resulted in significant inhibition of the PFE0055c-stimulated ATPase activity of PfHsp70-x compared to the assay without pre-incubation (Dutta et al., 2021b). Based on this observation, we performed a virtual screening to select potential binders to the helix II region of the J domain, specifically harboring residues corresponding to the EcDnaJ helix II residues *viz.*, 22 (R), 26 (K), 27 (R) (Kityk et al., 2018). The 3D structures of J domains of two cytosolic/exported (PFE0055c, DNAJA1) and two ER JDPs (PfSec63, HsSec63) were used to perform virtual screening of the PRB compound library. The binding affinity of the docking solutions ranged from -3.4 to -9.0, -3.5 to -8.5, -3.6 to -8.0, and -3.5 to -7.5 for PFE0055c, DNAJA1, PfSec63, and HsSec63, respectively.

The docking solutions were sorted based on the difference in binding affinities between PFE0055c and DNAJA1 (Supplementary Table S15). The sorted table highlighted the top scoring compounds with binding affinity difference greater than or equal to -1.2 kcal/mol, for PFE0055c and PfSec63 over their counterparts in the human system (i.e. DNAJA1 and HsSec63), respectively (Supplementary Table S8). In addition, it was observed that a large number of compounds bound preferentially to PFE0055c (Supplementary Table S8). The reference compound, C86 was also observed to bind with greater affinity to the PFE0055c J domain (-6.0 kcal/mol) than the other three J domains (-5.3 kcal/mol). The structures of the top binding compounds specific to both PFE0055c and PfSec63 have been depicted in Supplementary Figure S11 and Supplementary Table S11, to provide an overview of their chemical nature. The overlay of the individual docking poses onto the PFE0055c J domain (Supplementary Figure S12) revealed specificity for the helix II region of the PFE0055c J domain, similar to C86, indicating their potential to interfere with PfHsp70-x-PFE0055c complex formation. The binding affinity values for individual docked poses for these compounds also depicted the same trend as the top ranked poses (Supplementary Table S9, S10).

All of the top scoring compounds (**150311/ezetimibe**, **2867190/benzo-diazepinone**), **10077130/vorapaxar**, **1317590**, **9911469**/FK-788) specific for PfJDPs are anti-viral in nature. Out of the PFE0055c J domain specific binders, the majority of them are anti-viral (**1684/pleconaril**), **2768133**, **3783668**, **51354502**, **55245/mifepristone**, **16739062**, **56668933**) followed by anti-bacterial (**8392433**, **76310291**, **115358/tafenoquine**, **71450388**, **1158563**, **2763159**) and anti-fungal (**155546288**, **10027278**, **155541425**) in reported bioactivity. The comparative graphics of all the PfJDPs specific inhibitors depicts their overall interaction maps with different J domains (Supplementary Figure S13, S14, S15, S16). These figures indicate the stronger and more specific binding to the PFE0055c J domain compared to all the J domains, while also indicating the stronger binding affinity score for the PfSec63 J domain in comparison to HsSec63 J domain. The highest differential binding affinity for the unbound J domains was -1.9 kcal/mol as compared to the scenarios when the compounds were targeted against the J domains bound to their corresponding Hsp70s (-0.9 kcal/mol for PfHsp70-x-PFE0055c and HsHsp70-DNAJA1; -1.7 kcal/mol for PfHsp70-2-PfSec63 and HsGRP78-HsSec63). More specific binding to the PFE0055c J domain with large differential binding affinities in comparison to the human counterparts may be attributed to the greater conformational space that the unbound J domain affords compared to the bound J domain. The J domains of PfSec63 as well as HsSec63 showed an overall binding preference towards the ends of the helix II region, specifically towards the HPD motif, as compared to PFE0055c and DNAJA1 J domains. One of the reasons for such a behaviour may be the mutation of one of the three positively charged residues (*viz.,* 22 (R), 26 (K), 27 (R) of the EcDnaJ J domain), i.e. 27 (R) to L in PfSec63 and HsSec63 J domain (Figures 1, 2). This would subtly change the electrostatic surface potential of helix II, shifting the optimal binding region towards the HPD end of helix II.

**FIGURE 5:**
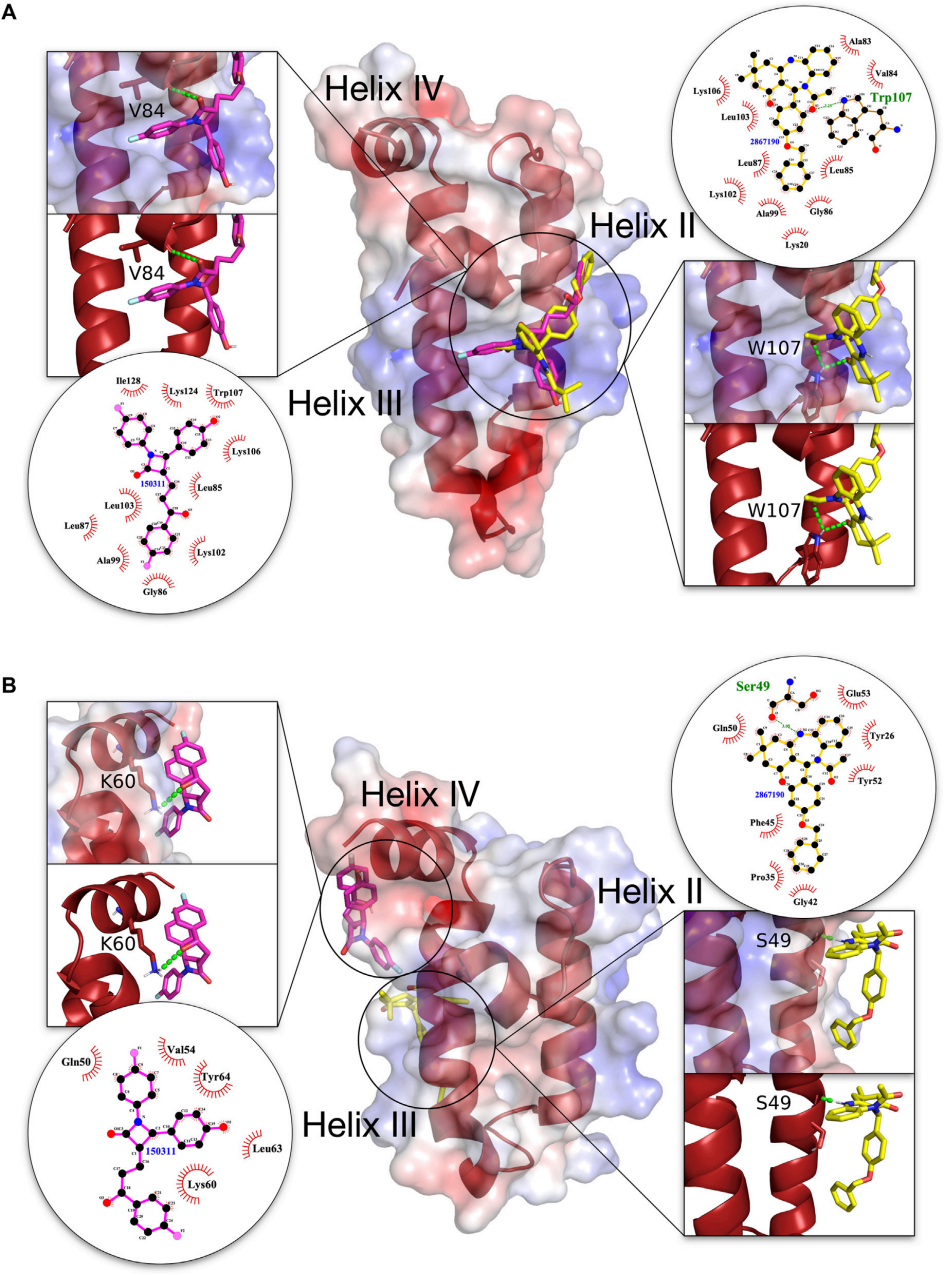
3D structures of molecular docking of **(A)** PFE0055c J domain and **(B)** DNAJA1 J domain with **150311/ezetimibe** and **2867190/benzo-diazepinone**. The protein structures in the middle show the docked **150311/ezetimibe** ligand as pink sticks and **2867190/benzo-diazepinone** as yellow sticks. The zoom-in on each side of the protein structure shows the docked ligands, amino acid residues forming H-bonds (green dotted lines) with the ligand as dark red sticks, and nitrogen and oxygen highlighted with blue and red colors. The positive charge is shown in blue colored surface, negative charge is shown in red colored surface and neutral potentials are shown in white colored surface. The surface electrostatic potential was calculated by APBS and graphically rendered using PyMol 2.5.2 (PyMOL Molecular Graphics System, Version 2.0 Schrödinger, LLC). The contact analysis on each side of the protein structures shows the ligand-protein interaction diagrams. The bonds within **150311/ezetimibe** are shown with thick pink lines, the bonds within **2867190/benzo-diazepinone** are shown with thick yellow lines, non-ligand bonds belonging to protein residues to which the ligand is H-bonded are shown with thin gold bonds, nitrogen and oxygen are highlighted with blue and red colors, respectively, H-bonds are shown by green dashed lines with the length of the bond printed in the middle and hydrophobic contacts between protein and ligand are indicated by the brick-red spoked arcs. The plots were generated by LigPlot+ (Laskowski and Swindells, 2011).

Detailed graphical depiction of interacting residues were created for the first two top scoring compounds (**150311/ezetimibe**, and **2867190/benzo-diazepinone**) with the highest binding energy difference between malarial and human J domains, to highlight specific contact residues and H-bond formation (Figure 5). The ligand-protein interaction as shown in this figure indicated that in both PFE0055c and DNAJA1, compounds **150311/ezetimibe** and **2867190/benzo-diazepinone** predominantly form hydrophobic contacts with the proteins, with only a few H-bonds. Interestingly, for PFE0055c, the majority of the hydrophobic contacts made by both compounds are with helix II, while for DNAJA1, the majority of hydrophobic contacts made by both compounds are with helix III and IV. Compound **150311/ezetimibe** formed H-bonds with V84 of helix I in PFE0055c and K60 of helix IV in the human DNAJA1, while **2867190/benzo-diazepinone** formed H-bonds with W107 of helix II in PFE0055c and S49 of helix III in human DNAJA1. For PFE0055c, the compound **150311/ezetimibe** formed hydrophobic contacts with helix II residues (A99, K102, L103, K 106) in addition to making some contacts with helix I residues (L85, L87) and helix-III residues (L124, I128) while the compound **2867190/benzo-diazepinone** formed hydrophobic contacts with helix-II residues (A99, K102, L103, K 106) in addition to making some contacts with helix I residues (A83, V84, L85, G86, L87). However, the presence of helix II residues at the interface (Supplementary Figure, S13, S14, S15, S16) of these compounds with the J domains of the JDPs, specifically PFE0055c, indicates their potential to act as novel inhibitors.

#### 3.3.4 MD simulation validation of top ranked compounds

MD simulations of the five top ranked compounds were analyzed for conformational stability as well as the binding free energy (MMPBSA) of both Hsp70-JDP and J domains complexes. The conformational stability of the protein-ligand complexes was initially monitored in terms of the variation in the RMSDs over the simulation period (Supplementary Figure S17; Table 3).

**TABLE 3:** An overview of the binding energy obtained from MD simulation (MMPBSA affinity) of the top ranked compounds in comparison to docking affinity.

Hsp70-J domain complex			J Domain		
Name of the complex and the drug	Docking binding energy (kcal/mol)	MMPBSA binding energy (kcal/mol)	Name of the J domain and the drug	Docking binding energy (kcal/mol)	MM-PBSA binding energy (kcal/mol)
PfHsp70-x-PFE0055c-MBX 1641	-6.6	-12.73	PFE0055c J Domain- Ezetimibe	-7.6	-12.68
HsHsp70-DNAJA1- MBX 1641	-6.0	-17.17	DNAJA1 J Domain- Ezetimibe	-5.7	-11.68
PfHsp70-x-PFE0055c- C86	-5.5	-9.48	PFE0055c J Domain- Benzo-diazepinone	-8.4	-15.03
PfHsp70-2-PfSec63-Itraconazole	-8.3	-21.17	DNAJA1 J Domain- Benzo-diazepinone	-6.6	-14.85
HsGRP78-HsSec63-Itraconazole	-7.5	-16.9	PFE0055c J Domain- C86	-6.0	-11.26
PfHsp70-2-PfSec63-Zoliflodacin	-9.0	-16.48	**-**	-	-
HsGRP78-HsSec63-Zoliflodacin	-8.1	-5.08	-	-	-
PfHsp70-2-PfSec63-C86	-5.9	-19.43	-	-	-

For PfHsp70-x-PFE0055c-MBX 1641, MBX 1641 remains bound to the complex after the initial stabilizing period of about 4.5 ns with few incidents of dissociation of the J domain-MBX 1641 complex from PfHsp70-x, resulting in the MMPBSA Binding energy of -12.3 kcal/mol, while for HsHsp70-DNAJA1-MBX 1641, MBX 1641 maintained its bound conformation to the complex throughout the 10 ns simulation, resulting in the MMPBSA Binding energy of -17.17 kcal/mol (Supplementary Figure S17A; Table 3). Both of these complexes of the exported proteins with MBX 1641 seemed to be more stable as compared to the control PfHsp70-x-PFE0055c-C86 (MMPBSA Binding energy of -9.48 kcal/mol). However, in contrast to the molecular docking results, the HsHsp70-DNAJA1-MBX 1641 complex seemed to be more stable as compared to the PfHsp70-x-PFE0055c-MBX 1641 complex. It is notable that the MBX 1641 contains two lone pairs, which were removed while carrying out the MMPBSA analysis as per the requirement of the gmx_MMPBSA software. The lack of consideration of lone pairs might be the reason for overall lower binding free energy for PfHsp70-x-PFE0055c-MBX 1641 complex as compared to the HsHsp70-DNAJA1-MBX 1641 complex (given the striking difference in their electrostatic component, Supplementary Table S16, indicating the important role that lone pairs might be playing in the interaction in the former complex). The lone pair parameter will need to be explored in future studies on all of the top candidate compounds.

The PfHsp70-2-PfSec63-itraconazole and PfHsp70-2-PfSec63-zoliflodacin complexes maintained their overall conformation and binding pose stability giving the MMPBSA Binding energy values of -21.17 and -16.48 kcal/mol, respectively, as compared to their human counterpart complexes, HsGRP78-HsSec63-itraconazole and HsGRP78-HsSec63-zoliflodacin, with MMPBSA Binding energy values of -16.9 and -5.08 kcal/mol, respectively (Supplementary Figure S17B; Table 3). It is notable that the PfHsp70-2-PfSec63-itraconazole complex scored better for MMPBSA Binding energy value (-21.17 kcal/mol) as compared to both the HsGRP78-HsSec63-itraconazole (-16.9 kcal/mol) and the control PfHsp70-2-PfSec63-C86 (-19.43 kcal/mol) complexes, indicating that itraconazole formed a more stable complex in the malarial system. On the other hand, zoliflodacin seemed to form a much more stable interaction with the PfHsp70-2-PfSec63 complex (-16.48 kcal/mol) as compared to the HsGRP78-HsSec63 complex (-5.08 kcal/mol), but not as stable as the PfHsp70-2-PfSec63-C86 complex. While the interaction of itraconazole with the HsGRP78-HsSec63 complex yielded reasonable binding free energy, the binding pose showed frequent variations during different phases of the MD simulation. Also, the HsGRP78-HsSec63-zoliflodacin complex was found to be the most unstable, with very frequent fluctuations in the binding pose as well as a much lower binding free energy predicted in the MMPBSA analysis. One of the most common features of the various Hsp70-JDP complexes showing large fluctuations in the binding pose, was the separation of the J domain from the Hsp70 partner while maintaining the interaction between the ligand and the J domain. For example, zoliflodacin seemed to be disrupting the Hsp70-JDP interaction while remaining strongly bound to the J domain (Supplementary Figure S17B; Table 3). In these complexes, there seem to be some competition among the selected drug candidates and the J domains to bind to the Hsp70 partners, which needs to be validated through more robust MD simulation protocols and experimental methods.

For the MD simulation of drug candidates bound to the J domains, the PFE0055c J domain-ezetimibe complex maintained the binding pose conformation after the initial 2ns of simulation with MMPBSA Binding energy value of -12.68 kcal/mol, performing slightly better than the DNAJA1 J domain-ezetimibe (-11.68 kcal/mol) and PFE0055c J domain-C86 control (-11.26 kcal/mol). Both PFE0055c J domain-benzo-diazepinone and DNAJA1 J domain-benzo-diazepinone complexes resulted in higher MMPBSA Binding energy values of -15.03 and -14.85 kcal/mol, respectively, as compared to the control PFE0055c J domain-C86 complex (-11.26 kcal/mol) (Supplementary Figure S17C; Table 3). Although the binding of benzo-diazepinone to both the PFE0055c J domain and DNAJA1 J domain proteins seem to be similar in terms of MMPBSA Binding energy values, its binding pose with the former is more stable over the simulation period with less deviations as compared to the DNAJA1 J domain. All the J domain-ligand complexes were fairly stable during the MD simulation time period, with PFE0055c J Domain-benzo-diazepinone and DNAJA1 J Domain-benzo-diazepinone complexes showing some significant fluctuations towards the end and start of the MD simulation period, respectively (Supplementary Figure S17C). The visual inspection of the trajectories indicated that these conformational changes tend to maintain the interactions of benzo-diazepinone with the helix-II of the J domains. One reason for such variations may be the free access to the J domain structure and thereby giving more conformational flexibility to the ligands to bind to the key J domain regions, particularly the helix-II region.

## 4 Discussion

There have been extensive studies on small molecule compounds that disrupt Hsp70-JDP interaction in bacterial, yeast and human systems (Fewell et al., 2004; Wisén et al., 2010; Moses et al., 2018; Alalem et al., 2022). However, there have been limited studies on modulators of PfHsp70-PfJDP interaction (Botha et al., 2011; Cockburn et al., 2014; Daniyan and Blatch, 2017; Dutta et al., 2021b; Almaazmi et al., 2022; Almaazmi et al., 2023). The chalcone C86, previously shown to bind to the J domain and act as a pan-inhibitor of JDPs (Moses et al., 2018), was shown by us to inhibit the PFE0055c-stimulated ATPase activity of PfHsp70-x (Dutta et al., 2021b). To our knowledge this is the only evidence of a small molecule inhibitor of a J domain-based functional interaction of a PfJDP with PfHsp70. Here these previous studies have been extended by using molecular docking-based screening to identify small molecule compounds that are potentially more effective than C86 as modulators of PfHsp70-PfJDP interaction.

The use of molecular docking for virtual screening of drug repurposing libraries have been found to be very useful in discovering new anti-malarial drugs. Such strategies are more practical and less expensive than traditional approaches, while the use of drug repurposing libraries of compounds already approved for human use, rules out the need to perform expensive human toxicology studies (Li et al., 2022). The availability of a variety of drug-like small molecule compounds lodged within the drug-repurposing PRB library (Samby et al., 2022), provided an opportunity to perform virtual screening of this library to identify potential compounds targeting the NBD-J domain interface. Virtual screening was carried out using two strategies. The first strategy made use of Hsp70-JDP complexes of malarial origin (PfHsp70-x-PFE0055c and PfHsp70-2-PfSec63) compared to control homologous human complexes, to identify small molecule modulators which potentially disrupted the interaction between the components of the complex. The second strategy mined for small molecules which showed preferential binding to the malarial JDP J domains (PFE0055c and PfSec63 J domains) over the homologous human J domains, thereby identifying potential specific inhibitors of PfHsp70-PfJDP interaction.

Both of the strategies resulted in identification of a variety of compounds from the PRB library which represent potential modulators interfering with the formation of Hsp70-JDP complexes. Interestingly, Hsp70-JDP complex-based screening identified entirely different sets of compounds compared to unbound J domain-based screening. Furthermore, different sets of compounds were identified when screening for compounds with affinity and specificity for PfHsp70-x-PFE0055c as compared to PfHsp70-2-PfSec63 (with the exception of one common compound, **60196939**). These compounds possess a diverse chemical space and were originally discovered as potent anti-bacterial, anti-viral and anti-fungal agents (Supplementary Table S2, S5, S8) (Samby et al., 2022).

Out of these compounds, the five top ranked compounds possessing differential binding affinity while maintaining the optimal docking pose were selected for further validation (three top Hsp70-JDP binders, **2862146/MBX 1641**, **76685216/zoliflodacin** and **55283/itraconazole;** and two top J domain binders, **150311/ezetimibe** and **2867190/benzo-diazepinone**; Supplementary Table S12; Table 3; and Table 4). These top 5 compounds were subjected to additional three runs of re-docking using AutoDock Vina employing the same parameters as for the overall virtual screening strategy. The analysis of re-docked conformations into the NDB-J domain interface region also depicted similar interactions with similar binding affinity values indicating the robustness of the docking procedure. Furthermore, these compounds were validated using MD simulation, resulting in all the compounds, except for MBX 1461, being confirmed to bind preferentially to the malarial chaperone system over the homologous human system.

**TABLE 4:** Properties of the top ranked compounds with both differential binding affinity and optimal docking pose.

Pubchem compound ID (CID)	Common name	Hsp70-JDP docking	PRB disease indication	Anti-malarial	Inhibition activity	References
**2862146**	MBX 1641	PfHsp70-x-PFE0055c	Anti-bacterial	?	Type III secretion system inhibition	Aburto-Rodríguez et al. (2021)
**55283**	Itraconazole	PfHsp70-2-PfSec63	Anti-fungal	Yes	Cytochrome P450 3A4 inhibitor	Li et al. (2022), Penna-Coutinho et al. (2011)
**76685216**	Zoliflodacin	PfHsp70-2-PfSec63	Anti-bacterial	?	DNA gyrase inhibitor	Govender et al. (2022)
**150311**	Ezetimibe	PFE0055c J Domain	Anti-viral	Yes	Cholesterol transport inhibitor; Hsp90 Inhibitor	Hayakawa et al. (2021), Shadrack et al. (2020)
**2867190**	Benzo-diazepinone	PFE0055c J Domain	Anti-viral	?	RNA-dependent RNA-polymerase inhibitor	Singh et al. (2010), Nyanguile et al. (2008)

The overall approach to screening with the Hsp70-JDP complexes focused on identifying compounds that bound at the interface along helix II of the J domain similar to the reference C86. Interestingly, a detailed contact analysis of the top three Hsp70-JDP binders identified compounds that: 1) bound primarily to the J domain (which are potential inhibitors; PfHsp70-x-PFE0055c-MBX 1641); or 2) equally well to the J domain and NBD (which are potential stabilizers/molecular glue; zoliflodacin and itraconazole bound to both PfHsp70-2-PfSec63 and HsGRP78-HsSec63). Hence two different types of small molecule modulators have been identified, those that could disrupt the complex by inhibiting formation, and those that could disrupt the function of the complex by making it too stable or less dynamic. Indeed, given that both zoliflodacin and itraconazole were validated as having differential binding affinity to the malarial chaperone complex over the human chaperone complex, suggests that they are potential novel modulators specific to the malarial system. A detailed contact analysis of the top two J domain binders (ezetimibe and the benzo-diazepinone), revealed that they not only bound with greater apparent affinity to the J domain of PFE0055c compared to that of DNAJA1, but also adopted the preferred pose, primarily making contact with helix II on the J domain of PFE0055c while binding to other regions of the J domain of DNAJA1. These data suggest that these compounds are potential specific inhibitors of the malarial chaperone system over the human system.

The compounds **2862146/MBX 1641** and **76685216/zoliflodacin** are anti-bacterial while **150311/ezetimibe** and **2867190/benzo-diazepinone** are anti-viral in nature, and **55283/itraconazole** is an anti-fungal agent. These compounds have been reported to inhibit a variety of biological processes (Table 4) including inhibition of the Type III secretion system (MBX 1641) (Aburto-Rodrígues et al., 2021), Cytochrome P450 3A4 (itraconazole) (Penna-Coutinho et al., 2011; Li et al., 2022), DNA gyrase (zoliflodacin) (Govender et al., 2022), cholesterol transport and Hsp90 (ezetimibe) (Shadrack et al., 2020; Hayakawa et al., 2021), and RNA-dependent RNA-polymerase (the benzo-diazepinone) (Nyanguile et al., 2008; Singh et al., 2010). Notably, itraconazole (Penna-Couthino et al., 2011) and ezetimibe (Shadrack et al., 2020) possess anti-malarial activity while the other three are still to be explored for this activity. As expected, pharmacokinetic evaluation confirmed the drug-like properties of these top binder compounds (SwissADME; Daina et al., 2017). While these compounds have poor to moderate water solubility, almost all are predicted to have high GI absorption, with the ability to cross the blood-brain barrier (BBB) and interact with one or more isoenzymes of the Cytochrome P family, demonstrating their potential to be effective drugs with minimal cytotoxicity (Supplementary Table S17). Zoliflodacin was the only best binder compound that was contrary to expectations. Furthermore, all of these compounds are at different stages of clinical testing, with two already approved (itraconazole and ezetimibe).

Itraconazole is a FDA-approved drug of the triazole class known for more than 30 years for its clinically proven anti-fungal activity (Piérard et al., 2000), and recently repurposed for the treatment of cancer (Li et al., 2022). Itraconazole was among the three top scoring compounds (with atorvastatin and posaconazole) identified using molecular docking studies to potentially inhibit *P. falciparum* lactate dehydrogenase enzyme (PfLDH), a target to develop anti-malarial drugs (Penna-Coutinho et al., 2011). In addition, this study revealed that all three compounds had anti-malarial activity using *in vitro* growth assays. However, itraconazole was also found to be partially active against *P. berghei* malaria in mice (Penna-Coutinho et al., 2011). Ezetimibe binds to Niemann Pick C1 like 1 (NPC1L1), and blocks cholesterol absorption (van Heek et al., 2001), thereby lowering the levels of essential lipids, and ultimately leading to suppression of intra-erythrocytic stages of *P. falciparum* growth (Hayakawa et al., 2021). Ezetimibe has also recently been re-purposed to treat cancer by targeting Hsp90 (Shadrack et al., 2020).

This is the first time that the PfHsp70 NBD-PfJDP-J domain interface has been explored as a drug target in virtual screening against a library of small molecule compounds. The virtual screening strategies outlined in this study have identified a number of potential modulators specific for PfHsp70-PfJDPs complexes involved in survival and pathogenicity of the malarial parasite. Since these compounds were deposited in the PRB because of their drug-like properties, they could potentially be readily developed into novel anti-malarial drugs. Furthermore, this study provides a PfHsp70-PfJDP drug-target platform for further studies on other drug-repurposing small molecule libraries. However, given the predictive nature of this virtual screening study, the potential small molecule modulators need to be tested for their biochemical activity in Hsp70-JDP binding studies and chaperone assays, and their effect on the growth of malaria parasites using *in vitro* and *in vivo* assay. In addition, while the protein-protein docking conducted in this study was based on experimental Hsp70-JDP complexes, solving the crystal structure of the PfHsp70-PfJDP complexes would greatly accelerate further drug-discovery studies of this system.

## 5 Conclusion

Using virtual screening strategies, a diversity of drug-like compounds were identified which showed affinity and specificity for binding to PfHsp70-PfJDP complexes known to be important for the survival and pathogenicity of the malarial parasite. We were able to identify compounds with differential binding affinity for PfHsp70-x-PFE0055c, PfHsp70-2-PfSec63 and J domains of both PFE0055c and PfSec63 as compared to their human counterparts. Overall, these virtual screening strategies have identified a number of potential small molecule disruptors of these PfHsp70-PfJDP complexes. The five top scoring compounds, with the highest differential binding affinity and relevant docking poses, have been in advanced clinical studies, including the two FDA approved drugs, itraconazole and ezetimibe, which have previously been reported to have anti-malarial activity. This is the first time that such drug-like compounds have been identified as potential modulators of PfHsp70-PfJDP complexes, and they represent novel candidates for validation and development into anti-malarial drugs. Experimental validation of these compounds with respect to their inhibition of PfHsp70-PfJDP interaction, anti-malarial activity and cytotoxicity are a high priority for future studies.

## Data Availability

The datasets presented in this study can be found in online repositories. The names of the repository/repositories and accession number(s) can be found in the article/Supplementary Material.
