# Supplementary material for: In silico identification of modulators of J domain protein-Hsp70 interactions in Plasmodium falciparum: a drug repurposing strategy against malaria
Source: Front Mol Biosci. 2023 Aug 9;10:1158912. doi: 10.3389/fmolb.2023.1158912 (PMC10445141; doi:10.3389/fmolb.2023.1158912)
Supplement: Supplementary file 1 [file DataSheet1.pdf]

Supplementary Material

In silico identification of modulators of J domain protein-Hsp70 interactions in *Plasmodium falciparum*: A drug repurposing strategy against malaria

Harpreet Singh[1], Shaikha Y. Almaazmi[2], Tanima Dutta[3], Robert A. Keyzers[4], Gregory L. Blatch[2,5*]

[1]Department of Bioinformatics, Hans Raj Mahila Maha Vidyalaya, Jalandhar 144008, Punjab, India

[2]Biomedical Research and Drug Discovery Research Group, Faculty of Health Sciences, Higher Colleges ofTechnology, Sharjah, United Arab Emirates

[3]PathWest Nedlands, QEII Medical Centre, WA, Australia

[4]Centre for Biodiscovery & School of Chemical and Physical Sciences, Victoria University of Wellington, Wellington, New Zealand

[5]Biomedical Biotechnology Research Unit, Department of Biochemistry and Microbiology, Rhodes University, Grahamstown, South Africa

*** Correspondence:**
Gregory L. Blatch
gblatch@hct.ac.ae; g.blatch@ru.ac.za

Data availability statement:

All data sets are available in the published article and these associated Supplementary Materials.

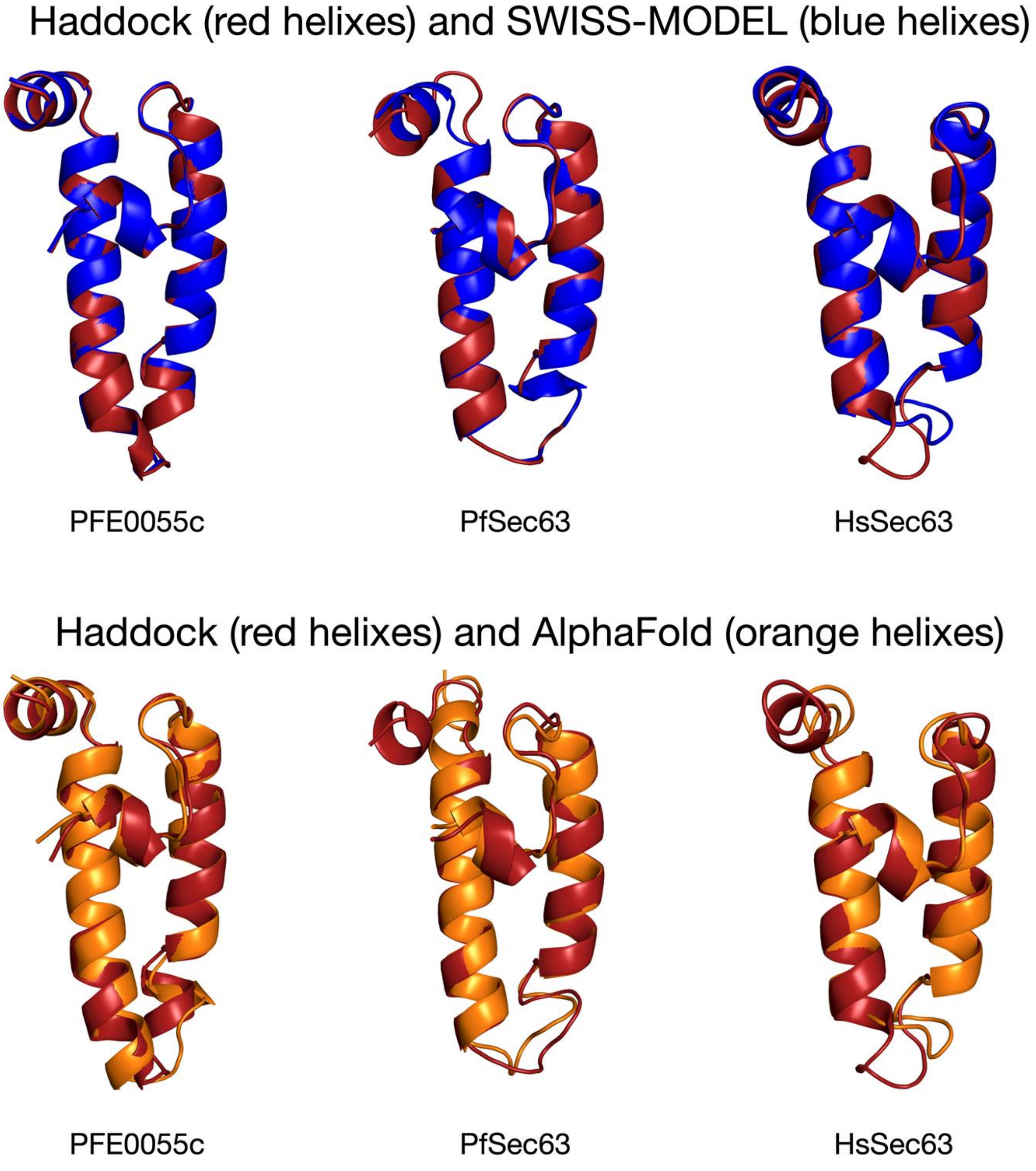

Figure S1. Three dimensional models of the J domains of PFE0055c, PfSec63, and HsSec63 generated using alternative software and shown superimposed onto HADDOCK models used in the docking screening. The models were prepared using SWISS-MODEL (blue helixes) (Waterhouse et al., 2018) and AlphaFold (orange helixes) (Jumper et al., 2021) to validate the HADDOCK models used in the docking screening (dark red helixes) (Dominguez et al. 2003; Van Zundert et al. 2016) and graphically rendered using PyMol 2.5.2 (PyMOL Molecular Graphics System, Version 2.0 Schrödinger, LLC).

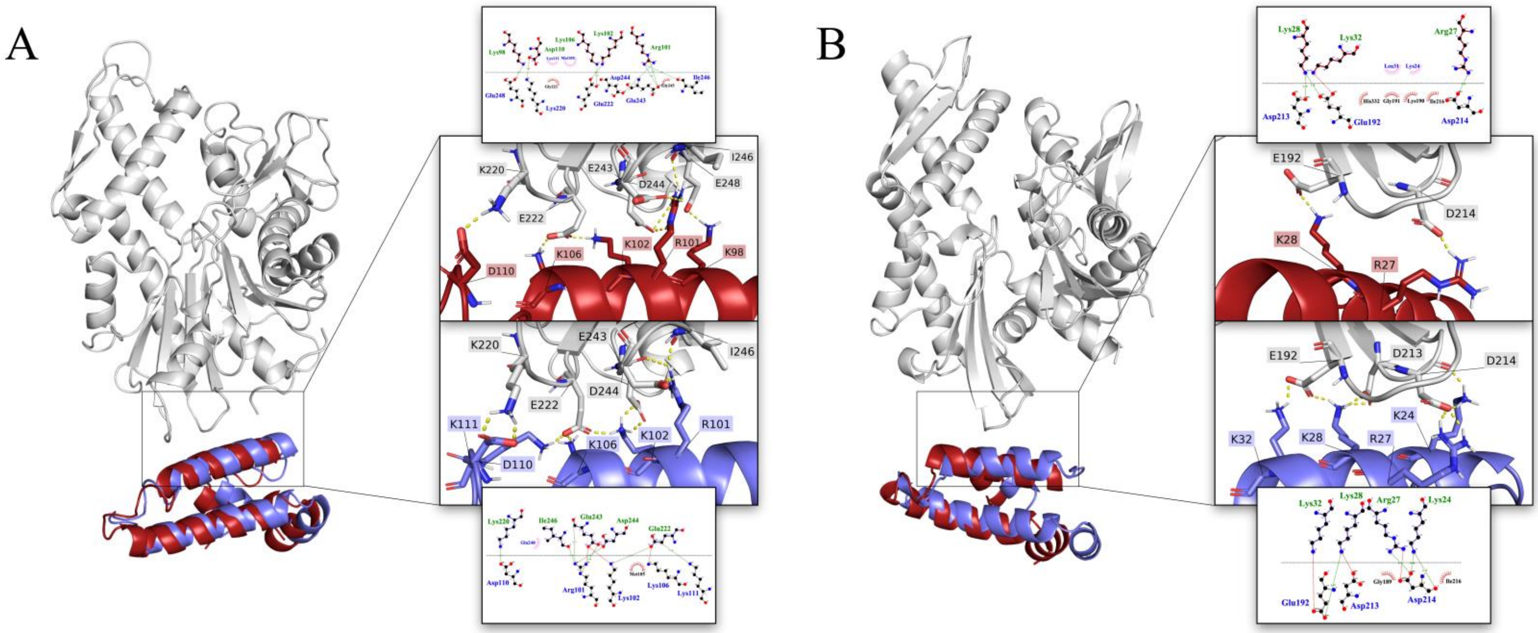

Figure S2. Three dimensional structures of the HADDOCK complexes used in the docking screening (J domain; red helixes) superimposed on the complex generated by ClusPro online server (J domain; blue helixes)(Desta et al. 2020; Vajda et al. 2017; Kozakov et al. 2017; Kozakov et al. 2013). (A) PfHsp70-x-PFE00055c complex (B) HsHsp70-DNAJA1 complex. The zoom-in on the right side of each protein structure showthe interacting residues, with yellow dotted lines representing the hydrogen bonds. The complexes weregraphically rendered using PyMol 2.5.2 (PyMOL Molecular Graphics System, Version 2.0 Schrödinger, LLC).The LigPlot+ contact analyses (Laskowski and Swindells, 2011) accompanied with the zoom-in figures shows the protein-protein interaction diagrams. The HADDOCK complex J domain bonds are shown with thick red lines, ClusPro complex J domain bonds are shown with blue lines, NBD bonds are shown with grey lines, nitrogen and oxygen are highlighted with blue and red colors dots, respectively, hydrogen bonds are shown by green dashed lines with the length of the bond printed in the middle, salt bridges shown by red dashed lines and hydrophobic contacts between proteins are indicated by the brick-red spoked arcs for NBD and pink spoked arcs for J domain.

Table S1: Quality parameters for the NBD-J domain complexes and the J domain structures using SAVES v6.0 web server utilizing ERRAT, VERIFY3D and PROCHECK structure validation programs.

**Structure**	**Score/Result**		
	**ERRAT**	**VERIFY 3D**	**PROCHECK (Ramachandran Plot)**
PfHsp70-x-PFE0055c	95.05	Pass (83.04% of the residues have averaged 3D-1D score >= 0.1)	Pass without errors (89.5 % core, 10.5% allowed, 0% in disallowed regions)
HsHsp70-DNAJA1	92	Pass (84.84% of the residues have averaged 3D-1D score >= 0.1)	Pass without errors (90.8 % core, 9.2% allowed, 0% in disallowed regions)
PfHsp70-2-PfSec63 J domain	94.12	Pass (84.84% of the residues have averaged 3D-1D score >= 0.1)	Pass without errors (89.2 % core, 10.8% allowed, 0% in disallowed regions)
HsHspGrp78-HsSec63 J domain	94.33	Pass (89.77% of the residues have averaged 3D-1D score >= 0.1)	Pass without errors (90.6 % core, 9.4% allowed, 0% in disallowed regions)
PFE0055c J domain	95	Pass (97.06% of the residues have averaged 3D-1D score >= 0.1)	Pass without errors (98.4% core, 1.6% allowed, 0% in disallowed regions)
DNAJA1A J domain	100	Pass (100% of the residues have averaged 3D-1D score >= 0.1)	Pass without errors (94.5 % core, 5.5% allowed, 0% in disallowed regions)
PfSec63 J domain	95	Pass (82% of the residues have averaged 3D-1D score >= 0.1)	Pass without errors (94.5 % core, 6.5% allowed, 0% in disallowed regions)
HsSec63 J domain	94.32	(83.05% of the residues have averaged 3D-1D score >= 0.1)	Pass without errors (92.2 % core, 7.8% allowed, 0% in disallowed regions)

Table S2. Summary of the top ranked compound dockings with differential binding affinity for PfHsp70-x-PFE0055c over PfHsp70-x-DNAJA1. Docking was conducted on NBD-J domain complexes.

Pubchem Compound ID (CID)	Common Name	PRB Disease Indication	Binding Affinity of the top ranked conformation (kcal/mol)			
			PfHsp70-x-PFE0055c	PfHsp70-x-DNAJA1	HsHsp70-PFE0055c	HsHsp70-DNAJA1
**5834620**	Chalcone, C86	Anti-cancer	-5.5	-5.7	-6.2	-5.5
**833990**	-	Anti-viral	-6.7	-5.3	-6.2	-5.8
**9819085**	-	Anti-viral	-6.7	-6.2	-6.9	-5.8
**10046204**	-	Anti-viral	-6.6	-6.8	-7.0	-5.8
**60196939**	-	Anti-bacterial	-7.2	-5.4	-6.9	-6.6
**2862146**	MBX 1641	Anti-bacterial	-6.6	-5.5	-6.6	-6.0

Table S3: Details of binding affinity of individual poses for the top ranked compound dockings with PfHsp70-x-PFE0055c and HsHsp70-DNAJA1 complexes. Docking was conducted on NBD-J domain complexes.

Ligand (Pubchem CID)	Pose	Binding Affinity (kcal/mol)	
		**PfHsp70-x-PFE0055c**	**HsHsp70-DNAJA1**
**833990**	1	-6.7	-5.8
	2	-6.3	-5.7
	3	-6.2	-5.6
	4	-6.2	-5.6
	5	-6.1	-5.4
	6	-6.1	-5.4
	7	-5.9	-5.4
	8	-5.9	-5.3
	9	-5.9	-5.3
**9819085**	1	-6.7	-5.8
	2	-6.3	-5.7
	3	-6.3	-5.7
	4	-6.2	-5.7
	5	-6.2	-5.7
	6	-6.1	-5.6
	7	-6	-5.6
	8	-6	-5.6
	9	-5.9	-5.6
**7658**	1	-5	-4.2
	2	-4.8	-4
	3	-4.8	-3.9
	4	-4.8	-3.9
	5	-4.7	-3.9
	6	-4.6	-3.8
	7	-4.6	-3.8
	8	-4.6	-3.7

	9	-4.6	-3.6
**10046204**	1	-6.6	-5.8
	2	-6.5	-5.6
	3	-6.5	-5.5
	4	-6.4	-5.5
	5	-6.3	-5.5
	6	-6.2	-5.3
	7	-6.1	-5.3
	8	-6	-5.2
	9	-5.9	-5.2
**464205**	1	-5.4	-4.7
	2	-5.2	-4.7
	3	-4.8	-4.6
	4	-4.8	-4.6
	5	-4.8	-4.6
	6	-4.7	-4.6
	7	-4.7	-4.6
	8	-4.6	-4.5
	9	-4.5	-4.5
**14570151**	1	-6.7	-6
	2	-5.3	-5.7
	3	-5.3	-5.5
	4	-5.3	-5.4
	5	-5.3	-5.4
	6	-5.3	-5.3
	7	-5.3	-5.3
	8	-5.3	-5.2
	9	-5.3	-5.2
**60196939**	1	-7.2	-6.6
	2	-7.1	-6.3
	3	-7	-6
	4	-7	-5.9
	5	-7	-5.7
	6	-6.9	-5.5
	7	-6.7	-5.5
	8	-6.7	-5.5
	9	-6.7	-5.4
**802112**	1	-6.5	-5.9
	2	-6	-5.8
	3	-5.9	-5.7
	4	-5.8	-5.5
	5	-5.6	-5.5
	6	-5.5	-5.4
	7	-5.4	-5.4
	8	-5.3	-5.4

	9	-5.2	-5.3
**2862146**	1	-6.6	-6
	2	-6.4	-5.9
	3	-5.9	-5.9
	4	-5.9	-5.7
	5	-5.9	-5.7
	6	-5.8	-5.6
	7	-5.8	-5.6
	8	-5.8	-5.4
	9	-5.8	-5.4
**104943**	1	-6.5	-5.9
	2	-6.1	-5.9
	3	-6.1	-5.8
	4	-6	-5.8
	5	-6	-5.7
	6	-5.9	-5.5
	7	-5.8	-5.5
	8	-5.8	-5.5
	9	-5.6	-5.4
**71812237**	1	-5.6	-5
	2	-5.3	-5
	3	-5.3	-5
	4	-5.2	-4.9
	5	-5.1	-4.9
	6	-5.1	-4.8
	7	-5	-4.8
	8	-4.9	-4.8
	9	-4.9	-4.7
**C86**	1	-5.5	-5.5
	2	-5.5	-5.4
	3	-5.5	-5.3
	4	-5.4	-5.2
	5	-5.4	-5.2
	6	-5.4	-5.1
	7	-5.4	-5.1
	8	-5.3	-5
	9	-5.3	-5

Table S4: Structural details of the top ranked compound dockings with differential binding affinity for both PfHsp70-x-PFE0055c and PfHsp70-x-DNAJA1 complexes. Docking was conducted on NBD-J domain complexes.

Compound IDs	Common Name	PRB Disease Indication	2D Structure/ Chemical Name	Binding Affinity of the Top ranked conformation (kcal/mol)			
				PfHsp70-x-PFE0055c	PfHsp70-x-DNAJA1	HsHsp70-PFE0055c	HsHsp70-DNAJA1
5834620<br>CHEMBL32856	Chalcone, C86	Anticancer	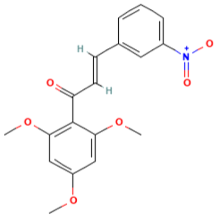<br>(2E)-3-(3-nitrophenyl)-1-(2,4,6-trimethoxyphenyl)prop-2-en-1-one	-5.5	-5.7	-6.2	-5.5
833990<br>CHEMBL1465049<br>MMV1580504	-	Antviral	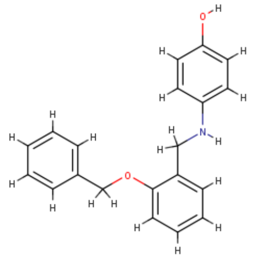<br>4-[(2-phenylmethoxyphenyl)methylamino]phenol	-6.7	-5.3	-6.2	-5.8
9819085<br>CHEMBL106454<br>MMV1633963	-	Antiviral	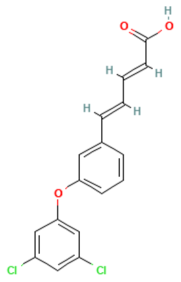<br>(2E,4E)-5-[3-(3,5-dichlorophenoxy)phenyl]penta-2,4-dienoic acid	-6.7	-6.2	-6.9	-5.8

10046204 CHEMBL285833 MMV1634556	-	Antviral	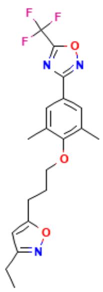 3-[4-[3-(3-ethyl-1,2-oxazol-5-yl)propoxy]-3,5-dimethylphenyl]-5-(trifluoromethyl)-1,2,4-oxadiazole	-6.6	-6.8	-7	-5.8
60196939 CHEMBL2365952 MMV1593540	-	Antibacterial	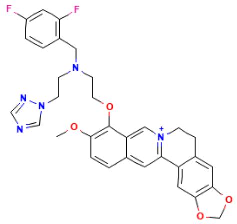 N-[(2,4-difluorophenyl)methyl]-N-[2-[(17-methoxy-5,7-dioxa-13-azoniapentacyclo[11.8.0.02,10.04,8.015,20]henicosa-1(21),2(10),3,8,13,15,17,19-octaen-16-yl)oxy]ethyl]-2-(1,2,4-triazol-1-yl)ethanamine	-7.2	-5.4	-6.9	-6.6
2862146 CHEMBL3398004 MMV1578925	MBX 1641	Antibacterial	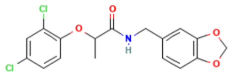 N-(1,3-benzodioxol-5-ylmethyl)-2-(2,4-dichlorophenoxy)propanamide	-6.6	-5.5	-6.6	-6

Table S5. Summary of the top ranked compound dockings for the ER complexes with differential binding affinity for PfHsp70-2-PfSec63 over HsGRP78-HsSec63. Docking was conducted on NBD-J domain complexes.

Pubchem Compound ID (CID)	Common Name	PRB Disease Indication	Binding Affinity of the top ranked conformation (kcal/mol)	
			PfHsp70-2-PfSec63	HsGRP78-HsSec63
**5834620**	Chalcone, C86	Anti-cancer	-5.9	-6.4
**60196939**	-	Anti-bacterial	-7.3	-5.6
**6098**	Cloxacillin	Anti-bacterial	-7.5	-6.0
**57383474**	-	Anti-bacterial	-7.1	-5.9
**10324367**	Boceprevir	Anti-viral	-7.4	-6.3
**5189681**	-	Anti-viral	-7.3	-6.2
**137648729**	-	Anti-fungal	-8.1	-7.1
**76685216**	Zoliflodacin	Anti-bacterial	-9.0	-8.1
**45138674**	Letermovir	Anti-viral	-7.0	-6.1
**467825**	Ravuconazole	Anti-fungal	-7.3	-6.4
**55283**	Itraconazole	Anti-fungal	-8.3	-7.5
**11485687**	-	Anti-viral	-7.4	-6.7

Table S6: Details of binding affinity of individual poses for the top ranked compound dockings with PfHsp70-2-PfSec63 and HsGRP78-HsSec63 complexes. Docking was conducted on NBD-J domain complexes.

Ligand (Pubchem CID)	Pose	Binding Affinity (kcal/mol)	
		**PfHsp70-2-PfSec63**	**HsGRP78-HsSe63**
**60196939**	1	-7.3	-5.6
	2	-6.6	-5.5
	3	-6.5	-5.5
	4	-6.5	-5.4
	5	-6.1	-5.4
	6	-5.9	-5.4
	7	-5.9	-5.1
	8	-5.8	-5.1
	9	-5.7	-5.1
**609**	1	-7.5	-6
	2	-6.5	-5.5
	3	-6.3	-5.5
	4	-6.2	-5.4
	5	-6.2	-5.3
	6	-6.1	-5.3
	7	-6	-5.2
	8	-5.8	-5.2
	9	-5.8	-5.2
**57383474**	1	-7.1	-5.9
	2	-7.1	-5.9
	3	-6.7	-5.8

	4	-6.6	-5.7
	5	-6.6	-5.7
	6	-6.4	-5.7
	7	-6.4	-5.6
	8	-6.2	-5.6
	9	-6.2	-5.6
**10324367**	1	-7.4	-6.3
	2	-7.2	-5.9
	3	-6.7	-5.5
	4	-6.6	-5.4
	5	-6.4	-5.4
	6	-6.4	-5.2
	7	-6.3	-5.1
	8	-6.3	-5.1
	9	-6.3	-5.1
**5189681**	1	-7.3	-6.2
	2	-6.8	-5.8
	3	-6.7	-5.7
	4	-6.7	-5.7
	5	-6.6	-5.6
	6	-6.5	-5.6
	7	-6.4	-5.5
	8	-6.4	-5.5
	9	-6.3	-5.5
**8392433**	1	-7.2	-6.2
	2	-6.6	-6.1
	3	-6.6	-6
	4	-6.6	-5.7
	5	-6.3	-5.6
	6	-6.3	-5.6
	7	-6.2	-5.6
	8	-6.2	-5.5
	9	-6.2	-5.4
**137648729**	1	-8.1	-7.1
	2	-8	-7.1
	3	-7.7	-6.9
	4	-7.5	-6.7
	5	-7.4	-6.7
	6	-7.3	-6.7
	7	-7.3	-6.7
	8	-7.2	-6.6
	9	-7.2	-6.6
**76685216**	1	-9	-8.1
	2	-8.6	-8
	3	-7.8	-7.9
	4	-7.6	-7.6

	5	-7.5	-7.6
	6	-7.5	-7.5
	7	-7.5	-7.4
	8	-7.5	-7.2
	9	-7.4	-7.2
**45138674**	1	-7	-6.1
	2	-6.9	-6.1
	3	-6.9	-6
	4	-6.8	-6
	5	-6.7	-6
	6	-6.6	-5.9
	7	-6.6	-5.9
	8	-6.5	-5.7
	9	-6.4	-5.6
**50942319**	1	-6.9	-6
	2	-6.6	-5.7
	3	-6.4	-5.6
	4	-6.4	-5.4
	5	-6.4	-5.3
	6	-6.2	-5.2
	7	-6.1	-5.1
	8	-6.1	-5.1
	9	-6	-5
**65781**	1	-6.4	-6.2
	2	-6.4	-6.2
	3	-6.3	-6
	4	-6.3	-5.9
	5	-6.2	-5.9
	6	-6.2	-5.8
	7	-6.1	-5.7
	8	-6.1	-5.6
	9	-6	-5.5
**467825**	1	-7.3	-6.4
	2	-6.9	-6.4
	3	-6.8	-6.4
	4	-6.7	-6.3
	5	-6.7	-6.1
	6	-6.7	-6
	7	-6.5	-5.9
	8	-6.5	-5.9
	9	-6.5	-5.9
**4106**	1	-5.9	-5.1
	2	-5.6	-5
	3	-5.6	-4.9
	4	-5.6	-4.9

	5	-5.4	-4.9
	6	-5.4	-4.7
	7	-5.4	-4.6
	8	-5.3	-4.6
	9	-5.2	-4.5
**55283**	1	-8.3	-7.5
	2	-8.2	-7.2
	3	-8.2	-7.2
	4	-8.1	-7.1
	5	-7.8	-7
	6	-7.7	-6.9
	7	-7.6	-6.8
	8	-7.5	-6.7
	9	-7.5	-6.7
**23730143**	1	-8.8	-8
	2	-8.4	-7.9
	3	-8.3	-7.8
	4	-8.2	-7.6
	5	-8.1	-7.6
	6	-8.1	-7.4
	7	-8	-7.2
	8	-7.9	-7.1
	9	-7.9	-7.1
**64971**	1	-7.1	-6.3
	2	-7.1	-6.2
	3	-7.1	-6.1
	4	-7	-6
	5	-6.7	-6
	6	-6.7	-6
	7	-6.6	-5.9
	8	-6.5	-5.9
	9	-6.4	-5.8
**55245**	1	-6.7	-5.9
	2	-6.7	-5.5
	3	-6.5	-5.5
	4	-6.3	-5.5
	5	-6.1	-5.4
	6	-6	-5.4
	7	-5.9	-5.1
	8	-5.7	-5.1
	9	-5.7	-5.1
**470766**	1	-6.8	-6.1
	2	-6.8	-6
	3	-6.5	-6
	4	-6.5	-6
	5	-6.5	-6

	6	-6.5	-5.7
	7	-6.5	-5.7
	8	-6.4	-5.4
	9	-6.4	-5.4
**11485687**	1	-7.4	-6.7
	2	-7.4	-6.7
	3	-7.3	-6.7
	4	-7.1	-6.6
	5	-7	-6.6
	6	-6.8	-6.6
	7	-6.8	-6.5
	8	-6.7	-6.3
	9	-6.7	-6.1
**493469**	1	-6.4	-5.7
	2	-6.4	-5.4
	3	-6.4	-5.4
	4	-6.2	-5.4
	5	-6.1	-5.3
	6	-6	-5.3
	7	-5.9	-5.2
	8	-5.9	-5.2
	9	-5.8	-5.1
**21755317**	1	-5.7	-5
	2	-5.7	-5
	3	-5.5	-4.7
	4	-5.3	-4.7
	5	-5.3	-4.6
	6	-5.2	-4.5
	7	-5.1	-4.4
	8	-4.9	-4.3
	9	-4.9	-4.3
**4211702**	1	-7.1	-6.4
	2	-7	-6.4
	3	-6.7	-6.2
	4	-6.6	-6.2
	5	-6.5	-6
	6	-6.4	-6
	7	-6.4	-5.9
	8	-6.3	-5.9
	9	-6.2	-5.7
**C86**	1	-5.9	-6.4
	2	-5.9	-5.8
	3	-5.8	-5.6
	4	-5.8	-5.5
	5	-5.7	-5.5

6	-5.6	-5.5
7	-5.5	-5.4
8	-5.5	-5.2
9	-5.5	-5.2

Table S7: Structural details of the top ranked compound dockings with differential binding affinity for ERspecific PfHsp70-2-PfSec63 and HsGRP78-HsSec63 complexes. Docking was conducted on NBD-J domain complexes.

Compound IDs	Common Name	PRB Disease Indication	2D Structure/ Chemical Name	Binding Affinity of the top ranked conformation (kcal/mol)	
				PfHsp70-2-PfSec63	HsGRP78-HsSec63
5834620<br>CHEMBL32856	Chalcone, C86	Anticancer	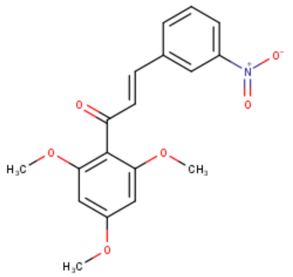<br>(2E)-3-(3-nitrophenyl)-1-(2,4,6-trimethoxyphenyl)prop-2-en-1-one	-5.9	-6.4
60196939<br>CHEMBL2365952<br>MMV1593540	-	Antibacterial	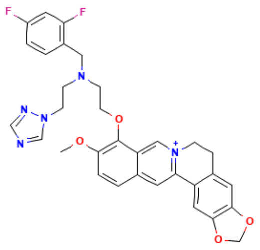<br>N-[(2,4-difluorophenyl)methyl]-N-[2-[(17-methoxy-5,7-dioxa-13-azoniapentacyclo[11.8.0.02,10.04,8.0 15,20]henicosa-1(21),2(10),3,8,13,15,17,19-octaen-16-yl)oxy]ethyl]-2-(1,2,4-triazol-1-yl)ethanamine	-7.3	-5.6
6098<br>CHEMBL891<br>MMV002740	Cloxacillin	Antibacterial	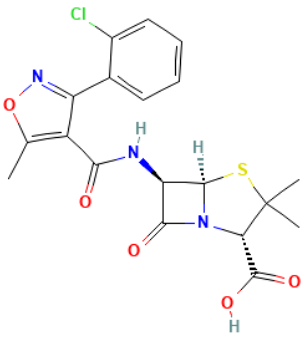<br>rac-(2R,5S,6S)-6-[[3-(2-chlorophenyl)-5-methyl-1,2-oxazole-	-7.5	-6

			4-carbonyl]amino]-3,3-dimethyl-7-oxo-4-thia-1-azabicyclo[3.2.0]heptane-2-carboxylate		
57383474<br>CHEMBL2088120<br>MMV1581559	-	Antibacterial	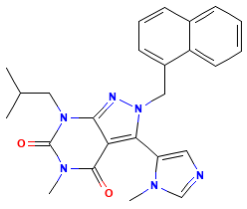<br>5-methyl-3-(3-methylimidazol-4-yl)-7-(2-methylpropyl)-2-(naphthalen-1-ylmethyl)pyrazolo[3,4-d]pyrimidine-4,6-dione	-7.1	-5.9
10324367<br>CHEMBL218394<br>MMV1580497	Boceprevir	Antiviral	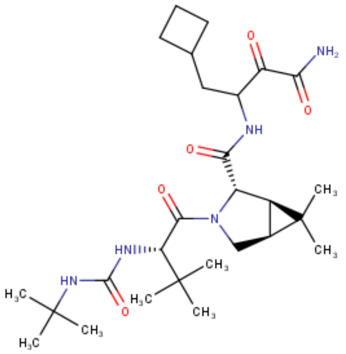<br>(1R,2S,5S)-N-(4-amino-1-cyclobutyl-3,4-dioxobutan-2-yl)-3-[(2S)-2-(tert-butylcarbamoylamino)-3,3-dimethylbutanoyl]-6,6-dimethyl-3-azabicyclo[3.1.0]hexane-2-carboxamide	-7.4	-6.3
5189681<br>CHEMBL1459098<br>MMV1165877	-	Antiviral	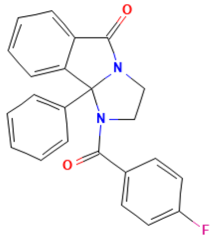<br>1-(4-fluorobenzoyl)-9b-phenyl-2,3-dihydroimidazo[2,1-a]isoindol-5-one	-7.3	-6.2
137648729<br>CHEMBL4085632<br>MMV1782110	-	Antifungal	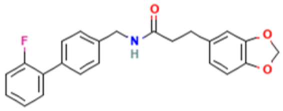<br>3-(1,3-benzodioxol-5-yl)-N-[[4-(2-fluorophenyl)phenyl]methyl]propanamide	-8.1	-7.1

76685216<br>CHEMBL3544978<br>MMV1578557	Zoliflodacin	Antbacterial	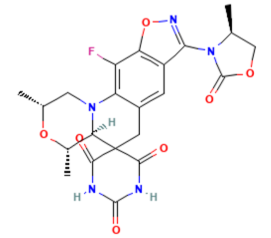<br>1-[(3-amino-2-methylphenyl)methyl]-4-(2-thiophen-2-ylethoxy)pyridin-2-one	-9	-8.1
45138674<br>CHEMBL1241951<br>MMV690653	Letermovir	Antiviral	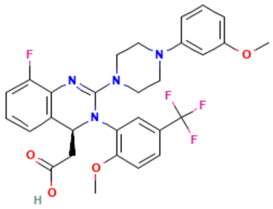<br>2-[(4S)-8-fluoro-2-[4-(3-methoxyphenyl)piperazin-1-yl]-3-[2-methoxy-5-(trifluoromethyl)phenyl]-4H-quinazolin-4-yl]acetic acid	-7	-6.1
467825<br>CHEMBL294029<br>MMV1634362	Ravuconazole	Antifungal	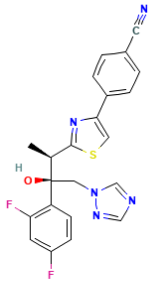<br>4-[2-[(2R,3R)-3-(2,4-difluorophenyl)-3-hydroxy-4-(1,2,4-triazol-1-yl)butan-2-yl]-1,3-thiazol-4-yl]benzonitrile	-7.3	-6.4
55283<br>CHEMBL22587<br>MMV637528	Itraconazole	Antifungal	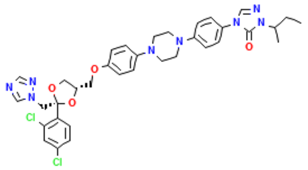<br>2-butan-2-yl-4-[4-[4-[4-[[2-(2,4-dichlorophenyl)-2-(1,2,4-triazol-1-ylmethyl)-1,3-dioxolan-4-yl]methoxy]phenyl]piperazin-1-yl]phenyl]-1,2,4-triazol-3-one	-8.3	-7.5
11485687<br>CHEMBL1242629<br>MMV1782114	-	Antiviral	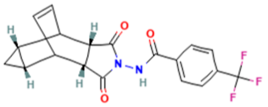<br>N-(3,5-dioxo-4-azatetracyclo[5.3.2.02,6.08,10]dodec-11-en-4-yl)-4-(trifluoromethyl)benzamide	-7.4	-6.7

Table S8. Summary of the top ranked compound dockings with differential binding affinity for PfJDPs.

Pubchem Compound ID (CID)	Common Name	PRB Disease Indication	Binding Affinity of the top ranked conformation (kcal/mol)			
			PFE0055c	DNAJA1	PfSec63	HsSec63
**Differential affinity across Cytosolic (PFE0055c) and ER (PfSec63) J domains**						
**5834620**	Chalcone, C86	Anti-cancer	-6.0	-5.3	-5.3	5.3
**150311**	Ezetimibe	Anti-viral	-7.6	-5.7	-6.1	-5.0
**2867190**	Benzo-diazepinone	Anti-viral	-8.4	-6.6	-7.3	-6.5
**10077130**	Vorapaxar	Anti-viral	-7.9	-6.7	-6.8	-6.0
**1317590**	-	Anti-viral	-7.0	-5.8	-6.0	-5.2
**9911469**	FK-788	Anti-viral	-7.1	-5.9	-6.6	-5.5
**Preferential Binding Affinities for Cytosolic J (PFE0055c)**						
**8392433**	BRD-K92790413	Anti-bacterial	-7.0	-5.3	-5.3	-5.8
**1684**	Pleconaril	Anti-viral	-6.8	-5.3	-6.1	-5.4
**155546288**	-	Anti-fungal	-7.6	-6.1	-6.5	-5.9
**10027278**	OSU-03012	Anti-fungal	-8.0	-6.6	-6.7	-6.1
**2768133**	-	Anti-viral	-7.6	-6.2	-6.0	-6.0
**76310291**	-	Anti-bacterial	-7.6	-6.2	-5.8	-5.9
**115358**	Tafenoquine	Anti-bacterial	-6.4	-5.1	-5.5	-5.2
**3783668**	-	Anti-viral	-7.7	-6.4	-6.9	-6.3
**51354502**	-	Anti-viral	-8	-6.7	-6.9	-6.6
**71450388**	-	Anti-bacterial	-8	-6.7	-6.2	-6.0
**55245**	Mifepristone	Anti-viral	-7.3	-6.0	-6.3	-5.6
**1158563**	-	Anti-bacterial	-7.5	-6.2	-6.0	-6.9
**16739062**	TTP 8307	Anti-viral	-9.0	-7.7	-7.9	-7.5
**155541425**	-	Anti-fungal	-7.8	-6.5	-6.7	-6.7
**2763159**	-	Anti-bacterial	-6.0	-4.8	-5.4	-5.0
**56668933**	-	Anti-viral	-7.3	-6.1	-6.1	-5.8

Table S9: Details of binding affinity of individual poses for compounds showing differential binding affinity against cytosolic as well as ER-specific J domains.

Ligand (Pubchem CID)	Pose	Binding Affinity (kcal/mol)			
		**PFE0055c**	**DNAJA1**	**PfSec63**	**HsSec63**
**150311**	1	-7.6	-5.7	-6.1	-5
	2	-7.1	-5.6	-5.9	-5
	3	-7.1	-5.4	-5.8	-4.9
	4	-7.1	-5.3	-5.7	-4.7
	5	-7.1	-5.3	-5.6	-4.7
	6	-6.9	-5.3	-5.6	-4.7
	7	-6.8	-5.1	-5.5	-4.5
	8	-6.7	-5.1	-5.5	-4.4
	9	-6.7	-5	-5.4	-4.3
**2867190**	1	-8.4	-6.6	-7.3	-6.5
	2	-7.7	-6.5	-6.4	-6.4

	3	-7.5	-6.5	-6.4	-6.2
	4	-7.4	-6.5	-6.4	-6
	5	-7.1	-6.5	-6.3	-5.9
	6	-7.1	-6.5	-6.3	-5.8
	7	-6.8	-6.3	-6.3	-5.8
	8	-6.7	-6.3	-6.1	-5.6
	9	-6.5	-6.2	-6.1	-5.6
**10077130**	1	-7.9	-6.7	-6.8	-6
	2	-7.6	-6.5	-6.5	-6
	3	-7.5	-6.5	-6.4	-5.9
	4	-7.5	-6.3	-6.3	-5.9
	5	-7	-6.1	-6.1	-5.9
	6	-6.9	-6	-6	-5.7
	7	-6.8	-6	-6	-5.7
	8	-6.6	-6	-6	-5.6
	9	-6.4	-5.7	-6	-5.6
**1317590**	1	-7	-5.8	-6	-5.2
	2	-6.4	-5.7	-5.8	-5.2
	3	-6.4	-5.7	-5.6	-5.1
	4	-6.4	-5.7	-5.5	-5
	5	-6.3	-5.6	-5.4	-5
	6	-6.2	-5.5	-5.4	-5
	7	-6.2	-5.4	-5.4	-5
	8	-6.2	-5.3	-5.2	-4.9
	9	-6.1	-5.3	-5.2	-4.9
**9911469**	1	-7.1	-5.9	-6.6	-5.5
	2	-7	-5.8	-6	-5.3
	3	-7	-5.6	-5.6	-5.3
	4	-6.9	-5.5	-5.5	-5.3
	5	-6.9	-5.5	-5.5	-5.3
	6	-6.7	-5.3	-5.5	-5.3
	7	-6.7	-5.3	-5.4	-5.3
	8	-6.7	-5.3	-5.4	-5.2
	9	-6.5	-5.3	-5.3	-5.2

Table S10: Details of binding affinity of individual poses for compounds showing differential binding affinity against cytosolic J domains.

Ligand (Pubchem CID)	Pose	Binding Affinity (kcal/mol)			
		**PFE0055c**	**DNAJA1**	**PfSec63**	**HsSec63**
**8392433**	1	-7	-5.3	-5.3	-5.8
	2	-6.6	-5	-5.1	-5.5
	3	-6.5	-4.9	-4.9	-5.5
	4	-6.4	-4.9	-4.9	-5.4
	5	-6.1	-4.9	-4.8	-5.3
	6	-6	-4.9	-4.7	-5.3
	7	-6	-4.8	-4.7	-5.3
	8	-6	-4.8	-4.7	-5.2
	9	-5.9	-4.8	-4.7	-5.2
**1684**	1	-6.8	-5.3	-6.1	-5.4
	2	-6.7	-5.2	-5.8	-5.4
	3	-6.6	-5.1	-5.7	-5.2
	4	-6.6	-5	-5.7	-5.2
	5	-6.6	-5	-5.7	-5
	6	-6.4	-5	-5.6	-4.9
	7	-6.3	-5	-5.6	-4.9
	8	-6.2	-4.9	-5.5	-4.9
	9	-6.2	-4.9	-5.5	-4.9
**155546288**	1	-7.6	-6.1	-6.5	-5.9
	2	-7.5	-6.1	-6.1	-5.8
	3	-7.3	-5.9	-6.1	-5.6
	4	-7.3	-5.9	-6	-5.6
	5	-7	-5.9	-5.8	-5.4
	6	-6.6	-5.7	-5.7	-5.2
	7	-6.5	-5.7	-5.7	-5.2
	8	-6.4	-5.6	-5.6	-5.1
	9	-6.4	-5.5	-5.4	-5
**10027278**	1	-8	-6.6	-6.7	-6.1
	2	-7.3	-6.5	-6.6	-5.9
	3	-6.4	-6.5	-6.3	-5.8
	4	-6.4	-6.2	-6.2	-5.7
	5	-6.4	-6	-6.2	-5.7
	6	-6.2	-6	-6.1	-5.5
	7	-6.2	-6	-6	-5.5
	8	-6.1	-6	-5.9	-5.4
	9	-6.1	-5.9	-5.9	-5.4
**2768133**	1	-7.6	-6.2	-6	-6
	2	-6.4	-6.1	-6	-5.6
	3	-6.4	-6	-6	-5.5
	4	-6.4	-5.9	-5.9	-5.4

	5	-6.2	-5.9	-5.7	-5.4
	6	-6.2	-5.9	-5.7	-5.4
	7	-5.8	-5.9	-5.7	-5.4
	8	-5.7	-5.8	-5.6	-5.3
	9	-5.7	-5.7	-5.6	-5.3
**76310291**	1	-7.6	-6.2	-5.8	-5.9
	2	-7.4	-6.2	-5.8	-5.5
	3	-7	-6	-5.7	-5.5
	4	-6.5	-5.9	-5.5	-5.3
	5	-6.4	-5.9	-5.5	-5.2
	6	-6.4	-5.9	-5.5	-5.2
	7	-6.3	-5.6	-5.5	-5
	8	-6.2	-5.5	-5.4	-4.9
	9	-6.1	-5.4	-5.3	-4.9
**115358**	1	-6.4	-5.1	-5.5	-5.2
	2	-6	-5	-5.2	-4.9
	3	-5.8	-5	-5.2	-4.9
	4	-5.8	-5	-5.2	-4.8
	5	-5.6	-4.9	-5.1	-4.8
	6	-5.4	-4.9	-5.1	-4.8
	7	-5.3	-4.9	-5.1	-4.8
	8	-5.2	-4.9	-5	-4.7
	9	-5.2	-4.8	-5	-4.7
**3783668**	1	-7.7	-6.4	-6.9	-6.3
	2	-7.3	-6.3	-6.7	-6.3
	3	-7.3	-6.3	-6.6	-5.9
	4	-7.2	-6.1	-6.3	-5.8
	5	-7.1	-6.1	-6.3	-5.7
	6	-7	-6	-6.3	-5.6
	7	-6.9	-6	-6.2	-5.5
	8	-6.4	-5.9	-6.2	-5.5
	9	-6.4	-5.9	-6.1	-5.5
**51354502**	1	-8	-6.7	-6.9	-6.6
	2	-7.4	-6.7	-6.5	-6.5
	3	-7.2	-6.6	-6.4	-6.4
	4	-6.7	-6.6	-6.4	-6.4
	5	-6.6	-6.6	-6.4	-6
	6	-6.6	-6.5	-5.8	-6
	7	-6.5	-6.4	-5.8	-5.9
	8	-6.5	-6.4	-5.8	-5.9
	9	-6.4	-6.3	-5.7	-5.8
**71450388**	1	-8	-6.7	-6.2	-6
	2	-7.4	-6.4	-6.1	-5.9
	3	-7.1	-6.4	-6.1	-5.9
	4	-7.1	-6.4	-6.1	-5.9

	5	-6.8	-6.4	-6	-5.9
	6	-6.8	-6.2	-6	-5.8
	7	-6.7	-6.1	-6	-5.8
	8	-6.7	-6.1	-5.9	-5.7
	9	-6.7	-6.1	-5.9	-5.7
**55245**	1	-7.3	-6	-6.3	-5.6
	2	-6.1	-5.7	-5.9	-5.4
	3	-6.1	-5.6	-5.8	-5.3
	4	-6.1	-5.4	-5.6	-5.3
	5	-6	-5.3	-5.5	-5.3
	6	-6	-5.2	-5.4	-5.1
	7	-5.8	-5.2	-5.3	-5
	8	-5.8	-5.1	-5.3	-4.8
	9	-5.8	-5.1	-5.3	-4.8
**1158563**	1	-7.5	-6.2	-6	-6.9
	2	-7.3	-6.1	-5.8	-5.9
	3	-6.9	-5.8	-5.8	-5.8
	4	-6.9	-5.7	-5.8	-5.5
	5	-6.7	-5.7	-5.7	-5.5
	6	-6.7	-5.7	-5.7	-5.4
	7	-6.5	-5.7	-5.6	-5.4
	8	-6.5	-5.6	-5.6	-5.4
	9	-6.4	-5.6	-5.6	-5.4
**16739062**	1	-9	-7.7	-7.9	-7.5
	2	-8.9	-7.6	-7.5	-7.4
	3	-8.8	-7.6	-7.4	-7.3
	4	-8.7	-7.6	-7.4	-7.3
	5	-8.5	-7.5	-7.4	-7.2
	6	-8.4	-7.5	-7.3	-7.2
	7	-8.2	-7.4	-7.3	-7.1
	8	-8.1	-7.4	-7.2	-7
	9	-8.1	-7.4	-7.1	-7
**155541425**	1	-7.8	-6.5	-6.7	-6.7
	2	-7.7	-6.3	-6.6	-6.3
	3	-7.1	-6.1	-6.3	-6.3
	4	-6.6	-6	-6.2	-6.2
	5	-6.5	-5.9	-6.2	-5.9
	6	-6.5	-5.9	-6	-5.6
	7	-6.4	-5.8	-6	-5.6
	8	-6.3	-5.8	-6	-5.5
	9	-6.2	-5.7	-5.9	-5.5
**2763159**	1	-6	-4.8	-5.4	-5
	2	-5.8	-4.6	-4.8	-4.9
	3	-5.6	-4.6	-4.8	-4.8
	4	-5.4	-4.5	-4.7	-4.7

	5	-5.3	-4.5	-4.7	-4.7
	6	-5.3	-4.5	-4.6	-4.5
	7	-5.3	-4.4	-4.5	-4.4
	8	-5.3	-4.4	-4.5	-4.3
	9	-5.2	-4.3	-4.4	-4.2
**56668933**	1	-7.3	-6.1	-6.1	-5.8
	2	-6.4	-6	-5.9	-5.7
	3	-6.2	-6	-5.9	-5.4
	4	-6.1	-5.9	-5.6	-5.4
	5	-6	-5.9	-5.5	-5.3
	6	-6	-5.9	-5.5	-5.3
	7	-5.9	-5.8	-5.4	-5.3
	8	-5.7	-5.7	-5.4	-5.1
	9	-5.5	-5.7	-5.3	-5.1
**C86**	1	-6	-5.3	-5.3	-5.3
	2	-5.8	-5.2	-5.1	-5.1
	3	-5.8	-5.2	-5.1	-4.9
	4	-5.6	-5.1	-4.9	-4.9
	5	-5.6	-5.1	-4.9	-4.9
	6	-5.2	-5	-4.9	-4.9
	7	-5.2	-5	-4.8	-4.8
	8	-5.1	-4.9	-4.7	-4.8
	9	-5	-4.8	-4.7	-4.8

Table S11: Structural details of the top ranked compound dockings with differential binding affinity for *P. falciparum* J domains.

Compound IDs	Common Name	PRB Disease Indication	2D Structure/ Chemical Name	Binding Affinity of the Top ranked conformation (kcal/mol)			
				PFE0055c	DNAJA1	PfSec63	HsSec63
**Top binders with differential affinity across Cytosolic (PFE0055c) and ER (PfSec63) J domains**							
5834620<br>CHEMBL32856	Chalcone, C86	Anticancer	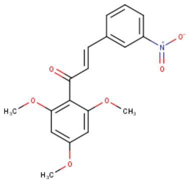<br>(2E)-3-(3-nitrophenyl)-1-(2,4,6-trimethoxyphenyl)prop-2-en-1-one	-6.0	-5.3	-5.3	5.3
150311<br>CHEMBL1138<br>MMV1581377	Ezetimibe	Antiviral	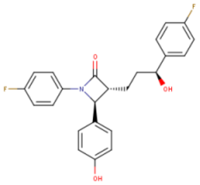<br>(3R,4S)-1-(4-fluorophenyl)-3-[(3S)-3-(4-fluorophenyl)-3-hydroxypropyl]-4-(4-hydroxyphenyl)azetidin-2-one	-7.6	-5.7	-6.1	-5.0
2867190<br>CHEMBL1673157<br>MMV1580503	Benzo-diazepinone	Antiviral	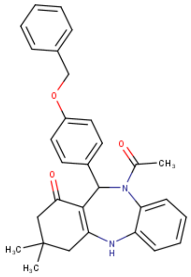<br>5-acetyl-9,9-dimethyl-6-(4-phenylmethoxyphenyl)-6,8,10,11-tetrahydrobenzo[b][1,4]benzodiazepin-7-one	-8.4	-6.6	-7.3	-6.5
10077130<br>CHEMBL493982<br>MMV1593515	Vorapaxar	Antiviral	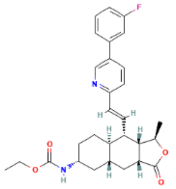<br>ethyl N-[(1R,6R,9S)-9-[(E)-2-[5-(3-fluorophenyl)pyridin-2-yl]ethenyl]-1-methyl-3-oxo-3a,4,4a,5,6,7,8,8a,9,9a-decahydro-1H-	-7.9	-6.7	-6.8	-6

			benzo[f][2]benzofuran-6-yl]carbamate				
1317590<br>CHEMBL1213418<br>MMV1580484	-	Antiviral	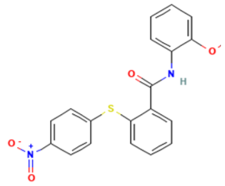<br>N-(2-methoxyphenyl)-2-(4-nitrophenyl)sulfanylbenzamide	-7	-5.8	-6	-5.2
9911469<br>CHEMBL196779<br>MMV1782352	FK-788	Antiviral	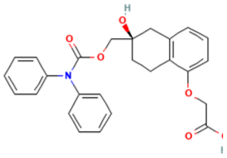<br>2-[[(6R)-6-(diphenylcarbamoyloxymethyl)-6-hydroxy-7,8-dihydro-5H-naphthalen-1-yl]oxy]acetic acid	-7.1	-5.9	-6.6	-5.5
**Preferential Binding Affinities for Cytosolic (PFE0055c)**							
8392433<br>CHEMBL1475828<br>MMV1578573	BRD-K92790413	Antibacterial	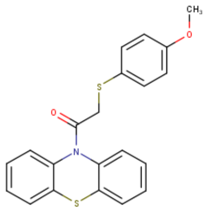<br>2-(4-methoxyphenyl)sulfanyl-1-phenothiazin-10-ylethanone	-7.0	-5.3	-5.3	-5.8
1684<br>CHEMBL29609<br>MMV690467	Pleconaril	Antiviral	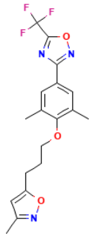<br>3-[3,5-dimethyl-4-[3-(3-methyl-1,2-oxazol-5-yl)propoxy]phenyl]-5-(trifluoromethyl)-1,2,4-oxadiazole	-6.8	-5.3	-6.1	-5.4
155546288<br>CHEMBL4531776<br>MMV1782218	-	Antifungal	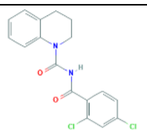<br>N-(2,4-dichlorobenzoyl)-3,4-dihydro-2H-quinoline-1-carboxamide	-7.6	-6.1	-6.5	-5.9

10027278<br>CHEMBL1650595<br>MMV1578560	OSU-03012	Antifungal	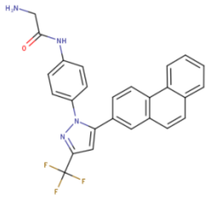<br>2-amino-N-[4-[5-phenanthren-2-yl-3-(trifluoromethyl)pyrazol-1-yl]phenyl]acetamide	-8.0	-6.6	-6.7	-6.1
2768133<br>CHEMBL3632725<br>MMV690555	-	Antiviral	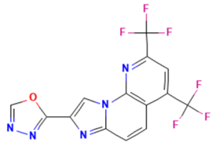<br>8-(1,3,4-Oxadiazol-2-yl)-2,4-bis(trifluoromethyl)imidazo[1,2-a][1,8]naphthyridine	-7.6	-6.2	-6	-6
76310291<br>CHEMBL3109593<br>MMV1582492	-	Antibacterial	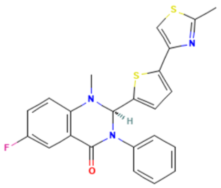<br>6-fluoro-1-methyl-2-[5-(2-methyl-1,3-thiazol-4-yl)thiophen-2-yl]-3-phenyl-2H-quinazolin-4-one	-7.6	-6.2	-5.8	-5.9
115358<br>CHEMBL298470<br>MMV000043	Tafenoquine	Antibacterial	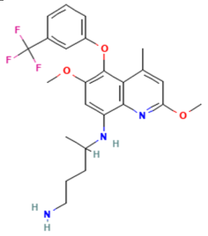<br>4-N-[2,6-dimethoxy-4-methyl-5-[3-(trifluoromethyl)phenoxy]quinolin-8-yl]pentane-1,4-diamine	-6.4	-5.1	-5.5	-5.2
3783668<br>CHEMBL548113<br>MMV019724	-	Antiviral	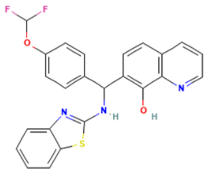<br>7-[(1,3-benzothiazol-2-ylamino)-[4-(difluoromethoxy)phenyl]methyl]quinolin-8-ol	-7.7	-6.4	-6.9	-6.3

51354502<br>CHEMBL2170001<br>MMV1634396	-	Antiviral	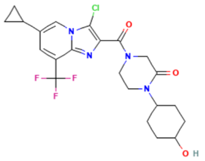<br>4-[3-chloro-6-cyclopropyl-8-(trifluoromethyl)imidazo[1,2-a]pyridine-2-carbonyl]-1-(4-hydroxycyclohexyl)piperazin-2-one	-8	-6.7	-6.9	-6.6
71450388<br>CHEMBL2203937<br>MMV1633965	-	Antibacterial	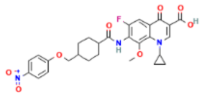<br>1-cyclopropyl-6-fluoro-8-methoxy-7-[[4-[(4-nitrophenoxy)methyl]cyclohexanecarbonyl]amino]-4-oxoquinoline-3-carboxylic acid	-8	-6.7	-6.2	-6
55245<br>CHEMBL1276308<br>MMV003297	Mifepristone	Antiviral	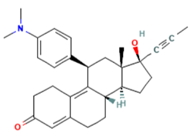<br>(8S,11R,13S,14S,17S)-11-[4-(dimethylamino)phenyl]-17-hydroxy-13-methyl-17-prop-1-ynyl-1,2,6,7,8,11,12,14,15,16-decahydrocyclopenta[a]phenanthren-3-one	-7.3	-6	-6.3	-5.6
1158563<br>CHEMBL1560266<br>MMV1578579	-	Antiacterial	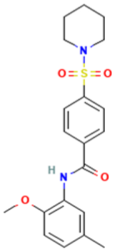<br>N-(2-methoxy-5-methylphenyl)-4-piperidin-1-ylsulfonylbenzamide	-7.5	-6.2	-6	-6.9
16739062<br>CHEMBL1163676<br>MMV1782211	TTP 8307	Antiviral	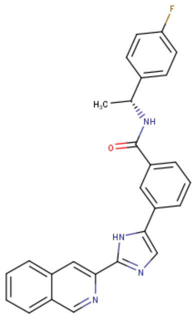<br>N-[(1R)-1-(4-fluorophenyl)ethyl]-3-(2-isoquinolin-3-yl-1H-	-9.0	-7.7	-7.9	-7.5

			imidazol-4-yl)benzamide				
155541425<br>CHEMBL4518698<br>MMV1782212	-	Antifungal	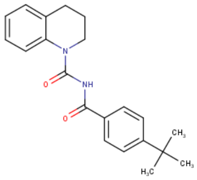<br>N-(4-tert-butylbenzoyl)-3,4-dihydro-2H-quinoline-1-carboxamide	-7.8	-6.5	-6.7	-6.7
2763159<br>CHEMBL2426244<br>MMV1578561	-	Antibacterial	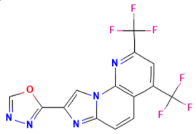<br>2-heptyl-3-hydroxy-1H-quinolin-4-one	-6	-4.8	-5.4	-5
56668933<br>CHEMBL1835916<br>MMV1634394	-	Antiviral	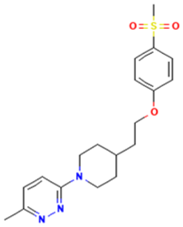<br>3-methyl-6-[4-[2-(4-methylsulfonylphenoxy)ethyl]piperidin-1-yl]pyridazine	-7.3	-6.1	-6.1	-5.8

Table S12. Top ranked compound testing for reproducibility of binding affinity and pose.

Pubchem Compound ID (CID)	Common Name	Binding Affinity of the top ranked conformation (kcal/mol)[a]					
		PfHsp70-x-PFE0055c	HsHsp70-DNAJA1A	PfHsp70-2-PfSec63	HsGRP78-HsSec63	PFE005c J domain	DNAJA1 J domain
**2862146**	MBX 1641	**-6.6**	**-6.0**				
		-6.6	-6.0				
		-6.6	-5.8				
		-6.5	-6.0				
**55283**	Itraconazole			**-8.3**	**-7.5**		
				-8.1	-7.4		
				-8.3	-7.5		
				-8.3	-7.5		
**76685216**	Zoliflodacin			**-9.0**	**-8.1**		
				-9.0	-8.1		
				-9.0	-8.1		
				-9.0	-8.1		
**150311**	Ezetimibe					**-7.6**	**-5.7**
						-7.6	-7.0
						-7.6	-7.0
						-7.6	-5.6
**2867190**	Benzo-diazepinone					**-8.4**	**-6.6**
						-8.3	-6.6
						-8.4	-6.8
						-8.3	-6.6

[a]A total of three additional dockings were conducted for each compound to validate the consistency of the docking results. The top binders are indicated with bold font followed by the binding values for the three repeat dockings.The top binder from all the dockings gave a strong affinity and appropriate pose orientated at the interface and making contact with helix II of the J domain. The reproducibility of the binding pose was judged by comparing the binding affinity and conformation between the top binder and the repeat dockings. The repeat dockings results confirmed the consistency of the docking results with all the repeat docking is exhibitingthe same appropriate pose with a minor variation in the binding affinities.

Table S13: Details of binding affinity of the top scoring pose of all the compounds used for virtual screening against different Hsp70 cytosolic as well as ER complexes. The values are sorted based on the difference in binding affinities for PfHsp70-x-PFE0055c and HsHsp70-DNAJA1 complexes. Docking was conducted on NBD-J domain complexes.

**Ligand (PubchemCID)**	**Binding Affinity (kcal/mol)**						
	**PfHsp70-x-PFE0055c**	**PfHsp70-x-DNAJA1**	**HsHsp70-PFE0055c**	**HsHsp70-DNAJA1**	**PfHsp70-2-PfSec63**	**HsGRP78-HsSec63**	**Difference PfHsp70-x-PFE0055c and HsHsp70-DNAJA1**
**833990**	-6.7	-5.3	-6.2	-5.8	-6.5	-6.5	-0.9
**9819085**	-6.7	-6.2	-6.9	-5.8	-6.3	-7.2	-0.9
**7658**	-5	-4.8	-4.6	-4.2	-4.8	-5.1	-0.8
**10046204**	-6.6	-6.8	-7	-5.8	-6.9	-6.3	-0.8
**464205**	-5.4	-5.3	-5	-4.7	-5	-5.5	-0.7
**14570151**	-6.7	-5.7	-5.8	-6	-6	-6.4	-0.7
**60196939**	-7.2	-5.4	-6.9	-6.6	-7.3	-5.6	-0.6
**802112**	-6.5	-6.2	-6.2	-5.9	-6.1	-5.8	-0.6
**2862146**	-6.6	-5.5	-6.6	-6	-6.4	-6.5	-0.6
**104943**	-6.5	-5.8	-6.5	-5.9	-6.5	-6.2	-0.6
**71812237**	-5.6	-5.5	-6	-5	-5.8	-6.1	-0.6
**1684**	-6.8	-6.7	-6.9	-6.3	-7.1	-7.2	-0.5
**72015**	-5.8	-5.6	-6.3	-5.3	-6.2	-5.7	-0.5
**6435415**	-5	-4.9	-4.5	-4.5	-5.5	-5.3	-0.5
**6918837**	-6.6	-5.6	-6.4	-6.1	-6.2	-7.5	-0.5
**52912224**	-6.8	-6.5	-6.7	-6.3	-6.6	-6.8	-0.5
**135480631**	-6.1	-5.5	-6.3	-5.6	-5.8	-6	-0.5
**516119**	-6.8	-6.5	-6.9	-6.3	-6.9	-6.9	-0.5
**9119536**	-6.6	-6.8	-6.4	-6.1	-6.7	-6.6	-0.5
**50942319**	-6.8	-6.6	-7.3	-6.3	-6.9	-6	-0.5
**11998575**	-7	-6.9	-7.3	-6.6	-7.4	-7.4	-0.4
**25138227**	-6.2	-5.8	-6.3	-5.8	-6.1	-6.5	-0.4
**610864**	-6.7	-6.4	-6.5	-6.3	-6.5	-6.7	-0.4
**696714**	-6.7	-6.6	-6.8	-6.3	-6.8	-7	-0.4
**1486255**	-7	-6.8	-7.2	-6.6	-6.6	-7	-0.4
**9839083**	-5.4	-5	-5.2	-5	-5.6	-5.1	-0.4
**46916956**	-5.7	-5.8	-6	-5.3	-5.6	-5.8	-0.4
**11438772**	-6.5	-6.1	-6.9	-6.1	-6.8	-7.1	-0.4
**16744283**	-5.5	-5.8	-6.3	-5.1	-5.7	-6.4	-0.4
**3442**	-5.1	-5	-4.8	-4.7	-4.8	-5.2	-0.4
**4106**	-6.1	-6.3	-6.3	-5.7	-5.9	-5.1	-0.4
**57383474**	-7.3	-6.8	-7.3	-6.9	-7.1	-5.9	-0.4
**71525312**	-7.3	-6.7	-7.2	-6.9	-7	-7.4	-0.4

**75059312**	-6.1	-5.7	-6.7	-5.7	-6.5	-6.6	-0.4
**155541425**	-7.6	-6.9	-7.5	-7.2	-7.2	-7.7	-0.4
**503536**	-6.4	-6.1	-6.8	-6.1	-6	-6.3	-0.3
**344265**	-7.2	-6.7	-6.8	-6.9	-6.8	-7.3	-0.3
**3008319**	-6.7	-6.2	-6.6	-6.4	-6.6	-6.6	-0.3
**204104**	-7.2	-7.2	-7.9	-6.9	-7.4	-7	-0.3
**446244**	-6.3	-5.7	-6	-6	-6.5	-6.6	-0.3
**514840**	-5.5	-5.8	-6	-5.2	-6	-5.7	-0.3
**669397**	-6.2	-6.3	-6.1	-5.9	-6.1	-6.2	-0.3
**25053418**	-7.1	-8.3	-8.2	-6.8	-7.6	-7.7	-0.3
**155567195**	-7	-6.8	-7.2	-6.7	-6.8	-7.5	-0.3
**55262**	-5.5	-5.1	-5.7	-5.3	-6	-5.7	-0.2
**797337**	-6.3	-6.7	-7.3	-6.1	-6.5	-7.2	-0.2
**9911469**	-6.9	-6.6	-7.4	-6.7	-7.3	-8	-0.2
**24204260**	-6.8	-6.7	-7.2	-6.6	-6.8	-7.5	-0.2
**70685668**	-6.4	-5.1	-5.5	-6.2	-6	-6	-0.2
**124348**	-6.2	-6.6	-6.4	-6	-6.6	-6.4	-0.2
**259755**	-6.4	-6.5	-6.6	-6.2	-6.6	-6.8	-0.2
**309881**	-6.8	-6.3	-6.9	-6.6	-7	-6.9	-0.2
**2081089**	-6.7	-6.7	-6.7	-6.5	-6.5	-7.2	-0.2
**2997881**	-5.5	-5.3	-5.3	-5.3	-5.2	-5.2	-0.2
**56763769**	-4.2	-4	-4.3	-4	-4.3	-4.4	-0.2
**68388466**	-7.3	-6.5	-8	-7.1	-7.3	-8	-0.2
**1994681**	-6.6	-5.8	-6.8	-6.4	-6.6	-6.1	-0.2
**2768133**	-7.6	-7.2	-7.4	-7.4	-6.7	-7.1	-0.2
**14040421**	-7.1	-8.2	-7.9	-6.9	-7.7	-8.3	-0.2
**73305**	-4.6	-4.1	-4.2	-4.4	-4.6	-4.1	-0.2
**154234**	-5.6	-5.5	-5.4	-5.4	-5.5	-5.2	-0.2
**6478675**	-6.1	-6.3	-6.9	-5.9	-6.8	-6.7	-0.2
**65576**	-7.9	-7.1	-8.3	-7.8	-7.9	-8.2	-0.1
**742756**	-6.4	-6.5	-6.6	-6.3	-6.4	-6.3	-0.1
**5061639**	-5.9	-5.3	-6.4	-5.8	-5.7	-6	-0.1
**76336146**	-4.7	-4.4	-4.3	-4.6	-4.9	-4.7	-0.1
**118725992**	-6.9	-7.1	-7.7	-6.8	-7	-7.2	-0.1
**1455**	-6.4	-5.9	-7	-6.3	-6.4	-7	-0.1
**5583**	-6.4	-5.7	-6.7	-6.3	-6.7	-7.1	-0.1
**668392**	-5.2	-5.5	-5.4	-5.1	-5.2	-5.5	-0.1
**8392433**	-6.4	-5.7	-6.3	-6.3	-7.2	-6.2	-0.1
**155546288**	-6.9	-6.9	-7.2	-6.8	-7	-7.6	-0.1
**3998**	-4.8	-5	-4.7	-4.7	-4.9	-4.9	-0.1
**446987**	-3.8	-3.7	-3.6	-3.7	-4	-3.6	-0.1
**1549008**	-6.3	-5.7	-6.5	-6.2	-5.8	-6.3	-0.1
**2879322**	-5.8	-5.8	-5.8	-5.7	-5.3	-6.2	-0.1

**5480200**	-7.1	-6.8	-6.9	-7	-7.2	-7.2	-0.1
**5849412**	-4.5	-4.1	-4.3	-4.4	-4.4	-4.4	-0.1
**6622676**	-6.3	-5.7	-6.7	-6.2	-7.2	-7	-0.1
**10014426**	-6.3	-6.2	-6.5	-6.2	-6.6	-6.3	-0.1
**51354502**	-7.5	-6.9	-7.4	-7.4	-7.3	-7.5	-0.1
**54764565**	-8.1	-8.5	-8.3	-8	-7.7	-7.8	-0.1
**59191218**	-5.8	-5.3	-6	-5.7	-5.8	-5.7	-0.1
**894115**	-6.6	-5.9	-6.7	-6.5	-6.3	-6.3	-0.1
**2549475**	-6.8	-6.4	-7	-6.7	-6.7	-7.2	-0.1
**3047730**	-4.6	-4.5	-4.8	-4.5	-4.7	-4.6	-0.1
**25105722**	-6.3	-6.5	-6.4	-6.2	-6.5	-6.6	-0.1
**c86**	-5.5	-5.7	-6.2	-5.5	-5.9	-6.4	0
**14279**	-3.8	-3.5	-4	-3.8	-4.2	-4	0
**159326**	-6.9	-6.9	-7.3	-6.9	-6.9	-6.9	0
**476861**	-5.2	-5.8	-5.7	-5.2	-5.3	-6.5	0
**501640**	-4.7	-4.8	-4.6	-4.7	-4.9	-5	0
**1289417**	-6.2	-5.2	-6.4	-6.2	-5.8	-7.1	0
**2303200**	-6.9	-7.1	-6.8	-6.9	-6.6	-7	0
**2763159**	-5.1	-5.5	-5.4	-5.1	-5.6	-5.9	0
**3035850**	-6.8	-6.3	-7.4	-6.8	-6.7	-6.9	0
**3783668**	-7.4	-7.2	-7.2	-7.4	-7.1	-8	0
**10193032**	-6.4	-5.8	-6.5	-6.4	-6.6	-6.5	0
**10662981**	-4.8	-4.8	-5.5	-4.8	-6	-5.6	0
**11530089**	-6.4	-5.7	-6.5	-6.4	-6.5	-6.8	0
**24703802**	-6.3	-6.2	-6.2	-6.3	-6.1	-6.2	0
**24765332**	-6.4	-6.6	-6.9	-6.4	-6.6	-6.2	0
**45484770**	-6.5	-6.4	-7.4	-6.5	-6.3	-6.9	0
**52938427**	-6.8	-6.5	-7.1	-6.8	-6.9	-6.9	0
**53357475**	-6.6	-6.8	-7.2	-6.6	-6.8	-6.6	0
**54690727**	-6.6	-6.1	-7	-6.6	-6.6	-7.5	0
**91827688**	-6.6	-6.5	-6.8	-6.6	-7.3	-7.4	0
**135415565**	-6.1	-5.5	-5.9	-6.1	-5.9	-6.2	0
**135494268**	-6.7	-6.7	-7	-6.7	-6.7	-6.9	0
**135743630**	-5.9	-6.1	-6.3	-5.9	-6.3	-6.4	0
**135970754**	-5.9	-5.7	-6.6	-5.9	-6.2	-6.8	0
**71651**	-5	-5.1	-5.2	-5	-4.9	-5.2	0
**352078**	-5.2	-5.7	-5.3	-5.2	-5.6	-5.3	0
**3570131**	-6.5	-6.7	-7	-6.5	-6.6	-7	0
**14522747**	-5.6	-4.9	-5.5	-5.6	-5.9	-5.9	0
**16221816**	-5.9	-7.2	-6.5	-5.9	-6.4	-6.8	0
**45137165**	-5.6	-5.4	-5.3	-5.6	-5.9	-5.9	0
**2778238**	-5.8	-5.7	-6.4	-5.8	-5.8	-6.7	0

**72356**	-5.1	-5.1	-5.4	-5.1	-4.9	-5.3	0
**71549763**	-7.6	-7.2	-7.8	-7.6	-8.2	-7.6	0
**91933855**	-7.2	-6.7	-7.2	-7.2	-6.9	-7.1	0
**118797941**	-7.6	-7.8	-8.2	-7.6	-8.2	-8.2	0
**3767**	-4.4	-4.4	-4.4	-4.5	-4.8	-4.5	0.1
**3899**	-5.9	-6.8	-6.2	-6	-6.2	-6	0.1
**25429**	-5	-5.2	-5.5	-5.1	-5.6	-5.4	0.1
**47898**	-6.4	-6.2	-6.7	-6.5	-6.4	-6.7	0.1
**115358**	-6	-5.7	-6.5	-6.1	-6.5	-6.1	0.1
**504457**	-6.9	-6.2	-7.3	-7	-7.1	-7.9	0.1
**670851**	-5.7	-5.5	-6.1	-5.8	-5.7	-6.9	0.1
**5388906**	-6.4	-6.6	-7.5	-6.5	-6.3	-6.1	0.1
**11159021**	-6.2	-6.3	-6.3	-6.3	-6.2	-6.6	0.1
**11325072**	-7	-6.6	-7.2	-7.1	-7	-7.5	0.1
**11687907**	-6.2	-5.9	-6.5	-6.3	-6.5	-6.2	0.1
**25014868**	-5.7	-5.4	-5.7	-5.8	-6.1	-6	0.1
**44574267**	-6.2	-6.4	-6.9	-6.3	-6.3	-6.5	0.1
**46875261**	-5.4	-5.7	-5.2	-5.5	-5.6	-5.3	0.1
**56663065**	-5.4	-5.5	-5.7	-5.5	-5.7	-5.8	0.1
**135398513**	-5	-4.7	-4.8	-5.1	-5.7	-5.2	0.1
**2782**	-5.9	-6.2	-6.5	-6	-6.3	-6.6	0.1
**55245**	-6.4	-6.1	-7.1	-6.5	-6.7	-5.9	0.1
**82498**	-5.7	-5.9	-6.1	-5.8	-5.7	-6.3	0.1
**828705**	-6.2	-6	-6.6	-6.3	-6.5	-6.3	0.1
**2947925**	-6.9	-6.1	-6.8	-7	-6.3	-7.3	0.1
**44339407**	-6.4	-6.2	-7.4	-6.5	-6.4	-5.9	0.1
**135398739**	-5.2	-5	-5.4	-5.3	-5.3	-5.6	0.1
**352561**	-5.7	-5.9	-6.2	-5.8	-6	-6.1	0.1
**1046**	-3.9	-4	-3.9	-4	-4.5	-4.3	0.1
**54260**	-5.8	-6	-6.3	-5.9	-6.3	-6.6	0.1
**65781**	-6.1	-6.5	-7.1	-6.2	-7.1	-6.2	0.1
**71815**	-4.1	-4.1	-4.5	-4.2	-4.7	-4.4	0.1
**119607**	-6.1	-6.4	-6.5	-6.2	-6.5	-6.7	0.1
**5481990**	-7.3	-7.4	-8.6	-7.4	-6.8	-7.4	0.1
**9571836**	-4.6	-4.3	-4.8	-4.7	-5.3	-5.2	0.1
**70688612**	-5.3	-5.6	-5.8	-5.4	-5.9	-5.9	0.1
**135398740**	-5.1	-5.1	-5	-5.2	-5.9	-5.2	0.1
**10324367**	-5.8	-5.6	-6.8	-5.9	-7.4	-6.3	0.1
**25332**	-5.1	-5	-4.8	-5.2	-5	-4.7	0.1
**213043**	-6.3	-6.9	-6.8	-6.4	-6.3	-6.9	0.1
**423349**	-5.1	-4.9	-5.4	-5.2	-5.2	-5.4	0.1
**562945**	-5.8	-5.8	-6	-5.9	-6	-6.6	0.1
**2113979**	-5.8	-5.9	-6.9	-5.9	-6.1	-6.5	0.1

**998472**	-5.1	-5.2	-5.7	-5.2	-5.5	-5.6	0.1
**9882778**	-6.1	-6.1	-6.3	-6.2	-6.4	-6.6	0.1
**8593**	-6.4	-6.8	-6.6	-6.6	-6.5	-7	0.2
**4211702**	-6.4	-7.1	-6.8	-6.6	-7.1	-6.4	0.2
**6437877**	-5.9	-6.5	-6.4	-6.1	-6.3	-5.8	0.2
**71450388**	-7.4	-7.1	-7.7	-7.6	-7.9	-7.4	0.2
**50915187**	-6.9	-6.7	-6.8	-7.1	-7.4	-7.4	0.2
**10077130**	-7.5	-6.9	-8.5	-7.7	-7.4	-8	0.2
**5092**	-5.7	-6.4	-6.5	-5.9	-5.9	-6.1	0.2
**55283**	-7.5	-7.3	-8.8	-7.7	-8.3	-7.5	0.2
**100492**	-6.3	-6.6	-7.2	-6.5	-6.3	-7.2	0.2
**471161**	-6.1	-5.5	-6.2	-6.3	-6.1	-6.5	0.2
**656970**	-5.1	-5.3	-5.4	-5.3	-5.4	-5.4	0.2
**1371749**	-7	-7	-7.7	-7.2	-7.6	-7.3	0.2
**3003141**	-5	-5.6	-6	-5.2	-5.5	-5.2	0.2
**3607474**	-5.7	-5.4	-5.8	-5.9	-5.9	-6	0.2
**6077765**	-6	-5.7	-6.3	-6.2	-6.3	-6.3	0.2
**6918497**	-7.3	-7.6	-8.2	-7.5	-7.9	-7.3	0.2
**6992140**	-5.5	-6	-6.2	-5.7	-6.3	-6.6	0.2
**11703058**	-6	-6.5	-7	-6.2	-6.5	-6.7	0.2
**14870486**	-6.1	-6.5	-6.6	-6.3	-6.4	-6.9	0.2
**19165610**	-6.1	-5.9	-6.2	-6.3	-6.4	-7	0.2
**25164146**	-6.1	-5.5	-6.1	-6.3	-5.6	-5.8	0.2
**54579915**	-6	-6.4	-6.3	-6.2	-6.4	-6.9	0.2
**57331386**	-7	-7.6	-7.5	-7.2	-7.3	-7	0.2
**71574667**	-6.7	-6.5	-6.8	-6.9	-6.9	-7.2	0.2
**76328537**	-6.8	-7.1	-7.2	-7	-7.5	-7.4	0.2
**135413497**	-5.3	-6.1	-5.9	-5.5	-5.9	-5.7	0.2
**255968**	-6	-6.2	-6.5	-6.2	-6.4	-6.6	0.2
**2948477**	-6.1	-5.5	-5.5	-6.3	-5.6	-6.4	0.2
**5308364**	-6	-5.9	-6	-6.2	-5.9	-6.8	0.2
**9796068**	-4.8	-4.9	-5	-5	-4.9	-5.1	0.2
**10543588**	-7	-6.9	-7.2	-7.2	-7.5	-7.8	0.2
**53326415**	-7.7	-6.9	-8	-7.9	-7.4	-7	0.2
**68234645**	-6	-5.7	-6.4	-6.2	-6.3	-6.5	0.2
**11627742**	-6	-5.8	-6.4	-6.2	-6.1	-6.1	0.2
**16727326**	-7	-6.9	-7.9	-7.2	-7.1	-7.5	0.2
**135619737**	-7.3	-7.4	-7.7	-7.5	-7.5	-7.8	0.2
**15752801**	-5.3	-5.2	-5.7	-5.5	-5.8	-5.5	0.2
**60093290**	-5.7	-5.4	-6.1	-5.9	-5.8	-6.2	0.2
**491941**	-7	-6.7	-6.9	-7.2	-6.9	-7.1	0.2
**1055931**	-7.9	-7.3	-8.1	-8.2	-7.8	-8.2	0.3

**5426**	-5.7	-6.6	-6.7	-6	-6.4	-6.5	0.3
**3334**	-5.9	-5.6	-6.8	-6.2	-6.3	-6.6	0.3
**47576**	-6.9	-6.7	-7.9	-7.2	-7.5	-7.2	0.3
**161167**	-6.2	-5.9	-7.2	-6.5	-6.3	-6.5	0.3
**467825**	-6.2	-6.6	-6.6	-6.5	-7.3	-6.4	0.3
**470766**	-6.5	-6.7	-7.1	-6.8	-6.8	-6.1	0.3
**1380943**	-7.3	-7	-7.8	-7.6	-7.7	-7.8	0.3
**3190778**	-6.2	-6	-7.5	-6.5	-6.3	-6.8	0.3
**4638672**	-6.7	-7.2	-6.9	-7	-6.9	-7.2	0.3
**9850038**	-6	-7.1	-7	-6.3	-6.7	-6.8	0.3
**11712332**	-7.7	-7.4	-7.9	-8	-7.5	-7.6	0.3
**14007283**	-5.2	-5.2	-5.8	-5.5	-5.3	-5.5	0.3
**44355925**	-6	-6.5	-6.4	-6.3	-6.6	-6.4	0.3
**44416927**	-6.3	-6.3	-6.8	-6.6	-6.5	-6.7	0.3
**71492799**	-6.8	-7.3	-7.4	-7.1	-6.5	-7.8	0.3
**90675642**	-6.8	-6.9	-7	-7.1	-7.2	-7.2	0.3
**118502708**	-6.3	-6.3	-6.8	-6.6	-6.5	-6.6	0.3
**135409256**	-5.3	-5.1	-5.3	-5.6	-5.6	-5.9	0.3
**41684**	-5.3	-5.7	-5.9	-5.6	-6.4	-6.2	0.3
**256756**	-6.2	-6.7	-6.7	-6.5	-6.5	-7.2	0.3
**493482**	-6.5	-6.7	-7	-6.8	-6.9	-6.8	0.3
**514875**	-5.4	-6	-5.8	-5.7	-5.8	-6.4	0.3
**659069**	-6.7	-6.8	-7.1	-7	-6.9	-6.8	0.3
**2415319**	-5.8	-5.5	-6.1	-6.1	-6.1	-6.1	0.3
**25101148**	-5.9	-5.8	-6.2	-6.2	-6.2	-5.6	0.3
**27869350**	-6.5	-7	-7.3	-6.8	-6.8	-6.8	0.3
**352559**	-4	-4	-4.3	-4.3	-4.5	-4.2	0.3
**71506854**	-7.2	-7.6	-7.8	-7.5	-6.6	-7.6	0.3
**49846599**	-6.8	-6.4	-7.1	-7.1	-6.8	-7.1	0.3
**3366**	-3.9	-3.8	-4.1	-4.2	-4.4	-4.3	0.3
**3883**	-6.1	-5.7	-6.6	-6.4	-6.8	-6.5	0.3
**36294**	-5.6	-5.7	-5.7	-5.9	-5.7	-5.1	0.3
**5274428**	-5.1	-5.7	-5.8	-5.4	-5.7	-6.2	0.3
**9207941**	-5.6	-6.4	-6.3	-5.9	-5.9	-6.6	0.3
**10830128**	-6.6	-6.8	-7.3	-6.9	-7	-7.4	0.3
**44185380**	-6.1	-5.8	-6.8	-6.4	-6.3	-7.2	0.3
**4189**	-5.4	-6.2	-6	-5.8	-6.1	-6.1	0.4
**130313**	-4.7	-4.7	-5.2	-5.1	-5.3	-5.2	0.4
**441199**	-5.4	-5.6	-5.6	-5.8	-6.4	-5.9	0.4
**446598**	-5.2	-5.5	-5.9	-5.6	-5.4	-5	0.4
**452507**	-5.2	-6.3	-5.6	-5.6	-5.5	-6.1	0.4
**4165150**	-6.9	-6.8	-7.6	-7.3	-7.1	-7.4	0.4
**2749**	-5.2	-5.5	-6	-5.6	-6.1	-5.7	0.4

**160355**	-5.7	-5.8	-6.5	-6.1	-5.9	-6.4	0.4
**5376484**	-6.4	-5.9	-6.6	-6.8	-6.1	-6.6	0.4
**135410306**	-5.4	-5.4	-5.9	-5.8	-5.8	-5.7	0.4
**4858**	-6.1	-5.8	-6.6	-6.5	-7	-6.9	0.4
**5071**	-5.1	-5.6	-5.1	-5.5	-5.3	-5.4	0.4
**5578**	-5.3	-5.2	-5.8	-5.7	-5.4	-5.7	0.4
**6098**	-6.1	-6.3	-6.7	-6.5	-7.5	-6	0.4
**60700**	-6.6	-6.6	-8	-7	-6.6	-6.3	0.4
**3342298**	-6.8	-6.1	-7.3	-7.2	-7	-7.2	0.4
**11336042**	-5.1	-5	-5.4	-5.5	-5.7	-5.7	0.4
**12000240**	-7.1	-6.8	-6.5	-7.5	-7.4	-7.3	0.4
**52939192**	-6.1	-5.4	-6.8	-6.5	-6	-6.6	0.4
**82533**	-6.3	-6.5	-7.3	-6.7	-6.4	-6.7	0.4
**866407**	-5.6	-6.2	-6.1	-6	-6	-6.1	0.4
**2815587**	-5.3	-5.3	-5.6	-5.7	-5.4	-5.7	0.4
**4754542**	-5.5	-5.7	-6.4	-5.9	-6.3	-6.4	0.4
**9835049**	-4.6	-5	-5.2	-5	-5.3	-4.7	0.4
**45114297**	-7	-6.8	-8.2	-7.4	-7.2	-8.2	0.4
**137648729**	-7.5	-7.3	-7.7	-7.9	-8.1	-7.1	0.4
**2719**	-4.8	-5.4	-5.6	-5.3	-5.1	-5.6	0.5
**2484**	-5.6	-6.4	-6.3	-6.1	-6.8	-7	0.5
**14052**	-3.8	-3.9	-3.7	-4.3	-4.4	-4.2	0.5
**1570601**	-6.2	-6.1	-6.8	-6.7	-6.3	-6.5	0.5
**3421033**	-6.5	-6.5	-6.9	-7	-6.8	-6.9	0.5
**10088846**	-5	-5.1	-5.8	-5.5	-5.4	-5.7	0.5
**11573282**	-6	-6.1	-6.4	-6.5	-6.3	-6.9	0.5
**25101874**	-7.5	-7.3	-7.8	-8	-7.8	-7.5	0.5
**44563024**	-6.8	-7.8	-7.9	-7.3	-7.6	-8	0.5
**49856164**	-6.1	-6.5	-6.7	-6.6	-7	-6.6	0.5
**54726192**	-6.9	-7.2	-7.9	-7.4	-7.7	-7.1	0.5
**59208494**	-6.5	-6.5	-7.1	-7	-6.7	-7.4	0.5
**72708610**	-5.8	-6.1	-6.2	-6.3	-6.4	-6.6	0.5
**76310291**	-6.3	-7.6	-7.3	-6.8	-6.5	-6.3	0.5
**51634**	-4.4	-4.4	-4.7	-4.9	-5	-4.4	0.5
**130606**	-6.3	-6.6	-6.6	-6.8	-6.6	-6.8	0.5
**413628**	-8.1	-8.1	-9	-8.6	-8.9	-8.8	0.5
**657237**	-5	-5.4	-5.9	-5.5	-6.1	-6	0.5
**1038445**	-6.3	-6.4	-6.6	-6.8	-6.6	-6.5	0.5
**1317590**	-5.7	-6	-7.2	-6.2	-6.3	-6.8	0.5
**2083510**	-6.6	-7.2	-7.2	-7.1	-7.2	-6.8	0.5
**3013147**	-5.3	-5.6	-6.5	-5.8	-5.7	-6.7	0.5
**10523324**	-5.9	-6.6	-7.1	-6.4	-7	-7.2	0.5

**11375263**	-6.8	-6.4	-7.4	-7.3	-7.6	-7.6	0.5
**11485687**	-7.2	-7.4	-7.4	-7.7	-7.4	-6.7	0.5
**45105267**	-6.7	-6.8	-7.4	-7.2	-7	-7.1	0.5
**46178485**	-6.8	-7.1	-7.5	-7.3	-7.7	-7.5	0.5
**46886302**	-6.9	-7.3	-7.7	-7.4	-7.6	-7.7	0.5
**46891352**	-5.7	-5.9	-6.4	-6.2	-6.1	-6.4	0.5
**493469**	-5.8	-5.9	-6.4	-6.3	-6.4	-5.7	0.5
**66575905**	-6.4	-6.2	-7.1	-6.9	-7.3	-7.7	0.5
**71711529**	-6.6	-7	-7.1	-7.1	-6.8	-7.3	0.5
**53387035**	-7.8	-8	-7.6	-8.3	-8	-7.5	0.5
**3948**	-5.7	-6.5	-6.5	-6.3	-6.5	-6.7	0.6
**40854**	-5.7	-5.8	-6.4	-6.3	-6.5	-6.7	0.6
**214348**	-6.4	-7.2	-7.2	-7	-6.9	-7.6	0.6
**465951**	-5.7	-5.7	-6.4	-6.3	-6.4	-6.7	0.6
**5271819**	-5.2	-5.2	-5.9	-5.8	-5.7	-5.7	0.6
**44393579**	-5.4	-5.7	-6.3	-6	-5.8	-6.6	0.6
**67286591**	-7.2	-7.2	-7.1	-7.8	-7.4	-7.2	0.6
**76685216**	-8.3	-7.9	-9.7	-8.9	-9	-8.1	0.6
**461399**	-5.9	-6.2	-6.7	-6.5	-6.5	-6.3	0.6
**4252281**	-6.7	-7.2	-7.3	-7.3	-7.4	-7	0.6
**9972012**	-5.5	-5.5	-6.6	-6.1	-7.1	-6.8	0.6
**16739062**	-8.5	-8.2	-8.8	-9.1	-8.8	-8.4	0.6
**71481097**	-6.8	-7.6	-7.6	-7.4	-6.7	-8.1	0.6
**456199**	-6.1	-6.2	-6.6	-6.7	-6.7	-6.7	0.6
**472335**	-6.6	-6.7	-8.4	-7.2	-7.2	-6.8	0.6
**487718**	-5.8	-6.6	-6.1	-6.4	-6.3	-6.5	0.6
**493400**	-6.8	-6.1	-6.8	-7.4	-6.7	-7.3	0.6
**2941638**	-5.6	-5.6	-5.9	-6.2	-5.9	-5.8	0.6
**22305773**	-6.3	-7.2	-7.2	-6.9	-6.7	-6.5	0.6
**15997213**	-6.3	-7.1	-7	-6.9	-6.8	-6.7	0.6
**5288209**	-6.9	-7	-7.4	-7.6	-7.2	-7.6	0.7
**20443948**	-6.4	-7.1	-7.2	-7.1	-7	-6.9	0.7
**10027278**	-7.5	-7.5	-7.9	-8.2	-7.9	-7.5	0.7
**4544**	-5.7	-6.1	-6.7	-6.4	-6.9	-6.4	0.7
**64971**	-6.5	-6.9	-7.6	-7.2	-7.1	-6.3	0.7
**72051**	-6	-6.4	-6.8	-6.7	-6.4	-6.2	0.7
**24780598**	-6.5	-6.6	-7.6	-7.2	-7.1	-6.9	0.7
**44208849**	-5.8	-6	-6.6	-6.5	-6.3	-6.5	0.7
**56668933**	-6.5	-6.6	-6.8	-7.2	-7.4	-6.8	0.7
**71740433**	-5.5	-5.9	-5.8	-6.2	-6.1	-5.9	0.7
**2794**	-6.5	-6.7	-8.4	-7.2	-7	-6.9	0.7
**3365**	-5.1	-5.5	-6	-5.8	-5.7	-5.8	0.7
**150311**	-6	-5.8	-6.6	-6.7	-7.2	-7.4	0.7

**5189681**	-6.7	-6.8	-7.5	-7.4	-7.3	-6.2	0.7
**6918462**	-6.3	-5.9	-6.8	-7	-6.5	-6.6	0.7
**7019465**	-5.6	-5.4	-5.9	-6.3	-5.4	-6.4	0.7
**11385393**	-7	-8.2	-8.2	-7.7	-7.5	-7.7	0.7
**153274580**	-5.7	-5.3	-6.1	-6.4	-5.9	-6.1	0.7
**5291**	-7.4	-7.6	-8.5	-8.2	-7.9	-7.9	0.8
**71456558**	-7.9	-7.8	-8.9	-8.7	-8.3	-8.6	0.8
**4057**	-5.5	-6.1	-6.8	-6.3	-6.1	-6.3	0.8
**65015**	-6.5	-6.4	-7.3	-7.3	-7.5	-7.8	0.8
**3011893**	-5.4	-5.2	-5.8	-6.2	-5.3	-5.2	0.8
**135413525**	-5.4	-5.7	-6.1	-6.2	-6.5	-6.3	0.8
**1158563**	-6.3	-6.7	-7.3	-7.1	-7	-7.1	0.8
**11129373**	-6	-6.4	-6.5	-6.8	-6.4	-6.7	0.8
**11647548**	-6.2	-7.1	-7.1	-7	-6.8	-7.2	0.8
**45138674**	-6.3	-6.4	-7.2	-7.1	-7	-6.1	0.8
**135976563**	-6.9	-7.4	-7.6	-7.7	-7	-7.7	0.8
**21755317**	-4.9	-5.4	-6.1	-5.7	-5.7	-5	0.8
**9799894**	-5.9	-6.4	-6.5	-6.7	-6.5	-6.5	0.8
**10955174**	-5.2	-5.7	-5.7	-6	-5.7	-4.9	0.8
**2392**	-7.6	-7.6	-8.8	-8.4	-7.8	-8.4	0.8
**23730143**	-7.6	-8.2	-8.9	-8.4	-8.8	-8	0.8
**44143209**	-6.1	-6.1	-6.8	-6.9	-6.7	-6.8	0.8
**3011189**	-5.9	-6.1	-7.1	-6.8	-6.5	-7.1	0.9
**77050711**	-6.9	-6.8	-7.7	-7.8	-7.5	-7.2	0.9
**2804888**	-6.2	-6	-6.9	-7.1	-6.6	-6.4	0.9
**3712088**	-6.9	-7.1	-7.4	-7.8	-7	-7.1	0.9
**159603**	-5.6	-5.9	-5.6	-6.5	-5.8	-6.2	0.9
**1102158**	-5.5	-5.7	-6.4	-6.4	-6.2	-6.4	0.9
**16076226**	-6.3	-6.3	-8	-7.2	-7.9	-8	0.9
**44300725**	-6	-6.4	-7	-6.9	-6.5	-7.3	0.9
**68234908**	-7.4	-8.1	-8.4	-8.3	-8.1	-8.1	0.9
**3918849**	-6.5	-6.8	-7.9	-7.4	-7.4	-7.5	0.9
**60093267**	-6.5	-7.7	-7.4	-7.4	-7.4	-7.3	0.9
**3000226**	-6	-6.4	-7.2	-7	-6.3	-6.7	1
**11644726**	-4.3	-4.9	-5.1	-5.3	-5.3	-4.7	1
**71580391**	-5.2	-5.3	-5.8	-6.2	-6.1	-5.9	1
**86298528**	-6.4	-7.5	-7.2	-7.4	-6.7	-7.3	1
**2133850**	-6.1	-6.8	-7.6	-7.2	-7	-7.6	1.1
**2867190**	-6.8	-7.9	-8.3	-8	-7.9	-7.6	1.2
**49800090**	-6.2	-6.1	-7	-7.6	-6.8	-6.3	1.4
**91885568**	-7.2	-7.7	-8	-8.6	-8.3	-7.8	1.4

Table S14: Details of binding affinity of the top scoring pose of all the compounds used for virtual screening against PfHsp70-2-PfSec63 and HsGRP78-HsSec63 complexes. The values are sorted based on the difference in binding affinities for PfHsp70-2-PfSec63 and HsGRP78-HsSec63. Docking was conducted on NBD-J domain complexes.

**Ligand (PubchemCID)**	**Binding Affinity (kcal/mol)**		
	**PfHsp70-2-PfSec63**	**HsGRP78-HsSec63**	**Difference between PfHsp70-2-PfSec63 and HsGRP78-HsSec63**
**60196939**	-7.3	-5.6	-1.7
**6098**	-7.5	-6	-1.5
**57383474**	-7.1	-5.9	-1.2
**10324367**	-7.4	-6.3	-1.1
**5189681**	-7.3	-6.2	-1.1
**8392433**	-7.2	-6.2	-1
**137648729**	-8.1	-7.1	-1
**76685216**	-9	-8.1	-0.9
**45138674**	-7	-6.1	-0.9
**50942319**	-6.9	-6	-0.9
**65781**	-7.1	-6.2	-0.9
**467825**	-7.3	-6.4	-0.9
**4106**	-5.9	-5.1	-0.8
**55283**	-8.3	-7.5	-0.8
**23730143**	-8.8	-8	-0.8
**64971**	-7.1	-6.3	-0.8
**55245**	-6.7	-5.9	-0.8
**10955174**	-5.7	-4.9	-0.8
**470766**	-6.8	-6.1	-0.7
**135398740**	-5.9	-5.2	-0.7
**11485687**	-7.4	-6.7	-0.7
**493469**	-6.4	-5.7	-0.7
**21755317**	-5.7	-5	-0.7
**4211702**	-7.1	-6.4	-0.7
**36294**	-5.7	-5.1	-0.6
**6918497**	-7.9	-7.3	-0.6
**10046204**	-6.9	-6.3	-0.6
**54726192**	-7.7	-7.1	-0.6
**56668933**	-7.4	-6.8	-0.6
**25101148**	-6.2	-5.6	-0.6
**11644726**	-5.3	-4.7	-0.6
**51634**	-5	-4.4	-0.6
**9835049**	-5.3	-4.7	-0.6
**71549763**	-8.2	-7.6	-0.6

**91885568**	-8.3	-7.8	-0.5
**4544**	-6.9	-6.4	-0.5
**72015**	-6.2	-5.7	-0.5
**441199**	-6.4	-5.9	-0.5
**1994681**	-6.6	-6.1	-0.5
**6437877**	-6.3	-5.8	-0.5
**53387035**	-8	-7.5	-0.5
**71450388**	-7.9	-7.4	-0.5
**135398513**	-5.7	-5.2	-0.5
**73305**	-4.6	-4.1	-0.5
**44339407**	-6.4	-5.9	-0.5
**49800090**	-6.8	-6.3	-0.5
**9839083**	-5.6	-5.1	-0.5
**115358**	-6.5	-6.1	-0.4
**446598**	-5.4	-5	-0.4
**472335**	-7.2	-6.8	-0.4
**10662981**	-6	-5.6	-0.4
**49856164**	-7	-6.6	-0.4
**204104**	-7.4	-7	-0.4
**2083510**	-7.2	-6.8	-0.4
**4252281**	-7.4	-7	-0.4
**10027278**	-7.9	-7.5	-0.4
**16739062**	-8.8	-8.4	-0.4
**53326415**	-7.4	-7	-0.4
**446987**	-4	-3.6	-0.4
**24765332**	-6.6	-6.2	-0.4
**2749**	-6.1	-5.7	-0.4
**2867190**	-7.9	-7.6	-0.3
**3767**	-4.8	-4.5	-0.3
**3883**	-6.8	-6.5	-0.3
**47576**	-7.5	-7.2	-0.3
**55262**	-6	-5.7	-0.3
**60700**	-6.6	-6.3	-0.3
**71815**	-4.7	-4.4	-0.3
**802112**	-6.1	-5.8	-0.3
**1371749**	-7.6	-7.3	-0.3
**3003141**	-5.5	-5.2	-0.3
**10014426**	-6.6	-6.3	-0.3
**11687907**	-6.5	-6.2	-0.3
**25101874**	-7.8	-7.5	-0.3
**46875261**	-5.6	-5.3	-0.3
**57331386**	-7.3	-7	-0.3

**77050711**	-7.5	-7.2	-0.3
**25332**	-5	-4.7	-0.3
**104943**	-6.5	-6.2	-0.3
**154234**	-5.5	-5.2	-0.3
**352078**	-5.6	-5.3	-0.3
**514840**	-6	-5.7	-0.3
**9972012**	-7.1	-6.8	-0.3
**15752801**	-5.8	-5.5	-0.3
**352559**	-4.5	-4.2	-0.3
**1046**	-4.5	-4.3	-0.2
**3899**	-6.2	-6	-0.2
**14052**	-4.4	-4.2	-0.2
**14279**	-4.2	-4	-0.2
**72051**	-6.4	-6.2	-0.2
**5388906**	-6.3	-6.1	-0.2
**6435415**	-5.5	-5.3	-0.2
**6622676**	-7.2	-7	-0.2
**22305773**	-6.7	-6.5	-0.2
**53357475**	-6.8	-6.6	-0.2
**67286591**	-7.4	-7.2	-0.2
**76310291**	-6.5	-6.3	-0.2
**76336146**	-4.9	-4.7	-0.2
**135413497**	-5.9	-5.7	-0.2
**135413525**	-6.5	-6.3	-0.2
**41684**	-6.4	-6.2	-0.2
**461399**	-6.5	-6.3	-0.2
**828705**	-6.5	-6.3	-0.2
**46178485**	-7.7	-7.5	-0.2
**25429**	-5.6	-5.4	-0.2
**24780598**	-7.1	-6.9	-0.2
**44355925**	-6.6	-6.4	-0.2
**71580391**	-6.1	-5.9	-0.2
**71740433**	-6.1	-5.9	-0.2
**124348**	-6.6	-6.4	-0.2
**2804888**	-6.6	-6.4	-0.2
**742756**	-6.4	-6.3	-0.1
**2941638**	-5.9	-5.8	-0.1
**12000240**	-7.4	-7.3	-0.1
**3366**	-4.4	-4.3	-0.1
**493482**	-6.9	-6.8	-0.1
**659069**	-6.9	-6.8	-0.1
**3047730**	-4.7	-4.6	-0.1
**9119536**	-6.7	-6.6	-0.1

**60093267**	-7.4	-7.3	-0.1
**4858**	-7	-6.9	-0.1
**130313**	-5.3	-5.2	-0.1
**3011893**	-5.3	-5.2	-0.1
**9571836**	-5.3	-5.2	-0.1
**10193032**	-6.6	-6.5	-0.1
**20443948**	-7	-6.9	-0.1
**25014868**	-6.1	-6	-0.1
**59191218**	-5.8	-5.7	-0.1
**76328537**	-7.5	-7.4	-0.1
**2794**	-7	-6.9	-0.1
**309881**	-7	-6.9	-0.1
**413628**	-8.9	-8.8	-0.1
**657237**	-6.1	-6	-0.1
**1038445**	-6.6	-6.5	-0.1
**6478675**	-6.8	-6.7	-0.1
**15997213**	-6.8	-6.7	-0.1
**135410306**	-5.8	-5.7	-0.1
**5291**	-7.9	-7.9	0
**3998**	-4.9	-4.9	0
**4189**	-6.1	-6.1	0
**159326**	-6.9	-6.9	0
**456199**	-6.7	-6.7	0
**656970**	-5.4	-5.4	0
**3008319**	-6.6	-6.6	0
**5271819**	-5.7	-5.7	0
**5480200**	-7.2	-7.2	0
**5849412**	-4.4	-4.4	0
**6077765**	-6.3	-6.3	0
**11336042**	-5.7	-5.7	0
**11998575**	-7.4	-7.4	0
**52938427**	-6.9	-6.9	0
**68234908**	-8.1	-8.1	0
**70685668**	-6	-6	0
**70688612**	-5.9	-5.9	0
**90675642**	-7.2	-7.2	0
**516119**	-6.9	-6.9	0
**833990**	-6.5	-6.5	0
**894115**	-6.3	-6.3	0
**2415319**	-6.1	-6.1	0
**2997881**	-5.2	-5.2	0
**11375263**	-7.6	-7.6	0

**14522747**	-5.9	-5.9	0
**27869350**	-6.8	-6.8	0
**45137165**	-5.9	-5.9	0
**50915187**	-7.4	-7.4	0
**11627742**	-6.1	-6.1	0
**9799894**	-6.5	-6.5	0
**118797941**	-8.2	-8.2	0
**5426**	-6.4	-6.5	0.1
**501640**	-4.9	-5	0.1
**1380943**	-7.7	-7.8	0.1
**2862146**	-6.4	-6.5	0.1
**3607474**	-5.9	-6	0.1
**9850038**	-6.7	-6.8	0.1
**11712332**	-7.5	-7.6	0.1
**16076226**	-7.9	-8	0.1
**54764565**	-7.7	-7.8	0.1
**56663065**	-5.7	-5.8	0.1
**75059312**	-6.5	-6.6	0.1
**118502708**	-6.5	-6.6	0.1
**3365**	-5.7	-5.8	0.1
**446244**	-6.5	-6.6	0.1
**866407**	-6	-6.1	0.1
**1158563**	-7	-7.1	0.1
**3712088**	-7	-7.1	0.1
**3918849**	-7.4	-7.5	0.1
**6918462**	-6.5	-6.6	0.1
**25105722**	-6.5	-6.6	0.1
**44143209**	-6.7	-6.8	0.1
**45105267**	-7	-7.1	0.1
**998472**	-5.5	-5.6	0.1
**352561**	-6	-6.1	0.1
**1684**	-7.1	-7.2	0.1
**5071**	-5.3	-5.4	0.1
**3421033**	-6.8	-6.9	0.1
**24703802**	-6.1	-6.2	0.1
**91827688**	-7.3	-7.4	0.1
**135743630**	-6.3	-6.4	0.1
**669397**	-6.1	-6.2	0.1
**4754542**	-6.3	-6.4	0.1
**25053418**	-7.6	-7.7	0.1
**46886302**	-7.6	-7.7	0.1
**56763769**	-4.3	-4.4	0.1
**5092**	-5.9	-6.1	0.2

**72708610**	-6.4	-6.6	0.2
**255968**	-6.4	-6.6	0.2
**9796068**	-4.9	-5.1	0.2
**153274580**	-5.9	-6.1	0.2
**91933855**	-6.9	-7.1	0.2
**9882778**	-6.4	-6.6	0.2
**491941**	-6.9	-7.1	0.2
**2484**	-6.8	-7	0.2
**3948**	-6.5	-6.7	0.2
**4057**	-6.1	-6.3	0.2
**40854**	-6.5	-6.7	0.2
**119607**	-6.5	-6.7	0.2
**161167**	-6.3	-6.5	0.2
**487718**	-6.3	-6.5	0.2
**1102158**	-6.2	-6.4	0.2
**1570601**	-6.3	-6.5	0.2
**3035850**	-6.7	-6.9	0.2
**3342298**	-7	-7.2	0.2
**11703058**	-6.5	-6.7	0.2
**14007283**	-5.3	-5.5	0.2
**25164146**	-5.6	-5.8	0.2
**44208849**	-6.3	-6.5	0.2
**44416927**	-6.5	-6.7	0.2
**44574267**	-6.3	-6.5	0.2
**51354502**	-7.3	-7.5	0.2
**52912224**	-6.6	-6.8	0.2
**118725992**	-7	-7.2	0.2
**135480631**	-5.8	-6	0.2
**135494268**	-6.7	-6.9	0.2
**130606**	-6.6	-6.8	0.2
**150311**	-7.2	-7.4	0.2
**259755**	-6.6	-6.8	0.2
**423349**	-5.2	-5.4	0.2
**610864**	-6.5	-6.7	0.2
**696714**	-6.8	-7	0.2
**10523324**	-7	-7.2	0.2
**11385393**	-7.5	-7.7	0.2
**68234645**	-6.3	-6.5	0.2
**46916956**	-5.6	-5.8	0.2
**65576**	-7.9	-8.2	0.3
**71456558**	-8.3	-8.6	0.3
**3334**	-6.3	-6.6	0.3

**5578**	-5.4	-5.7	0.3
**7658**	-4.8	-5.1	0.3
**47898**	-6.4	-6.7	0.3
**54260**	-6.3	-6.6	0.3
**65015**	-7.5	-7.8	0.3
**465951**	-6.4	-6.7	0.3
**503536**	-6	-6.3	0.3
**4638672**	-6.9	-7.2	0.3
**5061639**	-5.7	-6	0.3
**6992140**	-6.3	-6.6	0.3
**10088846**	-5.4	-5.7	0.3
**11530089**	-6.5	-6.8	0.3
**71574667**	-6.9	-7.2	0.3
**135415565**	-5.9	-6.2	0.3
**2782**	-6.3	-6.6	0.3
**71651**	-4.9	-5.2	0.3
**82533**	-6.4	-6.7	0.3
**668392**	-5.2	-5.5	0.3
**2815587**	-5.4	-5.7	0.3
**10543588**	-7.5	-7.8	0.3
**11129373**	-6.4	-6.7	0.3
**135398739**	-5.3	-5.6	0.3
**135619737**	-7.5	-7.8	0.3
**11438772**	-6.8	-7.1	0.3
**49846599**	-6.8	-7.1	0.3
**71812237**	-5.8	-6.1	0.3
**2763159**	-5.6	-5.9	0.3
**4165150**	-7.1	-7.4	0.3
**135409256**	-5.6	-5.9	0.3
**46891352**	-6.1	-6.4	0.3
**2768133**	-6.7	-7.1	0.4
**5288209**	-7.2	-7.6	0.4
**11159021**	-6.2	-6.6	0.4
**5583**	-6.7	-7.1	0.4
**1055931**	-7.8	-8.2	0.4
**16221816**	-6.4	-6.8	0.4
**72356**	-4.9	-5.3	0.4
**3442**	-4.8	-5.2	0.4
**159603**	-5.8	-6.2	0.4
**471161**	-6.1	-6.5	0.4
**2303200**	-6.6	-7	0.4
**3000226**	-6.3	-6.7	0.4
**14570151**	-6	-6.4	0.4

**25138227**	-6.1	-6.5	0.4
**44563024**	-7.6	-8	0.4
**71525312**	-7	-7.4	0.4
**1486255**	-6.6	-7	0.4
**2113979**	-6.1	-6.5	0.4
**3570131**	-6.6	-7	0.4
**10830128**	-7	-7.4	0.4
**11647548**	-6.8	-7.2	0.4
**66575905**	-7.3	-7.7	0.4
**16727326**	-7.1	-7.5	0.4
**60093290**	-5.8	-6.2	0.4
**c86**	-5.9	-6.4	0.5
**2719**	-5.1	-5.6	0.5
**8593**	-6.5	-7	0.5
**344265**	-6.8	-7.3	0.5
**464205**	-5	-5.5	0.5
**1549008**	-5.8	-6.3	0.5
**3190778**	-6.3	-6.8	0.5
**5274428**	-5.7	-6.2	0.5
**11325072**	-7	-7.5	0.5
**14870486**	-6.4	-6.9	0.5
**54579915**	-6.4	-6.9	0.5
**160355**	-5.9	-6.4	0.5
**1317590**	-6.3	-6.8	0.5
**2549475**	-6.7	-7.2	0.5
**5376484**	-6.1	-6.6	0.5
**71711529**	-6.8	-7.3	0.5
**155541425**	-7.2	-7.7	0.5
**10077130**	-7.4	-8	0.6
**452507**	-5.5	-6.1	0.6
**493400**	-6.7	-7.3	0.6
**2133850**	-7	-7.6	0.6
**3011189**	-6.5	-7.1	0.6
**19165610**	-6.4	-7	0.6
**52939192**	-6	-6.6	0.6
**86298528**	-6.7	-7.3	0.6
**135970754**	-6.2	-6.8	0.6
**1455**	-6.4	-7	0.6
**82498**	-5.7	-6.3	0.6
**562945**	-6	-6.6	0.6
**155546288**	-7	-7.6	0.6
**2392**	-7.8	-8.4	0.6

**5481990**	-6.8	-7.4	0.6
**11573282**	-6.3	-6.9	0.6
**14040421**	-7.7	-8.3	0.6
**45484770**	-6.3	-6.9	0.6
**213043**	-6.3	-6.9	0.6
**514875**	-5.8	-6.4	0.6
**214348**	-6.9	-7.6	0.7
**9207941**	-5.9	-6.6	0.7
**797337**	-6.5	-7.2	0.7
**9911469**	-7.3	-8	0.7
**24204260**	-6.8	-7.5	0.7
**59208494**	-6.7	-7.4	0.7
**256756**	-6.5	-7.2	0.7
**2081089**	-6.5	-7.2	0.7
**135976563**	-7	-7.7	0.7
**68388466**	-7.3	-8	0.7
**155567195**	-6.8	-7.5	0.7
**16744283**	-5.7	-6.4	0.7
**44300725**	-6.5	-7.3	0.8
**44393579**	-5.8	-6.6	0.8
**504457**	-7.1	-7.9	0.8
**2948477**	-5.6	-6.4	0.8
**5308364**	-5.9	-6.8	0.9
**100492**	-6.3	-7.2	0.9
**2879322**	-5.3	-6.2	0.9
**3783668**	-7.1	-8	0.9
**54690727**	-6.6	-7.5	0.9
**9819085**	-6.3	-7.2	0.9
**44185380**	-6.3	-7.2	0.9
**2778238**	-5.8	-6.7	0.9
**45114297**	-7.2	-8.2	1
**2947925**	-6.3	-7.3	1
**3013147**	-5.7	-6.7	1
**7019465**	-5.4	-6.4	1
**71506854**	-6.6	-7.6	1
**476861**	-5.3	-6.5	1.2
**670851**	-5.7	-6.9	1.2
**1289417**	-5.8	-7.1	1.3
**6918837**	-6.2	-7.5	1.3
**71492799**	-6.5	-7.8	1.3
**71481097**	-6.7	-8.1	1.4

Table S15: Details of binding affinity of the top scoring pose of all the compounds used for virtual screening against various cytosolic and ER-specific J-domains. The values are sorted based on the difference in binding affinities for PFE0055c and DNAJA1. Docking was conducted using the J domains.

**Ligand (Pubchem CID)**	**Binding Affinity (kcal/mol)**				
	**PFE0055c**	**DNAJA1**	**PfSec63**	**HsSec63**	**Difference between PFE0055c and DNAJA1**
**150311**	-7.6	-5.7	-6.1	-5	-1.9
**2867190**	-8.4	-6.6	-7.3	-6.5	-1.8
**8392433**	-7	-5.3	-5.3	-5.8	-1.7
**1684**	-6.8	-5.3	-6.1	-5.4	-1.5
**155546288**	-7.6	-6.1	-6.5	-5.9	-1.5
**10027278**	-8	-6.6	-6.7	-6.1	-1.4
**2768133**	-7.6	-6.2	-6	-6	-1.4
**76310291**	-7.6	-6.2	-5.8	-5.9	-1.4
**115358**	-6.4	-5.1	-5.5	-5.2	-1.3
**3783668**	-7.7	-6.4	-6.9	-6.3	-1.3
**51354502**	-8	-6.7	-6.9	-6.6	-1.3
**71450388**	-8	-6.7	-6.2	-6	-1.3
**55245**	-7.3	-6	-6.3	-5.6	-1.3
**1158563**	-7.5	-6.2	-6	-6.9	-1.3
**16739062**	-9	-7.7	-7.9	-7.5	-1.3
**155541425**	-7.8	-6.5	-6.7	-6.7	-1.3
**10077130**	-7.9	-6.7	-6.8	-6	-1.2
**2763159**	-6	-4.8	-5.4	-5	-1.2
**56668933**	-7.3	-6.1	-6.1	-5.8	-1.2
**1317590**	-7	-5.8	-6	-5.2	-1.2
**9911469**	-7.1	-5.9	-6.6	-5.5	-1.2
**11712332**	-7.9	-6.8	-6.3	-6.1	-1.1
**487718**	-6.8	-5.7	-5.5	-5.7	-1.1
**1371749**	-7.6	-6.5	-6.6	-6.6	-1.1
**11530089**	-6.8	-5.7	-5.4	-5.6	-1.1
**1038445**	-7	-5.9	-6.3	-6.4	-1.1
**11129373**	-7	-5.9	-6	-5.7	-1.1
**49800090**	-7.1	-6	-6.1	-6.1	-1.1
**4544**	-6.4	-5.4	-5.4	-5.4	-1
**65576**	-7.8	-6.8	-6.6	-7.2	-1
**1289417**	-5.6	-4.6	-5.2	-4.7	-1
**2879322**	-5.9	-4.9	-5.4	-4.4	-1
**11325072**	-7.2	-6.2	-6.9	-5.7	-1
**11687907**	-6.8	-5.8	-6.3	-5.6	-1

**11998575**	-7.3	-6.3	-6.1	-6.1	-1
**14040421**	-8.1	-7.1	-6.7	-6.7	-1
**24204260**	-7.1	-6.1	-6.1	-5.3	-1
**44393579**	-6.1	-5.1	-5.6	-4.5	-1
**118502708**	-6.8	-5.8	-6	-5.7	-1
**135494268**	-7.3	-6.3	-6.8	-6.7	-1
**256756**	-7.2	-6.2	-5.9	-5.5	-1
**493482**	-6.8	-5.8	-6.2	-6.3	-1
**696714**	-6.9	-5.9	-5.8	-5.8	-1
**5189681**	-7.4	-6.4	-6.7	-5.7	-1
**5308364**	-6.5	-5.5	-5.6	-6	-1
**2778238**	-6.2	-5.2	-5.2	-5.2	-1
**9839083**	-5.4	-4.4	-4.6	-4.5	-1
**71506854**	-7.7	-6.7	-7.1	-6.4	-1
**60093290**	-6	-5	-5.2	-5.2	-1
**5291**	-8.2	-7.2	-7.4	-6.9	-1
**2303200**	-7.2	-6.3	-6.5	-6.6	-0.9
**4638672**	-7.2	-6.3	-6.5	-6.5	-0.9
**57331386**	-7	-6.1	-6.6	-5.8	-0.9
**77050711**	-7.2	-6.3	-6.6	-6.2	-0.9
**91827688**	-6.7	-5.8	-5.7	-6	-0.9
**204104**	-7.4	-6.5	-6.5	-5.8	-0.9
**2549475**	-7	-6.1	-6.3	-6.1	-0.9
**10830128**	-7.4	-6.5	-6.6	-6.2	-0.9
**25101874**	-7.6	-6.7	-6.9	-6.5	-0.9
**45484770**	-7.1	-6.2	-6.7	-5.8	-0.9
**71580391**	-6.3	-5.4	-5.6	-4.6	-0.9
**118725992**	-7.6	-6.7	-6.8	-6.4	-0.9
**461399**	-6.8	-5.9	-5.7	-5.3	-0.9
**562945**	-6.3	-5.4	-5.3	-5.5	-0.9
**2804888**	-6.6	-5.7	-5.7	-5.7	-0.9
**3570131**	-7.1	-6.2	-6	-5.5	-0.9
**10523324**	-7.1	-6.2	-6.4	-5.7	-0.9
**45114297**	-7.8	-6.9	-6.8	-6.2	-0.9
**504457**	-6.9	-6.1	-6.2	-5.8	-0.8
**57383474**	-6.9	-6.1	-6	-5.5	-0.8
**352561**	-6.4	-5.6	-5.7	-5.6	-0.8
**65015**	-6.6	-5.8	-5.6	-5.3	-0.8
**476861**	-5.8	-5	-4.5	-5.2	-0.8
**11703058**	-6.7	-5.9	-6.2	-5.6	-0.8
**24703802**	-6.5	-5.7	-5.5	-5.6	-0.8
**60196939**	-6.2	-5.4	-5.1	-6.4	-0.8
**71525312**	-7.1	-6.3	-6.5	-5.8	-0.8

**82533**	-7.1	-6.3	-6.6	-5.6	-0.8
**124348**	-6.8	-6	-5.5	-5.9	-0.8
**259755**	-7.1	-6.3	-5.9	-6.3	-0.8
**828705**	-6.5	-5.7	-5.7	-5.8	-0.8
**16221816**	-7.1	-6.3	-5.3	-5.6	-0.8
**44339407**	-6.7	-5.9	-6.2	-5.1	-0.8
**72356**	-5.2	-4.4	-4.8	-4.5	-0.8
**91933855**	-7.3	-6.5	-6.2	-6.7	-0.8
**c86**	-6	-5.3	-5.3	-5.3	-0.7
**71481097**	-7.3	-6.6	-6.5	-6.3	-0.7
**3334**	-6.4	-5.7	-5.6	-6.2	-0.7
**3899**	-6.5	-5.8	-5.4	-5.4	-0.7
**467825**	-6.8	-6.1	-6	-5.6	-0.7
**670851**	-6.3	-5.6	-5.2	-5.4	-0.7
**797337**	-6.2	-5.5	-6	-5.3	-0.7
**1570601**	-6.5	-5.8	-6.1	-6	-0.7
**2133850**	-6.8	-6.1	-6.8	-5.8	-0.7
**5480200**	-7.2	-6.5	-6.2	-5.9	-0.7
**25138227**	-6.4	-5.7	-5.5	-4.9	-0.7
**25164146**	-5.7	-5	-5.3	-5.2	-0.7
**54726192**	-7.2	-6.5	-6.2	-6.3	-0.7
**1455**	-6.5	-5.8	-6.1	-5.6	-0.7
**130606**	-6.5	-5.8	-5.5	-5.5	-0.7
**309881**	-7	-6.3	-6.3	-6.3	-0.7
**894115**	-6.4	-5.7	-5.9	-5.5	-0.7
**2947925**	-6.5	-5.8	-6	-5.9	-0.7
**5376484**	-6.2	-5.5	-5.6	-5.7	-0.7
**11485687**	-7.2	-6.5	-6.9	-6.7	-0.7
**45105267**	-6.7	-6	-6.3	-6	-0.7
**46178485**	-7.5	-6.8	-6.6	-6.3	-0.7
**46891352**	-6.4	-5.7	-5.4	-4.8	-0.7
**71549763**	-7.7	-7	-6.9	-6.7	-0.7
**9799894**	-7	-6.3	-6	-5.6	-0.7
**3607474**	-6.1	-5.4	-5.4	-5.3	-0.7
**5288209**	-7.1	-6.4	-6.5	-5.9	-0.7
**2815587**	-5.6	-4.9	-5	-4.8	-0.7
**7019465**	-5.6	-4.9	-5.7	-4.8	-0.7
**68388466**	-7.1	-6.4	-7.3	-6.3	-0.7
**71711529**	-7.1	-6.4	-6.1	-5.8	-0.7
**5426**	-6.7	-6.1	-6	-5.7	-0.6
**16076226**	-6.9	-6.3	-6.4	-6.6	-0.6
**24765332**	-6.9	-6.3	-6.2	-5.2	-0.6

**44416927**	-6.9	-6.3	-6.5	-5.7	-0.6
**52938427**	-6.9	-6.3	-6	-5.9	-0.6
**71740433**	-5.9	-5.3	-5	-5	-0.6
**1486255**	-6.7	-6.1	-6.9	-5.7	-0.6
**11385393**	-7.9	-7.3	-6.8	-6	-0.6
**135619737**	-7.7	-7.1	-7	-6.3	-0.6
**15752801**	-5.9	-5.3	-5.4	-5	-0.6
**71812237**	-5.7	-5.1	-5.4	-5.5	-0.6
**491941**	-6.9	-6.3	-6	-6.5	-0.6
**3948**	-6.3	-5.7	-5.6	-5.5	-0.6
**4057**	-6.1	-5.5	-5.9	-5	-0.6
**47576**	-7.1	-6.5	-6.3	-5.9	-0.6
**470766**	-7.3	-6.7	-6.6	-6.4	-0.6
**471161**	-5.5	-4.9	-6.1	-4.4	-0.6
**9850038**	-6.6	-6	-5.7	-4.9	-0.6
**11336042**	-5.1	-4.5	-4.8	-4.6	-0.6
**14870486**	-6.6	-6	-6	-6.4	-0.6
**22305773**	-6.6	-6	-6.1	-5.6	-0.6
**44208849**	-6.6	-6	-5.5	-5.6	-0.6
**44355925**	-6	-5.4	-5.7	-5.4	-0.6
**70685668**	-6.6	-6	-4.8	-5.3	-0.6
**70688612**	-6	-5.4	-5.1	-5.2	-0.6
**72708610**	-6.3	-5.7	-5.2	-5	-0.6
**76328537**	-7.5	-6.9	-6.6	-5.9	-0.6
**5583**	-6.3	-5.7	-5.5	-5.2	-0.6
**3013147**	-5.6	-5	-5.2	-5.1	-0.6
**11375263**	-6.8	-6.2	-6.8	-5.9	-0.6
**27869350**	-6.8	-6.2	-6.9	-5.7	-0.6
**46916956**	-5.6	-5	-5.4	-5.4	-0.6
**11438772**	-7.1	-6.5	-5.8	-5.7	-0.6
**2719**	-5.3	-4.8	-4.5	-4.6	-0.5
**2484**	-6.6	-6.1	-6.1	-5.5	-0.5
**3883**	-6.5	-6	-5.9	-5.2	-0.5
**3998**	-4.7	-4.2	-4.3	-4.2	-0.5
**72015**	-5.4	-4.9	-5.2	-4.7	-0.5
**119607**	-6.2	-5.7	-5.8	-5.5	-0.5
**159603**	-5.6	-5.1	-5.5	-4.8	-0.5
**742756**	-6.3	-5.8	-6.1	-5.9	-0.5
**802112**	-6.4	-5.9	-5.9	-5.6	-0.5
**1994681**	-6.3	-5.8	-5.7	-5	-0.5
**2862146**	-6.2	-5.7	-6	-5.3	-0.5
**3342298**	-6.7	-6.2	-6.5	-5.6	-0.5
**4165150**	-7.4	-6.9	-6.3	-5.9	-0.5

**5271819**	-5.4	-4.9	-5	-4.7	-0.5
**5274428**	-5.9	-5.4	-4.8	-5.1	-0.5
**6077765**	-6.1	-5.6	-5.8	-5.9	-0.5
**10088846**	-5.4	-4.9	-5.8	-4.6	-0.5
**10193032**	-6.4	-5.9	-5.9	-5.8	-0.5
**71574667**	-6.9	-6.4	-6.3	-5.9	-0.5
**135398740**	-4.8	-4.3	-5	-4.4	-0.5
**71651**	-5.2	-4.7	-4.8	-5	-0.5
**514840**	-5.6	-5.1	-5.1	-4.9	-0.5
**514875**	-5.7	-5.2	-5.1	-4.7	-0.5
**669397**	-6.2	-5.7	-5.3	-5.4	-0.5
**3712088**	-6.9	-6.4	-7	-6.3	-0.5
**15997213**	-6.5	-6	-6.1	-5.6	-0.5
**25053418**	-7.7	-7.2	-7.3	-5	-0.5
**25101148**	-6.1	-5.6	-5.4	-5.3	-0.5
**45137165**	-5.5	-5	-5.1	-5.4	-0.5
**50915187**	-7.2	-6.7	-6.2	-5.6	-0.5
**153274580**	-5.8	-5.3	-5.8	-4.4	-0.5
**9882778**	-6.3	-5.8	-5.3	-5.6	-0.5
**155567195**	-7.2	-6.7	-6.2	-5.9	-0.5
**4858**	-6.5	-6.1	-5.8	-5.7	-0.4
**54260**	-6.2	-5.8	-5.3	-5.9	-0.4
**344265**	-6.7	-6.3	-6.2	-6.1	-0.4
**472335**	-6.9	-6.5	-7.1	-6.1	-0.4
**1380943**	-7.5	-7.1	-6.6	-6.6	-0.4
**3003141**	-5.4	-5	-4.9	-4.5	-0.4
**6622676**	-6.7	-6.3	-6.2	-5.9	-0.4
**11159021**	-6.4	-6	-5.9	-5.3	-0.4
**14007283**	-5.7	-5.3	-5.3	-5	-0.4
**49856164**	-6.2	-5.8	-5.8	-6.5	-0.4
**54579915**	-6.4	-6	-5.2	-5.9	-0.4
**2794**	-6.7	-6.3	-6.5	-5.9	-0.4
**3365**	-5.7	-5.3	-5.2	-4.7	-0.4
**82498**	-6	-5.6	-5.3	-5.5	-0.4
**2083510**	-6.5	-6.1	-6.1	-6.2	-0.4
**25105722**	-6.4	-6	-5.7	-5.6	-0.4
**16727326**	-6.5	-6.1	-6.8	-5.6	-0.4
**137648729**	-7.5	-7.1	-7	-6.5	-0.4
**118797941**	-8	-7.6	-6.9	-6.7	-0.4
**7658**	-5.1	-4.7	-4.1	-4.4	-0.4
**47898**	-6.8	-6.4	-5.6	-5.6	-0.4
**503536**	-6.3	-5.9	-5.6	-5.3	-0.4

**2941638**	-6.1	-5.7	-5.4	-5.3	-0.4
**3000226**	-5.8	-5.4	-5.3	-5.9	-0.4
**3011189**	-6.6	-6.2	-5.7	-5.8	-0.4
**3035850**	-6.6	-6.2	-6.1	-5.7	-0.4
**3190778**	-6.1	-5.7	-5.6	-5.4	-0.4
**5388906**	-6.6	-6.2	-6.2	-5.5	-0.4
**5481990**	-7.8	-7.4	-5.7	-6.5	-0.4
**11644726**	-4.8	-4.4	-4.6	-4.4	-0.4
**75059312**	-6.3	-5.9	-5.9	-5.7	-0.4
**2782**	-6.1	-5.7	-5.5	-5.3	-0.4
**446244**	-6.1	-5.7	-5.7	-5.4	-0.4
**46886302**	-7.3	-6.9	-7.2	-5.9	-0.4
**11627742**	-5.8	-5.4	-5.4	-5.2	-0.4
**21755317**	-5.6	-5.2	-4.8	-4.4	-0.4
**501640**	-4.9	-4.6	-5.2	-4.1	-0.3
**54690727**	-6.4	-6.1	-6.2	-6.2	-0.3
**423349**	-4.9	-4.6	-4.6	-4.9	-0.3
**4189**	-6.2	-5.9	-5.6	-5.1	-0.3
**60700**	-6.5	-6.2	-6.7	-6.1	-0.3
**452507**	-5.2	-4.9	-5.2	-4.8	-0.3
**456199**	-6.5	-6.2	-5.6	-6	-0.3
**6918497**	-7.1	-6.8	-6.9	-6	-0.3
**10014426**	-6.3	-6	-5.6	-5.8	-0.3
**10046204**	-6.7	-6.4	-6	-4.8	-0.3
**10662981**	-5.1	-4.8	-5.4	-4.6	-0.3
**52912224**	-6.1	-5.8	-6	-5.4	-0.3
**54764565**	-7.5	-7.2	-7.8	-6.2	-0.3
**56663065**	-6.1	-5.8	-5.4	-5.1	-0.3
**59191218**	-5.5	-5.2	-5	-4.6	-0.3
**68234908**	-7.8	-7.5	-7.1	-7	-0.3
**76685216**	-7.6	-7.3	-7.4	-6.8	-0.3
**90675642**	-6.7	-6.4	-6.3	-6.1	-0.3
**213043**	-5.8	-5.5	-5.8	-5.5	-0.3
**610864**	-6.2	-5.9	-5.9	-5.7	-0.3
**2113979**	-5.8	-5.5	-5.6	-4.8	-0.3
**2997881**	-5.2	-4.9	-5.1	-4.8	-0.3
**3918849**	-6.8	-6.5	-6.4	-5.9	-0.3
**4252281**	-7.2	-6.9	-6.3	-6.8	-0.3
**9119536**	-6.3	-6	-5.9	-6	-0.3
**9796068**	-4.7	-4.4	-4.7	-4.2	-0.3
**493469**	-5.8	-5.5	-5.7	-5.5	-0.3
**1046**	-3.7	-3.5	-4.4	-3.9	-0.2
**4106**	-5.8	-5.6	-5.3	-4.9	-0.2

**5092**	-5.9	-5.7	-5.1	-5.1	-0.2
**5578**	-5.3	-5.1	-4.9	-5	-0.2
**40854**	-6.2	-6	-5.7	-5	-0.2
**159326**	-6.9	-6.7	-6.6	-5.6	-0.2
**161167**	-6.4	-6.2	-6.5	-5.4	-0.2
**1102158**	-5.8	-5.6	-5.1	-4.8	-0.2
**4211702**	-6.8	-6.6	-6.2	-5.9	-0.2
**5849412**	-4.5	-4.3	-4.2	-3.7	-0.2
**6437877**	-5.7	-5.5	-5.5	-5.4	-0.2
**24780598**	-6.8	-6.6	-5.9	-6.8	-0.2
**59208494**	-6.5	-6.3	-6.1	-5.5	-0.2
**67286591**	-6.9	-6.7	-6.6	-5.9	-0.2
**135409256**	-5	-4.8	-5.5	-4.4	-0.2
**51634**	-4.4	-4.2	-5	-3.7	-0.2
**160355**	-5.8	-5.6	-5.8	-5.6	-0.2
**516119**	-6.3	-6.1	-5.7	-5.5	-0.2
**668392**	-5.3	-5.1	-5	-4.7	-0.2
**6478675**	-6	-5.8	-5.5	-5.3	-0.2
**9819085**	-6.2	-6	-5.9	-5.4	-0.2
**135398739**	-5	-4.8	-5.3	-4.9	-0.2
**71815**	-3.9	-3.7	-4.3	-3.9	-0.2
**55262**	-5.1	-4.9	-5.7	-4.9	-0.2
**46875261**	-5.1	-4.9	-5.7	-4.9	-0.2
**53357475**	-6.6	-6.4	-6.1	-5.3	-0.2
**6435415**	-4.9	-4.8	-4.9	-4.7	-0.1
**19165610**	-6.4	-6.3	-5.9	-5.9	-0.1
**25014868**	-5.9	-5.8	-5	-5.4	-0.1
**135415565**	-5.4	-5.3	-5.8	-5.1	-0.1
**255968**	-5.9	-5.8	-5.6	-5.4	-0.1
**9972012**	-5.9	-5.8	-5.8	-5.6	-0.1
**68234645**	-5.9	-5.8	-5.9	-5.3	-0.1
**49846599**	-6.4	-6.3	-5.6	-5.7	-0.1
**16744283**	-6.2	-6.1	-5.7	-6.1	-0.1
**352559**	-3.9	-3.8	-4.3	-4	-0.1
**3442**	-4.8	-4.7	-4.6	-4.3	-0.1
**656970**	-5.3	-5.2	-5.1	-4.6	-0.1
**3421033**	-7	-6.9	-6.4	-5.9	-0.1
**11573282**	-6.5	-6.4	-5.6	-5.8	-0.1
**135398513**	-4.3	-4.2	-4.9	-4.1	-0.1
**135743630**	-5.6	-5.5	-5.4	-5.8	-0.1
**104943**	-5.8	-5.7	-5.7	-4.8	-0.1
**154234**	-5.1	-5	-4.9	-4.8	-0.1

**657237**	-5.1	-5	-5.8	-4.9	-0.1
**866407**	-5.8	-5.7	-5.2	-4.9	-0.1
**1055931**	-6.8	-6.7	-6.3	-6.1	-0.1
**9207941**	-5.6	-5.5	-5.3	-5.3	-0.1
**50942319**	-6.1	-6	-5.8	-6	-0.1
**60093267**	-7.1	-7	-6.2	-6.1	-0.1
**3767**	-4.1	-4.1	-4.3	-4	0
**8593**	-6.7	-6.7	-6.3	-6.2	0
**14279**	-3.6	-3.6	-4.2	-3.5	0
**55283**	-7.3	-7.3	-6.4	-5.9	0
**441199**	-5.2	-5.2	-5.3	-4.8	0
**5061639**	-5.6	-5.6	-5.6	-5.9	0
**6918837**	-5.4	-5.4	-5.1	-4.5	0
**6992140**	-5.4	-5.4	-6	-4.9	0
**9571836**	-4.5	-4.5	-4.7	-4.2	0
**44300725**	-6.1	-6.1	-5.9	-5.7	0
**44574267**	-5.9	-5.9	-6.1	-5.4	0
**52939192**	-5.8	-5.8	-5.4	-5.4	0
**71492799**	-7	-7	-6.9	-5.3	0
**135970754**	-5.7	-5.7	-5.6	-4.9	0
**41684**	-5.6	-5.6	-5.8	-5.6	0
**659069**	-6.6	-6.6	-6	-5.8	0
**2081089**	-6.7	-6.7	-6.7	-6.2	0
**14522747**	-5.4	-5.4	-5.6	-4.7	0
**44143209**	-6.7	-6.7	-5.8	-6.2	0
**45138674**	-6.1	-6.1	-5.8	-5.5	0
**998472**	-5.1	-5.1	-5.1	-4.5	0
**14052**	-4	-4.1	-3.6	-3.7	0.1
**64971**	-6.5	-6.6	-6	-5.8	0.1
**72051**	-6.4	-6.5	-5.4	-5.3	0.1
**3011893**	-5	-5.1	-5.5	-4.6	0.1
**20443948**	-7.2	-7.3	-6.1	-5.8	0.1
**23730143**	-7.9	-8	-7.1	-7	0.1
**135413497**	-5.4	-5.5	-5.6	-4.9	0.1
**135480631**	-5.4	-5.5	-5.8	-5	0.1
**413628**	-7.5	-7.6	-8	-7.1	0.1
**833990**	-5.5	-5.6	-5.6	-4.8	0.1
**4754542**	-5.9	-6	-5.4	-5	0.1
**53326415**	-7.2	-7.3	-7.3	-6	0.1
**135410306**	-5.5	-5.6	-5.2	-4.9	0.1
**446987**	-3.4	-3.5	-4.1	-3.5	0.1
**214348**	-6.6	-6.7	-6.4	-6.1	0.1
**1549008**	-5.8	-5.9	-5.5	-5.3	0.1

**53387035**	-7.3	-7.4	-6.5	-6.2	0.1
**2749**	-5.8	-5.9	-5	-5	0.1
**352078**	-4.8	-4.9	-5.7	-4.7	0.1
**2415319**	-5.8	-5.9	-5.7	-4.8	0.1
**2948477**	-5.8	-5.9	-4.9	-5.2	0.1
**6098**	-5.9	-6.1	-5.4	-5	0.2
**56763769**	-3.6	-3.8	-4.2	-3.6	0.2
**25429**	-5.1	-5.3	-5.3	-4.9	0.2
**130313**	-4.5	-4.7	-5.4	-4.5	0.2
**465951**	-5.5	-5.7	-5.6	-5.1	0.2
**3008319**	-6.3	-6.5	-5.8	-5.7	0.2
**86298528**	-7.2	-7.4	-6.3	-5.7	0.2
**135413525**	-5.6	-5.8	-6.1	-5.3	0.2
**10324367**	-6	-6.2	-5.5	-5	0.2
**3366**	-3.8	-4	-4.5	-3.9	0.2
**135976563**	-7.1	-7.3	-6.5	-5.8	0.2
**2392**	-7.4	-7.7	-6.8	-7.2	0.3
**100492**	-6.3	-6.6	-5.9	-5.7	0.3
**14570151**	-5.4	-5.7	-5.2	-5.5	0.3
**3047730**	-4.3	-4.6	-4.7	-4.1	0.3
**10543588**	-6.9	-7.2	-6.7	-6.1	0.3
**11647548**	-6.2	-6.5	-6.2	-5.5	0.3
**44185380**	-5.8	-6.1	-5.9	-5.3	0.3
**65781**	-6.1	-6.4	-5.9	-5.3	0.3
**12000240**	-6.1	-6.4	-6.1	-5.8	0.3
**44563024**	-7.1	-7.4	-7.4	-6.7	0.3
**6918462**	-5.9	-6.3	-6.2	-5.2	0.4
**9835049**	-4.4	-4.8	-5.1	-4.7	0.4
**76336146**	-3.9	-4.3	-5.3	-4.3	0.4
**36294**	-4.8	-5.2	-5	-4.7	0.4
**446598**	-5.2	-5.7	-5.4	-5	0.5
**464205**	-4.3	-4.8	-4.7	-4.9	0.5
**66575905**	-6.4	-6.9	-5.7	-5.7	0.5
**91885568**	-7.2	-7.7	-6.4	-6.5	0.5
**10955174**	-5.1	-5.6	-5.2	-4.9	0.5
**493400**	-7	-7.7	-5.8	-5.8	0.7
**71456558**	-7.8	-8.5	-7.3	-7.4	0.7
**73305**	-3.8	-4.5	-4.5	-3.5	0.7
**25332**	-4	-5.2	-5.1	-4.1	1.2
**5071**	-4.4	-5.8	-5.3	-4.5	1.4

```
  EcDnaK ------GKIIGIDLGTTNSCVAIMDGTTPRVLENAEGDRTTPSIIAYTQDGCTLVGQPAKRQAVTNPQNTLFAIKRLIGR 75
  HsGRP78 -----VGTVVGIDLGTTYSCVGVFKNGRVEIIANDQGNRITPSYVAFTPEGERLIGDAAKNQLTSNPENTVFDAKRLIGR 101
PfHsp70-2 -----EGPVIGIDLGTTYSCVGVFKNGRVEILNNELGNRITPSYVSFV-DGERKVGEAAKLEATLHPTQTVFDVKRLIGR 100
  HsHsp70 -----KAAAIGIDLGTTYSCVGVFQHGKVEIIANDQGNRTTPSYVAFT-DTERLIGDAAKNQVALNPQNTVFDAKRLIGR 76
PfHsp70-x NNAEESEVAIGIDLGTTYSCVGICRNGVVDIIANDQGNRTTPSYVAFT-DTERLIGDAAKNQASRNPENTVFDAKRLIGR 105
Consensus          :******* ***.:       :: *  *:* *** :::. :    *:* ** :   :* :*:*  ******
Conserved

   EcDnaK RFQDEEVQRDVSIMPFKIIAADNGDAWVEVK-----GQKMAPPQISAEVLKKMKKTAEDYLGEPVTEAVITVPAYFNDAQ 150
  HsGRP78 TWNDPSVQQDIKFLPFKVVEK-KTKPYIQVDIGGGQTKTFAPEEISAMVLTKMKETAEAYLGKKVTHAVVTVPAYFNDAQ 180
PfHsp70-2 KFDDQEVVKDRSLLPYEIVNN-QGKPNIKVQIKDK-DTTFAPEQISAMVLEKMKEIAQSFLGKPVKNAVVTVPAYFNDAQ 178
  HsHsp70 KFGDPVVQSDMKHWPFQVIND-GDKPKVQVSYKGE-TKAFYPEEISSMVLTKMKEIAEAYLGYPVTNAVITVPAYFNDSQ 154
PfHsp70-x KFSETTVQSDMKHWPFTVKGGSDGKPMIEVSYQGE-KKTFHPEEISSMVLKKMKEVAETYLGKPVKNAVITVPAYFNDSQ 184
Consensus  : :  *  * .  *: :      .  ::*.        : * :**: ** ***: *: :**  *..**:********:*
Conserved

   EcDnaK RQATKDAGRIAGLEVKRIINEPTAAALAYGLDKG-TGNRTIAVYDLGGGAFDISIIEIDEVDGEKTFEVLATNGDTHLGG 229
  HsGRP78 RQATKDAGTIAGLNVMRIINEPTAAAIAYGLDKR-EGEKNILVFDLGGGTFDVSLLTIDN----GVFEVVATNGDTHLGG 255
PfHsp70-2 RQATKDAGTIAGLNIVRIINEPTAAALAYGLDKKE--ETSILVYDLGGGTFDVSILVIDN----GVFEVYATAGNTHLGG 252
  HsHsp70 RQATKDAGVIAGLNVLRIINEPTAAAIAYGLDRTGKGERNVLIFDLGGGTFDVSILTIDD----GIFEVKATAGDTHLGG 230
PfHsp70-x RQATKDAGAIAGLNVLRIINEPTAAAIAYGLDKKGKGEQNILIFDLGGGTFDVSLLTLED----GIFEVKATSGDTHLGG 260
Consensus ******** ****:: **********:*****:    : .: ::*****:**:*:: :::      *** ** *:*****
Conserved                                                         E DN D     E
                                                                  T ED
                                                                  V

   EcDnaK EDFDSRLINYLVEEFKKDQ-GIDLRNDPLAMQRLKEAAEKAKIELSSAQQTDVNLPYITADATGPKHMNIKVTRAKLESL 308
  HsGRP78 EDFDQRVMEHFIKLYKKKT-GKDVRKDNRAVQKLRREVEKAKRALSSQHQARIEIESFY----EGEDFSETLTRAKFEEL 330
PfHsp70-2 EDFDQRVMDYFIKMFKKKN-NIDLRTDKRAIQKLRKEVEIAKRNLSVVHSTQIEIEDIV----EGHNFSETLTRAKFEEL 327
  HsHsp70 EDFDNRLVNHFVEEFKRKH-KKDISQNKRAVRRLRTACERAKRTLSSSTQASLEIDSLF----EGIDFYTSITRARFEEL 305
PfHsp70-x EDFDNKLVNFCVQDFKKKNGGKDVSKNSKSLRRLRTQCEKAKRVLSSSAQATIEVDSLF----DGIDYNVNITRAKFEEL 336
Consensus ****.::::. :: :*:.   :*   :  ::::*:   * **  **   .: :::  :        .   .:***::*.*
Conserved

   EcDnaK VEDLVNRSIEPLKVALQDAGLSVSDIDDVILVGGQTRMPMVQKKVAEFF-GKEPRKDVNPDEAVAIGAAVQGGVLTGD-- 385
  HsGRP78 NMDLFRSTMKPVQKVLEDSDLKKSDIDEIVLVGGSTRIPKIQQLVKEFFNGKEPSRGINPDEAVAYGAAVQAGVLSG--- 407
PfHsp70-2 NDDLFRETLEPVKKVLDDAKYEKSKIDEIVLVGGSTRIPKIQQIIKEFFNGKEPNRGINPDEAVAYGAAIQAGIILG--- 404
  HsHsp70 CSDLFRSTLEPVEKALRDAKLDKAQIHDLVLVGGSTRIPKVQKLLQDFFNGRDLNKSINPDEAVAYGAAVQAAILMG--- 382
PfHsp70-x CMDQFRNTLIPVEKVLKDAKMDKSQVHEIVLVGGSTRIPKIQQLIKDFFNGKEPCKAINPDEAVAYGAAVQAAILSGDQS 416
Consensus   * .. :: *:: .* *:  . :.:.:::****.**:* :*: : :** *::  : :******* ***:*..:: *
Conserved
```

Figure S3. Multiple sequence alignment of the NBDs of Hsp70 proteins. The proteins are defined by their common name in the first column. Colored in black are identical amino acids (in at least 50% of the aligned sequences), colored in light grey are similar amino acids (in at least 50% of the aligned sequences), and colored in white are the amino acids with no identity or similarity. The default categories for similar amino acids were applied to the multiple sequence alignment (ILV, FWY, KRH, DE, GAS, P, C and TNQM). The row titled "Consensus" are the common consensus symbols of the multiple sequence alignment: an "*" (asterisk) indicates positions which have a single, fully conserved residue; a ":" (colon) indicates conservation between groups of strongly similar properties; and a "." (period) indicates conservation between groups of weakly similar properties. The row titled "Conserved" refers to residues previously found to be highly conserved across NBD domains of different origins, with the residues defined at the bottom of the alignment (Osipiuk et al., 1999; Hughes et al., 2016; Chen et al., 2018; Kityk et al. 2018; Day et al., 2019). The alignment was created using Clustal Omega (Sievers et al., 2017) and rendered with box shading using Multiple Align Show (Stothard, 2000).

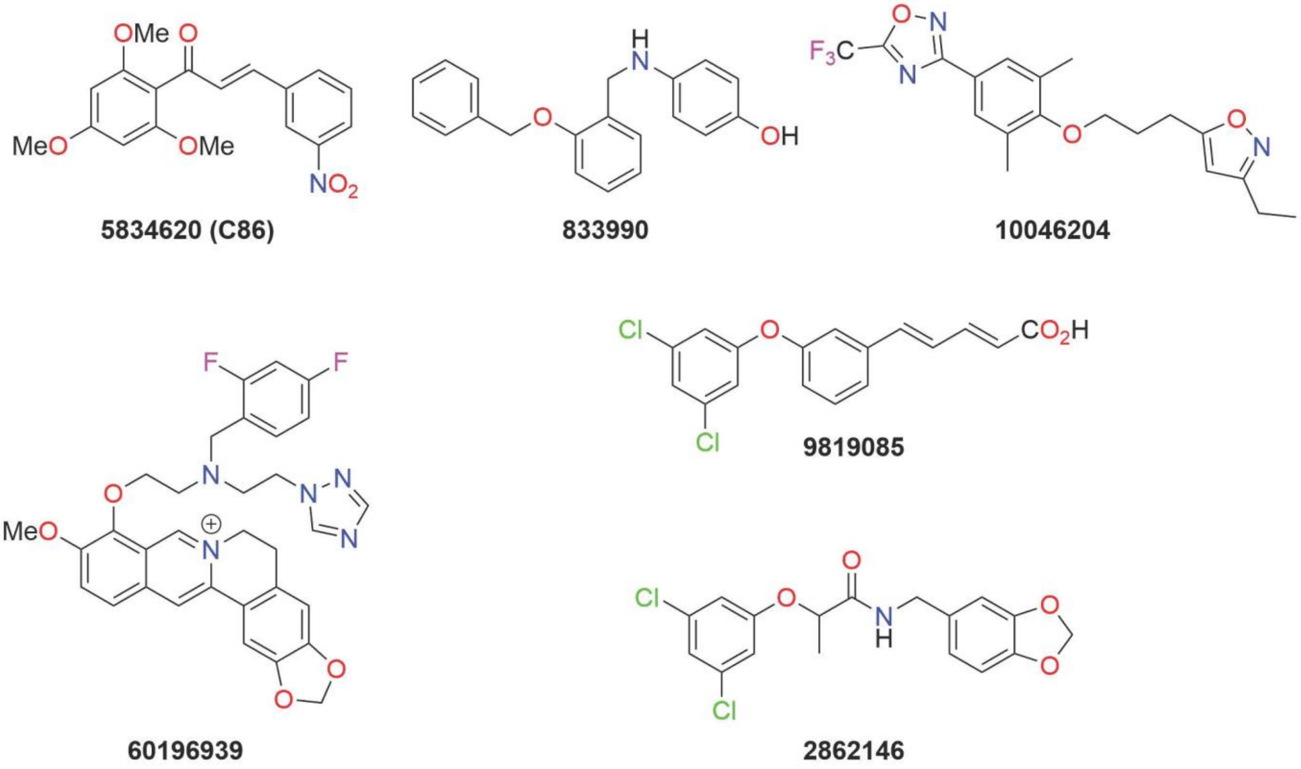

Figure S4. Chemical structures of compounds exhibiting differential binding affinity for PfHsp70-x-PFE0055c over PfHsp70-x-DNAJA1, and the reference compound C86. Pubchem Compound IDs (CIDs) for each compound are indicated below each structure in bold type. These structures have been retrieved from the Pubchem database (https://pubchem.ncbi.nlm.nih.gov/).

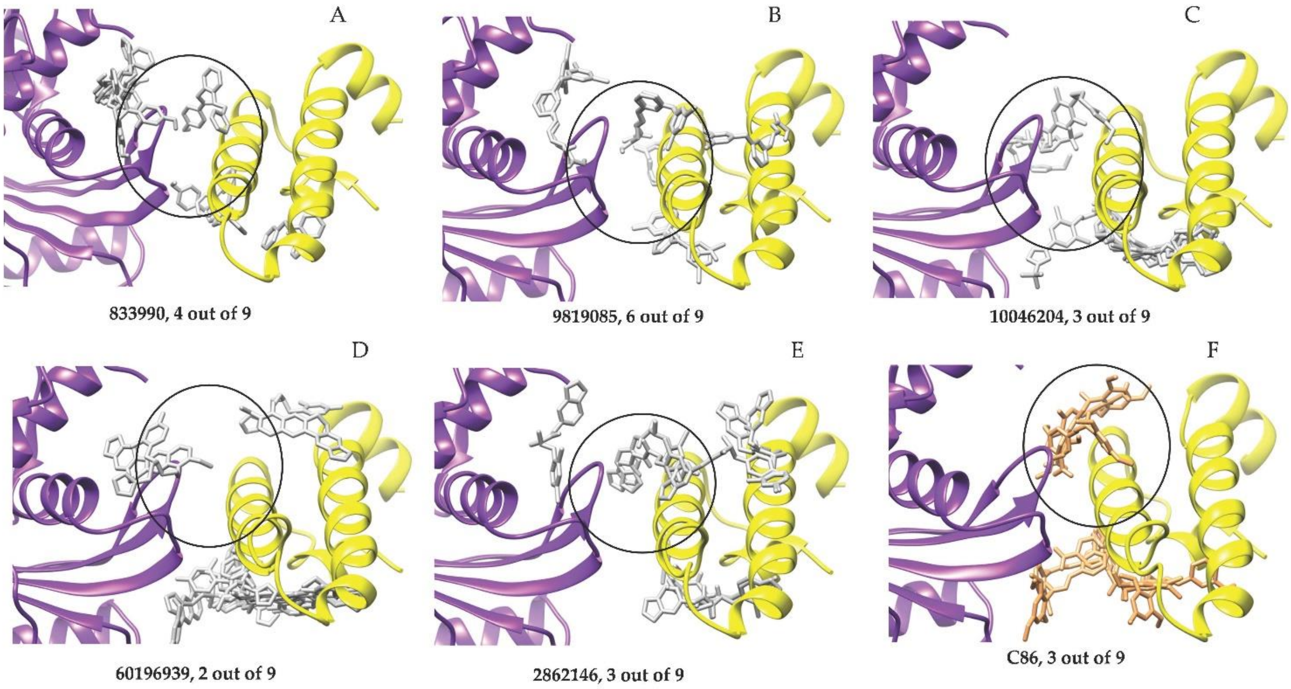

Figure S5. Results of docking into the interface of the PfHsp70-x NBD (purple) and PFE0055c J domain (yellow). The compounds (A-E; grey color) and the reference C86 (sandy brown color) are represented as sticks. The interface region is marked by a black circle. The caption for each compound shows its Pubchem CIDs and the number of poses docked into the interface region out of the total number of poses.

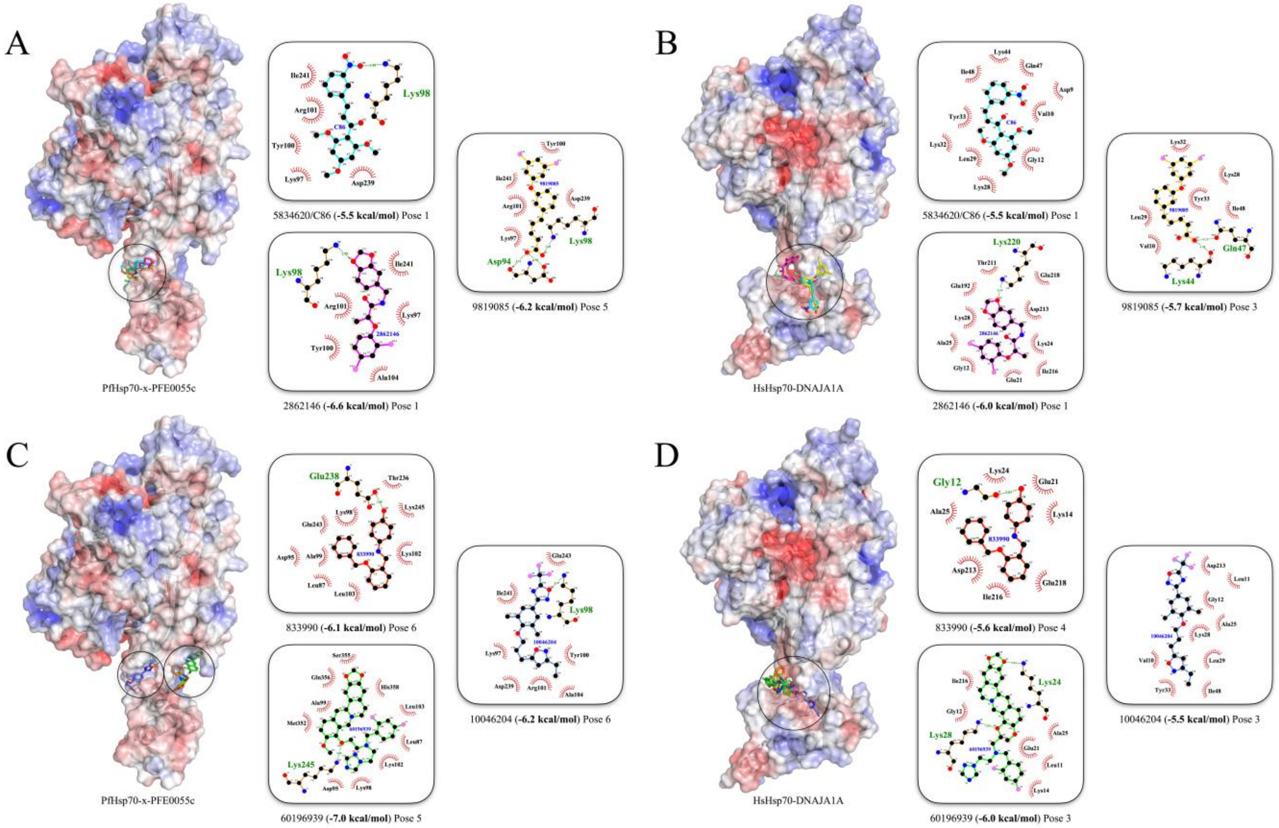

Figure S6. 3D structure molecular docking of (A) and (C) Hsp70-DNAJA1 (B) and (D) PfHsp70-x-PFE0055c in complex with 5834620/C86, 2862146/MBX 1641, 9819085, 833990, 60196939 and 10046204. The protein structures show the docked 5834620/C86 ligand as cyan sticks, 2862146/MBX 1641 as pink sticks, 9819085 as yellow sticks, 833990 as orange sticks, 60196939 as green sticks and 10046204 as sky blue sticks. The positive charge is shown in blue colored surface, negative charge shown in red colored surface and neutral potentials shown in white colored surface. The surface electrostatic potential calculated by APBS and graphically rendered using PyMol 2.5.2 (PyMOL Molecular Graphics System, Version 2.0 Schrödinger, LLC). The contact analysis on the right side of each protein structure shows the ligand-protein interaction diagrams. The 5834620/C86 ligand bonds are shown with thick cyan lines, 2862146/MBX 1641 ligand bonds are shown with thick pink lines, 9819085 ligand bonds are shown with thick yellow lines, 833990 ligand bonds are shown with thick orange lines, 60196939 ligand bonds are shown with thick green lines,10046204 ligand bonds are shown with thick sky blue lines, non-ligand bonds belonging to protein residues to which the ligand is hydrogen-bonded are shown with thin gold bonds, nitrogen and oxygen are highlighted with blue and red colors dots, respectively, hydrogen bonds are shown by green dashed lines with the length of the bond printed in the middle and hydrophobic contacts between protein and ligand are indicated by the brick-red spoked arcs. The plots were generated by LigPlot+ (Laskowski and Swindells, 2011).

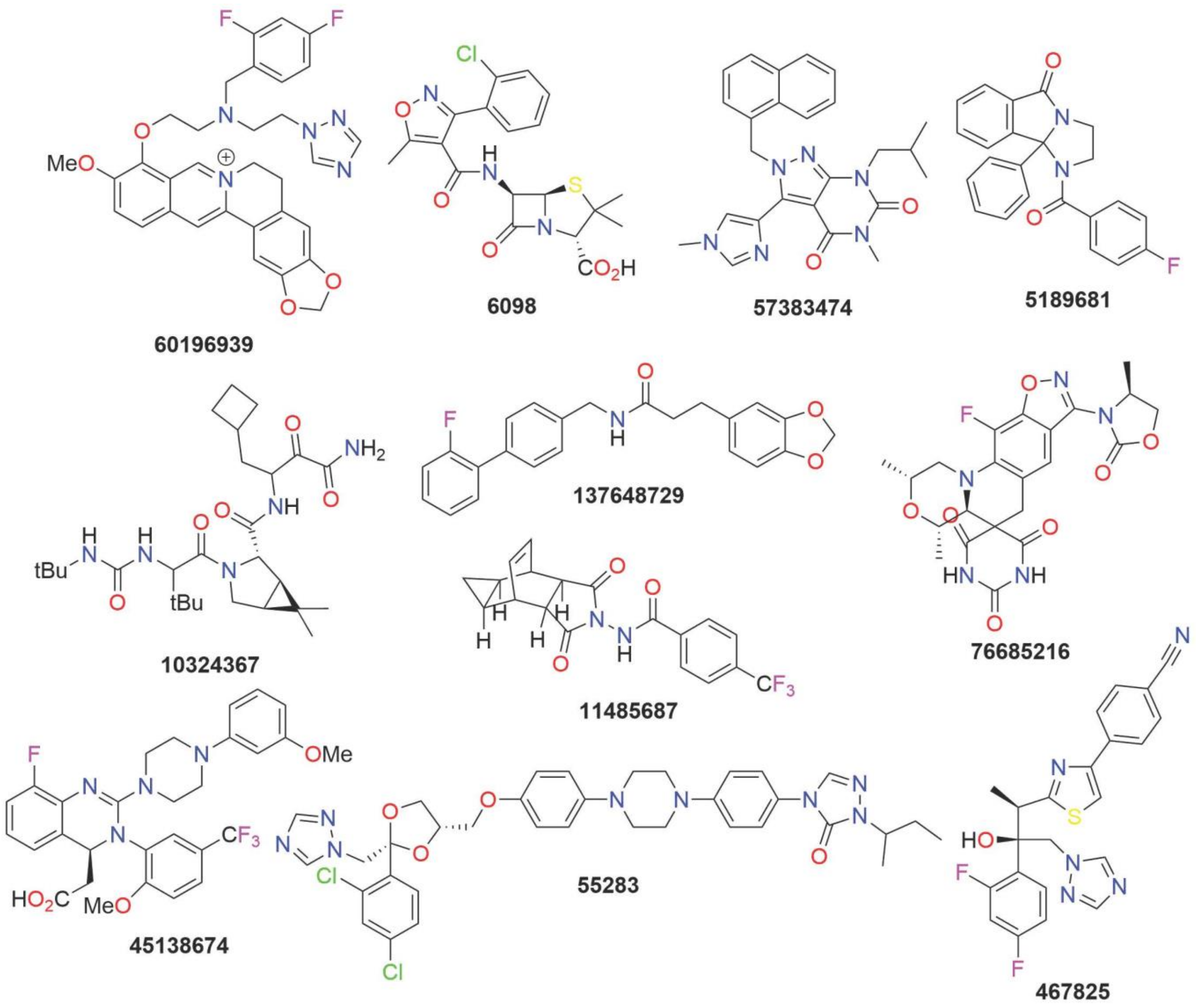

Figure S7. Chemical structures of the top docking solutions for the ER complexes PfHsp70-2-PfSec63 and HsGRP78-HsSec63. Pubchem CIDs for each compound have been mentioned at the bottom in bold type. These structures have been retrieved from the Pubchem database (https://pubchem.ncbi.nlm.nih.gov/).

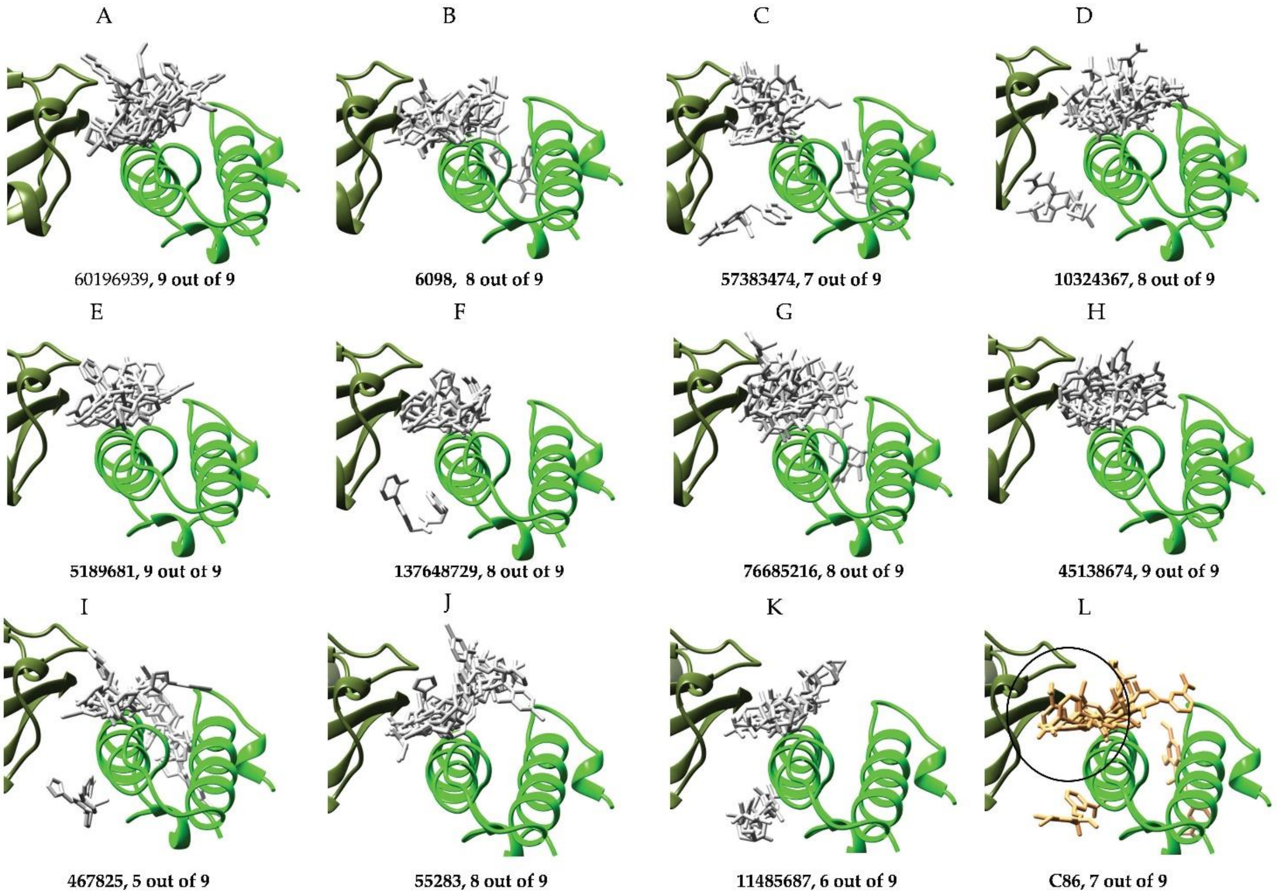

Figure S8. Results of docking into the interface between PfHsp70-2 (olive green) and PfSec63 (green). Compounds (A-I; grey color) and the reference C86 (sandy brown color) are represented as sticks. The interface region is marked by a black circle for C86. The caption for each compound shows its Pubchem CIDs and the number of poses docked into the interface region out of total number of poses.

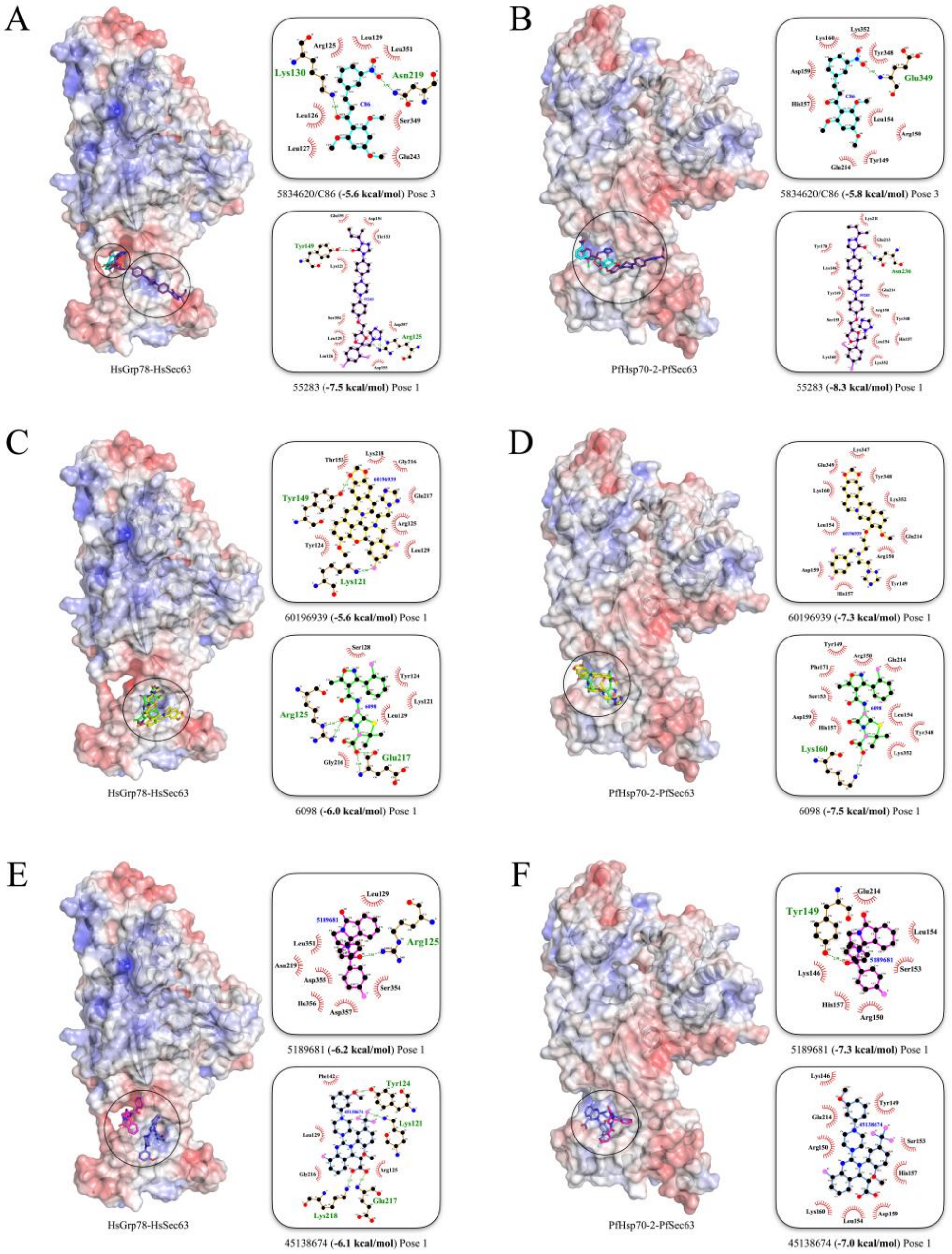

Figure S9. 3D structure molecular docking of (A), (C) and (E) HsGRP78-HsSec63 (B), (D) and (F) PfHsp70-2-PfSec63 in complex with 5834620/C86, 55283/itraconazole, 60196939, 6098, 5189681 and 45138674. The protein structures show the docked 5834620/C86 ligand as cyan sticks, 55283 as purple sticks, 60196939 as yellow sticks, 6098 as green sticks, 5189681 as pink sticks and 45138674 as sky blue sticks. The positive charge is shown in blue colored surface, negative charge shown in red colored surface and neutral potentials shown in white colored surface. The surface electrostatic potential calculated by APBS and graphically rendered using PyMol 2.5.2 (PyMOL Molecular Graphics System, Version 2.0 Schrödinger, LLC). The contact analysis on the right side of each protein structure shows the ligand-protein interaction diagrams. Ligands bonds are shown with thick colored lines that correspond with the sticks colors, non-ligand bonds belonging to protein residues to which the ligand is hydrogen-bonded are shown with thin gold bonds, nitrogen and oxygen are highlighted with blue and red colors dots, respectively, hydrogen bonds are shown by green dashed lines with the length of the bond printed in the middle and hydrophobic contacts between protein and ligand are indicated by the brick-red spoked arcs. The plots were generated by LigPlot+ (Laskowski and Swindells, 2011).

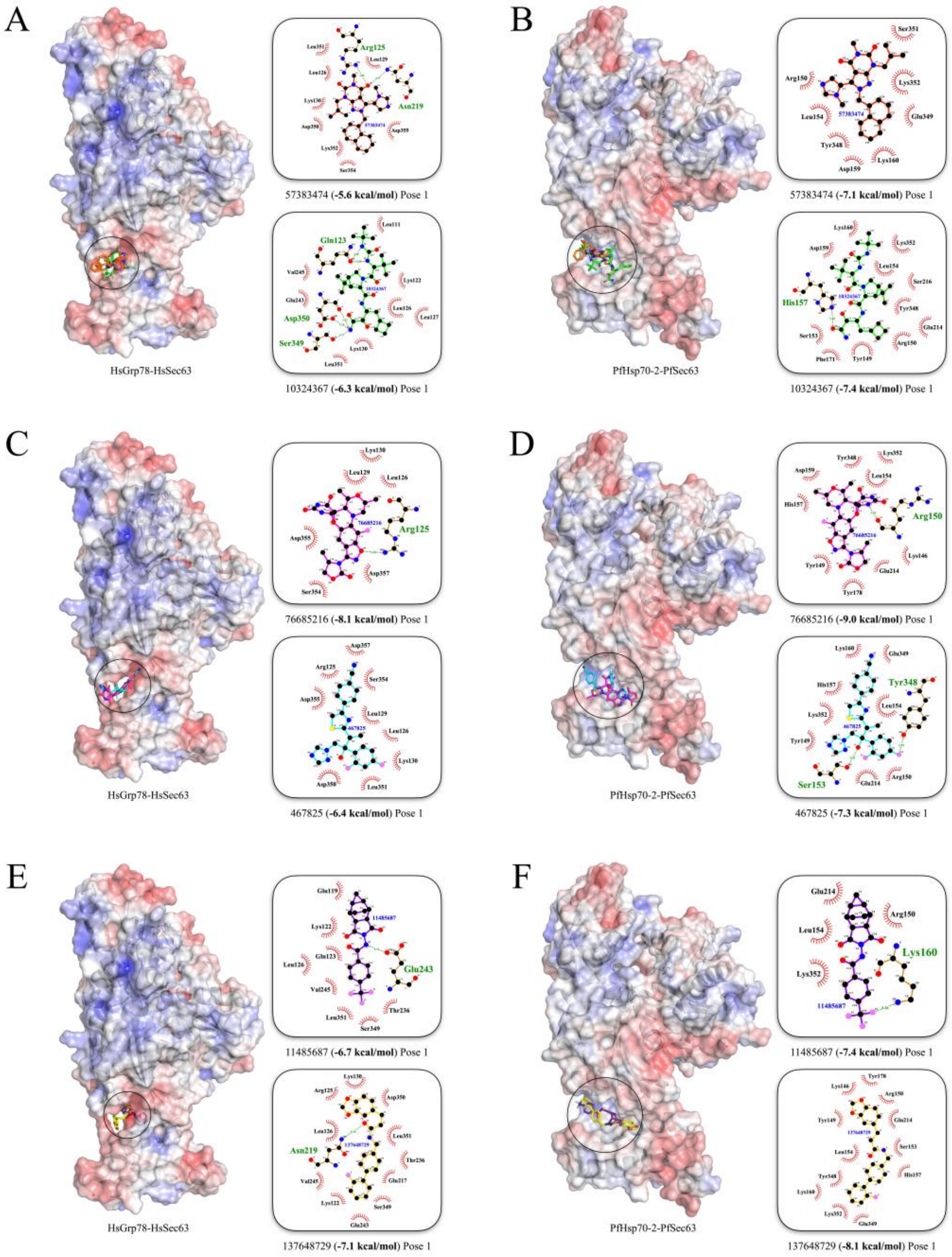

Figure S10. 3D structure molecular docking of (A), (C) and (E) HsGRP78-HsSec63 (B), (D) and (F) PfHsp70-2-PfSec63in complex with 57383474, 10324367, 76685216/zoliflodacin, 467825, 11485687 and 137648729. The protein structures show the docked 57383474 ligand as orange sticks, 10324367 as green sticks, 76685216 as pink sticks, 467825 as cyan sticks, 11485687 as purple sticks and 137648729 as yellow sticks. The positive charge is shown in blue colored surface, negative charge shown in red colored surface and neutral potentials shown in white colored surface. The surface electrostatic potential calculated by APBS and graphically rendered using PyMol 2.5.2 (PyMOL Molecular Graphics System, Version 2.0 Schrödinger, LLC). The contact analysis on the right side of each protein structure shows the ligand-protein interaction diagrams. Ligands bonds are shown with thick colored lines that correspond with the sticks colors, non-ligand bonds belonging to protein residues to which the ligand is hydrogen-bonded are shown with thin gold bonds, nitrogen and oxygen are highlighted with blue and red colors dots, respectively, hydrogen bonds are shown by green dashed lines with the length of the bond printed in the middle and hydrophobic contacts between protein and ligand are indicated by the brick-red spoked arcs. The plots were generated by LigPlot+ (Laskowski and Swindells, 2011).

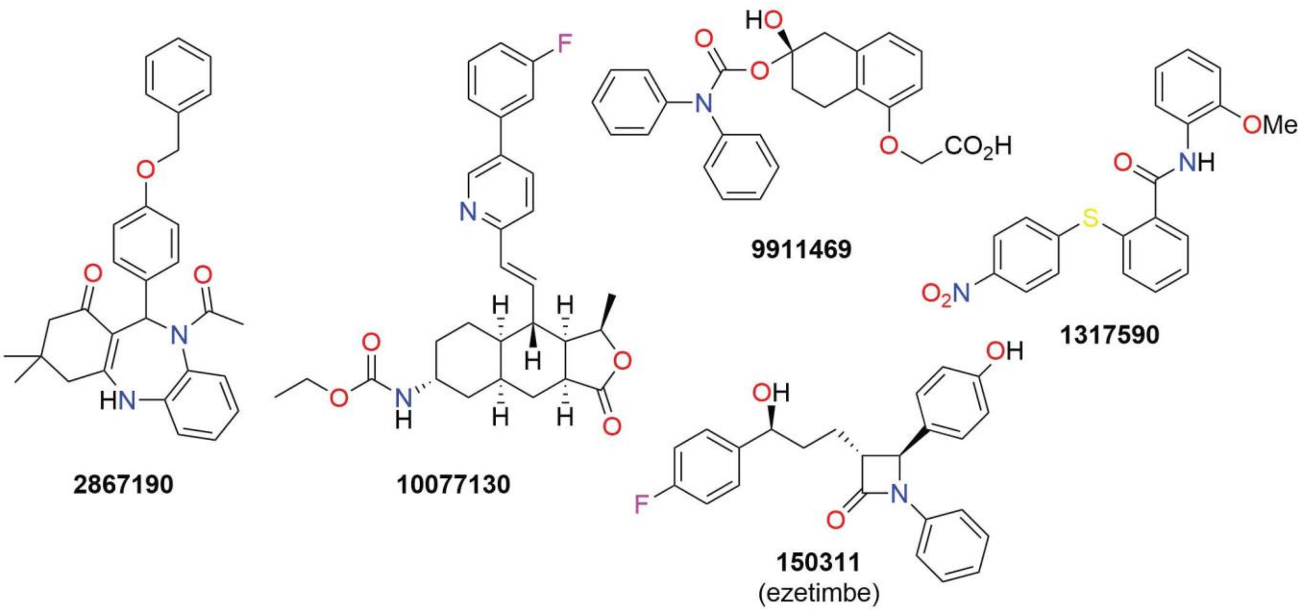

Figure S11. Chemical structures of the compounds with differential binding affinity across cytosolic (PFE0055c) and ER (PfSec63) J domains. Pubchem CIDs for each compound have been mentioned at the bottom in bold type. These structures have been retrieved from the Pubchem database (https://pubchem.ncbi.nlm.nih.gov/).

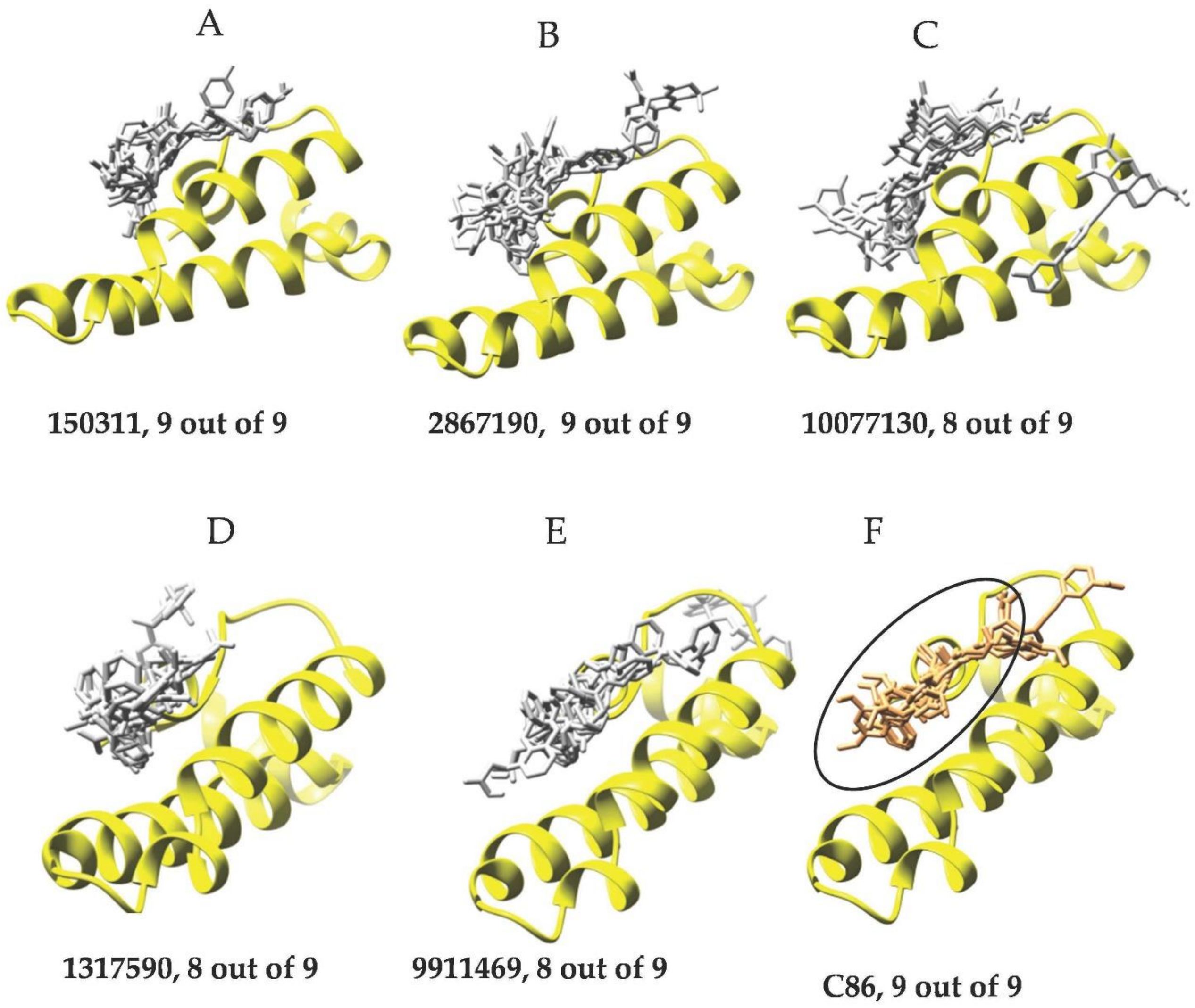

Figure S12. Results of Docking into the helix II region of the PFE0055c J domain (yellow). The compounds (A-E; grey color) and the reference C86 (sandy brown color) are represented as sticks. The helix II region is marked by a black circle for C86. The caption for each compound shows its Pubchem CIDs and the number of poses docked into the interface region out of the total number of poses.

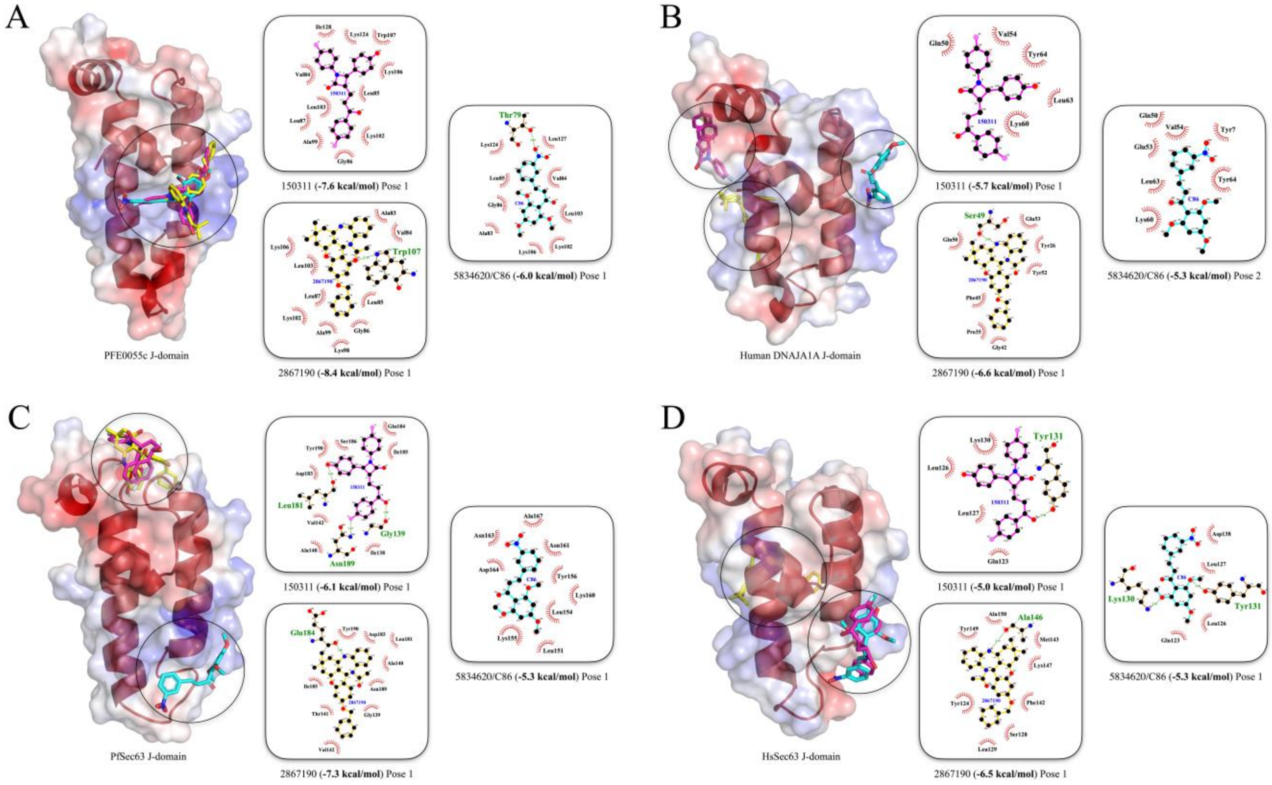

Figure S13. 3D structure molecular docking of (A) PFE0055c J-domain (B) Human DNAJA1 J-domain (C) PfSec63 and (D) human Sec63 J-domain in complex with 5834620/C86, 150311 and 2867190. The protein structures show the docked 5834620/C86 ligand as cyan sticks, 150311 as pink sticks and 2867190 as yellow sticks. The positive charge is shown in blue colored surface, negative charge shown in red colored surface and neutral potentials shown in white colored surface. The surface electrostatic potential calculated by APBS and graphically rendered using PyMol 2.5.2 (PyMOL Molecular Graphics System, Version 2.0 Schrödinger, LLC). The contact analysis on the right side of each protein structure shows the ligand-protein interaction diagrams. Ligands bonds are shown with thick colored lines that correspond with the sticks colors, non-ligand bonds belonging to protein residues to which the ligand is hydrogen-bonded are shown with thin gold bonds, nitrogen and oxygen are highlighted with blue and red colors dots, respectively, hydrogen bonds are shown by green dashed lines with the length of the bond printed in the middle and hydrophobic contacts between protein and ligand are indicated by the brick-red spoked arcs. The plots were generated by LigPlot+ (Laskowski and Swindells, 2011).

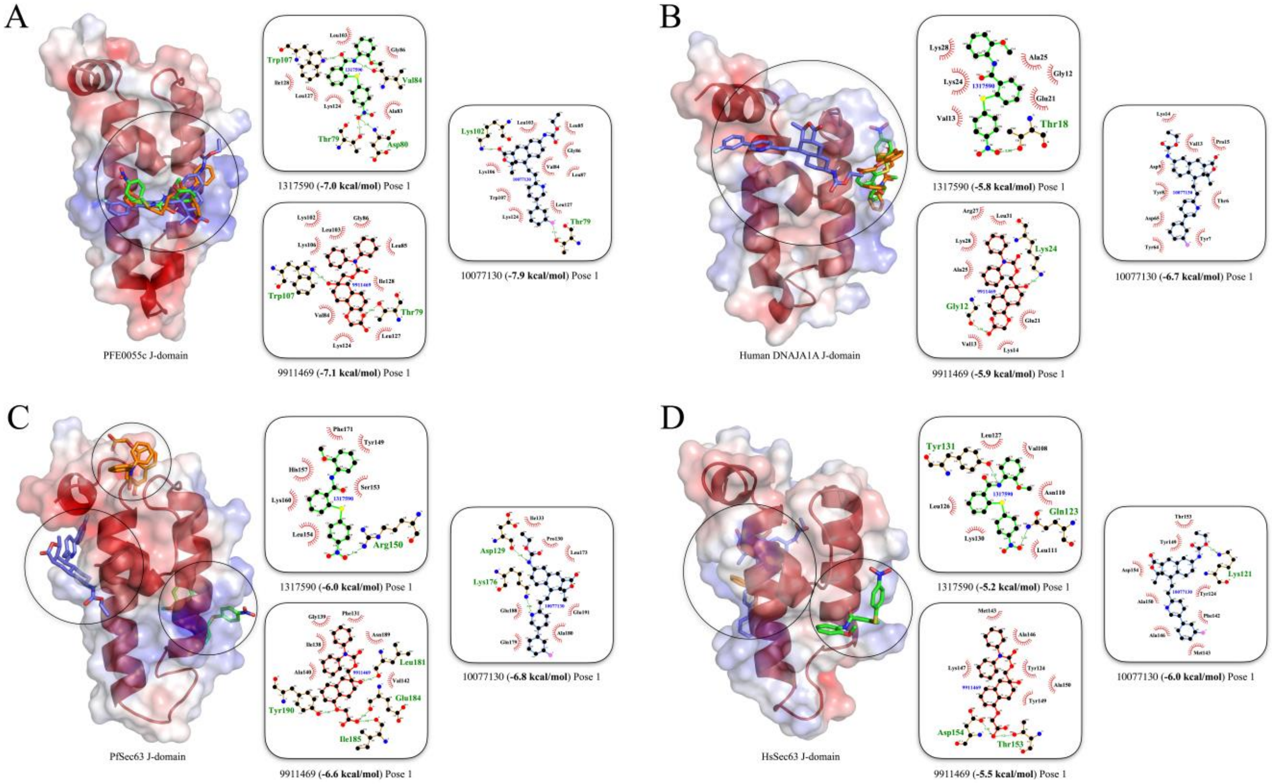

Figure S14. 3D structure molecular docking of (A) PFE0055c J-domain (B) Human DNAJA1 J-domain (C) PfSec63 and (D) human Sec63 J-domain in complex with 1317590, 9911469 and 10077130. The protein structures show the docked 1317590 ligand as green sticks, 9911469 as orange sticks and 10077130 as sky blue sticks. The positive charge is shown in blue colored surface, negative charge shown in red colored surface and neutral potentials shown in white colored surface. The surface electrostatic potential calculated by APBS and graphically rendered using PyMol 2.5.2 (PyMOL Molecular Graphics System, Version 2.0 Schrӧdinger, LLC). The contact analysis on the right side of each protein structure shows the ligand-protein interaction diagrams. Ligands bonds are shown with thick colored lines that correspond with the sticks colors, non-ligand bonds belonging to protein residues to which the ligand is hydrogen-bonded are shown with thin gold bonds, nitrogen and oxygen are highlighted with blue and red colors dots, respectively, hydrogen bonds are shown by green dashed lines with the length of the bond printed in the middle and hydrophobic contacts between protein and ligand are indicated by the brick-red spoked arcs. The plots were generated by LigPlot+ (Laskowski and Swindells, 2011).

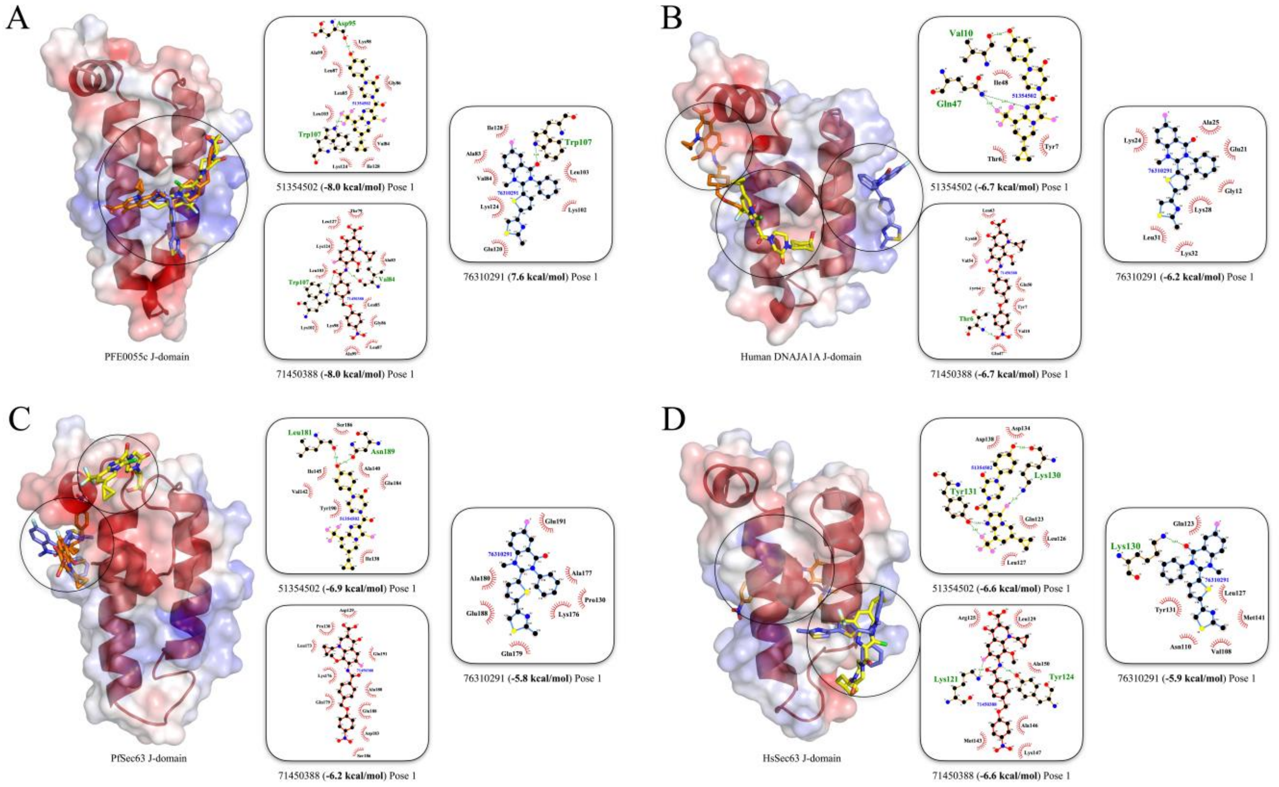

Figure S15. 3D structure molecular docking of (A) PFE0055c J-domain (B) Human DNAJA1 J-domain (C) PfSec63 and (D) human Sec63 J-domain in complex with 51354502, 71450388 and 76310291. The protein structures show the docked 51354502 ligand as yellow sticks, 71450388 as orange sticks and 76310291 as sky blue sticks. The positive charge is shown in blue colored surface, negative charge shown in red colored surface and neutral potentials shown in white colored surface. The surface electrostatic potential calculated by APBS and graphically rendered using PyMol 2.5.2 (PyMOL Molecular Graphics System, Version 2.0 Schrödinger, LLC). The contact analysis on the right side of each protein structure shows the ligand-protein interaction diagrams. Ligands bonds are shown with thick colored lines that correspond with the sticks colors, non-ligand bonds belonging to protein residues to which the ligand is hydrogen-bonded are shown with thin gold bonds, nitrogen and oxygen are highlighted with blue and red colors dots, respectively, hydrogen bonds are shown by green dashed lines with the length of the bond printed in the middle and hydrophobic contacts between protein and ligand are indicated by the brick-red spoked arcs. The plots were generated by LigPlot+ (Laskowski and Swindells, 2011).

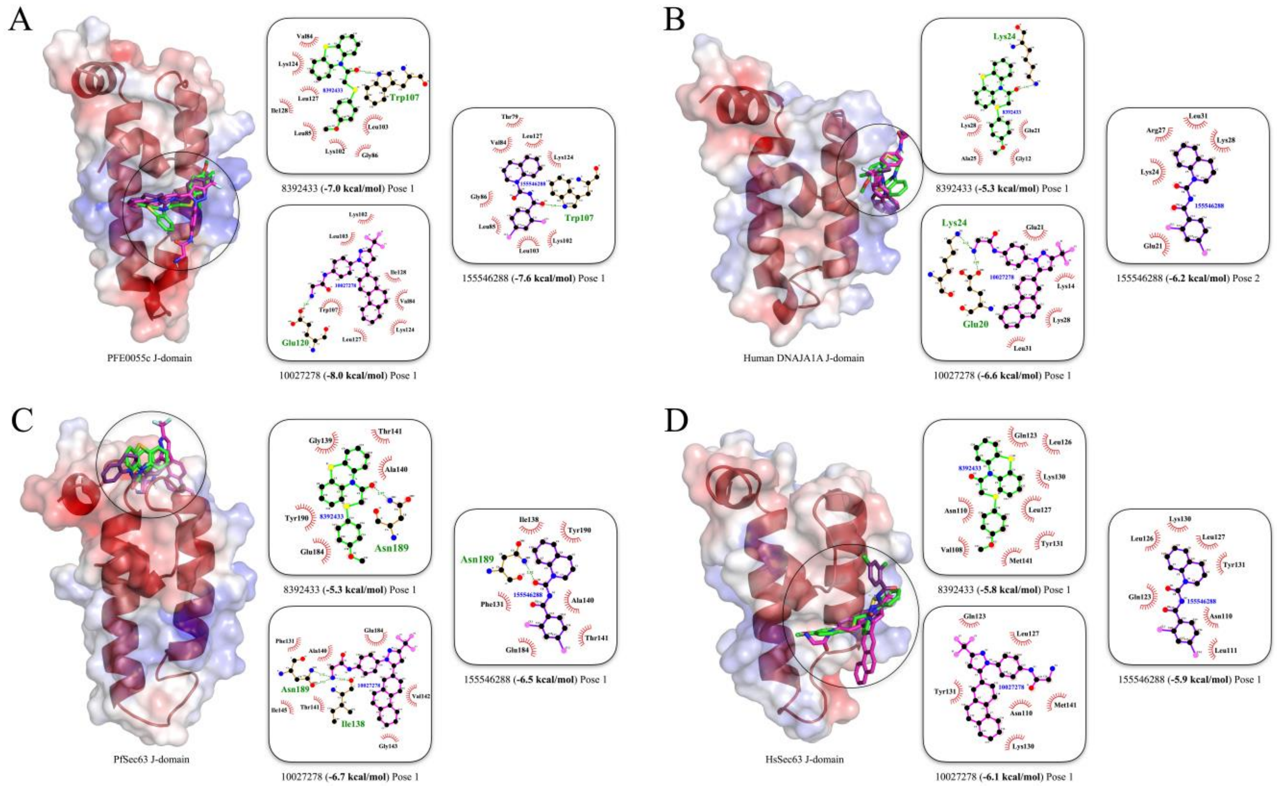

Figure S16. 3D structure molecular docking of (A) PFE0055c J-domain (B) Human DNAJA1 J-domain (C) PfSec63 and (D) human Sec63 J-domain in complex with 8392433, 10027278 and 155546288. The protein structures show the docked 8392433 ligand as green sticks, 10027278 as pink sticks and 155546288 as purple sticks. The positive charge is shown in blue colored surface, negative charge shown in red colored surface and neutral potentials shown in white colored surface. The surface electrostatic potential calculated by APBS and graphically rendered using PyMol 2.5.2 (PyMOL Molecular Graphics System, Version 2.0 Schrödinger, LLC). The contact analysis on the right side of each protein structure shows the ligand-protein interaction diagrams. Ligands bonds are shown with thick colored lines that correspond with the sticks colors, non-ligand bonds belonging to protein residues to which the ligand is hydrogen-bonded are shown with thin gold bonds, nitrogen and oxygen are highlighted with blue and red colors dots, respectively, hydrogen bonds are shown by green dashed lines with the length of the bond printed in the middle and hydrophobic contacts between protein and ligand are indicated by the brick-red spoked arcs. The plots were generated by LigPlot+ (Laskowski and Swindells, 2011).

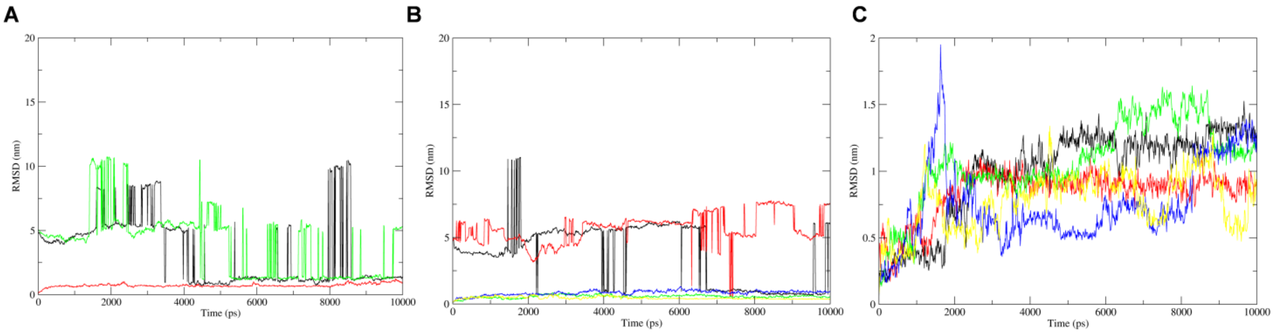

Figure S17. Root mean square deviation (RMSD) of the ligand structure (heavy atoms) measured in nm fitted to the protein backbone over the MD simulation time period (ps). A: Exported Hsp70-JDP complexes, PfHsp70-x-PFE0055c-MBX 1641 (black), HsHsp70-DNAJA1-MBX 1641 (red) and PfHsp70-x-PFE0055c-C86 (green). B: ER Specific Hsp70-JDP complexes, PfHsp70-2-PfSec63-itraconazole (green), HsGRP78-HsSec63-itraconazole (black), PfHsp70-2-PfSec63-zoliflodacin (blue), HsGRP78-HsSec63-zoliflodacin (red) and PfHsp70-2-PfSec63- C86 (yellow). C: PFE0055c J Domain-ezetimibe (black), DNAJA1 J Domain-ezetimibe (red), PFE0055c J Domain-benzo-diazepinone (green), DNAJA1 J Domain-benzo-diazepinone (blue), PFE0055c J Domain-C86 (yellow).

Supplementary Table S16. A detailed decomposition of binding energy obtained through MMPBSA analysis of the top ranked compounds into various components.

Frames	VDWAALS	EEL	EPB	ENPOLAR	GGAS	GSOLV	TOTAL
**PfHsp70-x-PFE0055c –MBX 1641**							
Average	-21.46	-10.01	21.14	-2.4	-31.47	18.74	-12.73
SD	6.44	7.79	8.92	0.67	10.49	8.55	4.15
SEM	0.3	0.37	0.42	0.03	0.49	0.4	0.2
**HsHsp70-DNAJA1 –MBX 1641**							
Average	-28.15	-45.24	59.12	-2.9	-73.39	56.22	-17.17
SD	4.35	10.05	11.69	0.41	12.63	11.37	3.58
SEM	0.21	0.47	0.55	0.02	0.6	0.54	0.17
**PfHsp70-x-PFE0055c –C86**							
Average	-18.11	-5.86	16.86	-2.37	-23.97	14.49	-9.48
SD	8.1	5.88	8.8	1.02	11.44	7.99	4.63
SEM	0.38	0.28	0.41	0.05	0.54	0.38	0.22
**PfHsp70-2-PfSec63- Itraconazole**							
Average	-50.84	-11.08	46.24	-5.49	-61.92	40.75	-21.17
SD	6.02	6.35	7.12	0.33	9.09	6.96	6.38
SEM	0.27	0.28	0.32	0.01	0.41	0.31	0.29
**HsGRP78-HsSec63- Itraconazole**							
Average	-32.08	-12.31	31.1	-3.62	-44.38	27.48	-16.9
SD	8.27	10.57	9.73	0.69	11.48	9.55	4.94
SEM	0.39	0.5	0.46	0.03	0.54	0.45	0.23
**PfHsp70-2-PfSec63- Zoliflodacin**							
Average	-35.38	-19.91	42.94	-3.93	-55.28	39.01	-16.28
SD	3.79	7.2	6.62	0.21	6.61	6.54	3.75
SEM	0.18	0.34	0.31	0.01	0.31	0.31	0.18
**HsGRP78-HsSec63- Zoliflodacin**							
Average	-10.7	-4.41	11.42	-1.38	-15.11	10.04	-5.08
SD	6.7	4.62	7.34	0.77	9.2	6.71	3.86
SEM	0.32	0.22	0.35	0.04	0.43	0.32	0.18
**PfHsp70-2-PfSec63- C86**							
Average	-28.25	-29.38	41.36	-3.16	-57.63	38.2	-19.43
SD	6.32	7.38	7.06	0.48	8.57	6.89	4.11
SEM	0.3	0.35	0.33	0.02	0.4	0.33	0.19

**PFE0055c J domain- Ezetimibe**							
Average	-19.64	-11.88	21.48	-2.63	-31.53	18.85	-12.68
SD	3.55	8.77	7.31	0.31	9.05	7.2	3.89
SEM	0.17	0.41	0.34	0.01	0.43	0.34	0.18
**PFE0055c J domain- Benzo-diazepinone**							
Average	-23.19	-10.68	21.65	-2.8	-33.87	18.85	-15.03
SD	3.92	6.29	5.35	0.3	6.46	5.24	3.56
SEM	0.19	0.3	0.25	0.01	0.3	0.25	0.17
**PFE0055c J domain- C86**							
Average	-18.08	-6.59	15.57	-2.16	-24.68	13.41	-11.26
SD	3.29	4.88	5.23	0.27	6.55	5.07	2.63
SEM	0.15	0.23	0.25	0.01	0.31	0.24	0.12
**DNAJA1 J domain- Ezetimibe**							
Average	-19.5	-6.19	16.35	-2.34	-25.69	14.01	-11.68
SD	6.48	6.7	9.14	0.68	11.15	8.72	4.27
SEM	0.31	0.32	0.43	0.03	0.53	0.41	0.2
**DNAJA1 J domain- Benzo-diazepinone**							
Average	-20.98	-15.42	24.04	-2.49	-36.4	21.54	-14.85
SD	3.01	5.73	6.06	0.24	7.26	5.94	3.03
SEM	0.14	0.27	0.29	0.01	0.34	0.28	0.14

Table S17. Swiss ADME pharmacokinetics prediction for the top ranked compounds and clinical phase states according to ChEMBL.

Common Name	Ali Class	Silicos-IT class	GI absorption	BBB permeation	Pgp substrate	CYP1A2 inhibitor	CYP2C19 inhibitor	Synthetic Accessibility	Clinical Phase
Itraconazole	Poorly soluble	Poorly soluble	High	No	Yes	Yes	Yes	5.77	Approved
Ezetimibe	Moderately soluble	Poorly soluble	High	Yes	Yes	No	Yes	3.37	Approved
Zoliflodacin	Soluble	Moderately soluble	Low	No	Yes	No	No	5.36	Phase 3
Benzo-diazepinone	Poorly soluble	Poorly soluble	High	Yes	Yes	No	Yes	4.62	Preclinical
MBX 1641	Moderately soluble	Poorly soluble	High	Yes	No	Yes	Yes	3.04	Preclinical
